# Tsinghua facial expression database – A database of facial expressions in Chinese young and older women and men: Development and validation

**DOI:** 10.1371/journal.pone.0231304

**Published:** 2020-04-15

**Authors:** Tao Yang, Zeyun Yang, Guangzheng Xu, Duoling Gao, Ziheng Zhang, Hui Wang, Shiyu Liu, Linfeng Han, Zhixin Zhu, Yang Tian, Yuqi Huang, Lei Zhao, Kui Zhong, Bolin Shi, Juan Li, Shimin Fu, Peipeng Liang, Michael J. Banissy, Pei Sun

**Affiliations:** 1 Department of Psychology, School of Social Sciences, Tsinghua University, Beijing, China; 2 Food and Agriculture Standardization Institute, China National Institute of Standardization, Beijing, China; 3 Institute of Psychology, Chinese Academy of Sciences, Beijing, China; 4 School of Psychology, Capital Normal University, Beijing, China; 5 Department of Psychology, Goldsmiths, University of London, London, United Kingdom; 6 Tsinghua H^+^ Lab, Tsinghua University, Beijing, China; 7 Tsinghua Brain and Intelligence Lab, Tsinghua University, Beijing, China; Harvard Medical School, UNITED STATES

## Abstract

Perception of facial identity and emotional expressions is fundamental to social interactions. Recently, interest in age associated changes in the processing of faces has grown rapidly. Due to the lack of older faces stimuli, most previous age-comparative studies only used young faces stimuli, which might cause own-age advantage. None of the existing Eastern face stimuli databases contain face images of different age groups (e.g. older adult faces). In this study, a database that comprises images of 110 Chinese young and older adults displaying eight facial emotional expressions (Neutral, Happiness, Anger, Disgust, Surprise, Fear, Content, and Sadness) was constructed. To validate this database, each image was rated on the basis of perceived facial expressions, perceived emotional intensity, and perceived age by two different age groups. Results have shown an overall 79.08% correct identification rate in the validation. Access to the freely available database can be requested by emailing the corresponding authors.

## Introduction

We live in a complex world where we see countless items at any given one time. Faces are one of the most “biologically and socially significant” visual stimuli in the human environment [1]. Emotional facial expression perception is fundamental to successful social interactions, which are important to happiness and health. For example, declines in facial emotion perception have been linked to increased social isolation, loneliness and poor interpersonal relationships [2, 3]. Research on the perception/identification of facial emotional expressions has been a major aspect of the social perception literature, and it is also an important topic across a variety of domains (including clinical, developmental, personality, physiological, social, and cross-cultural psychology).

The notions that emotional facial expressions are innate and universally expressed and perceived was proposed by Darwin [4]. This view has been supported by evidences showing that basic emotions (happiness, anger, fear, sadness, disgust, and surprise) are displayed and recognised across different cultures (including both Western and Eastern cultures) and across literate and preliterate groups [5–10], although it appears that different cultural groups might differ in the level of accuracy for the recognition of specific facial expressions [11, 12] and exhibit different eye scanning patterns during recognition [12, 13]. Previous studies have shown that the basic emotions are reliably displayed, perceived and recognised by Chinese people of different ages [8–10, 14].

The proportion of older people is growing enormously across the world, and interest in age associated changes in the processing of faces has grown rapidly. The general pattern that has emerged is that older adults appear to have declined recognition of negative facial expressions of emotions such as Anger, Sadness, Fear and Surprise [e.g. 15–18, see 19 for a review]. Due to a lack of appropriate stimuli, a general limitation involved in most previous age-comparative studies is that they only used young face stimuli, which could lead to *own-age advantage*: where participants tend to show better performance in perception of own versus other age faces [20, 21]. Therefore, it is possible that declines in performance displayed by older adults in previous studies have been related to the use of young adult actors, which favours young adult participants. Thus, it is crucial to use faces across different ages when comparing face perceptual abilities in different age groups.

To address this issue, some research groups have constructed face databases and related measurements across different age groups in order to eliminate such perceptual bias involved in face perception research [22–24]. Most age-related face perception research have only focused on Western adulthood, thus the face emotional perceptual abilities changes across Eastern adulthood is less understood. Due to ‘other-race bias’ [e.g. 25, 26], Caucasian face databases are not suitable to be used to measure Eastern adults’ face perceptual abilities. Existing East-Asian/Chinese face stimuli databases such as Japanese and Caucasian Facial Expressions of Emotion [JACFEE; 27], Taiwanese Facial Expression Image Database [TFEID; 28] and Chinese Academy of Science—Pose, Expression, Accessories, and Lighting [CAS-PEAL; 29], and mixed-race Carnegie Mellon University multi-Pose, Illumination, and Expression (PIE) database [CMU Multi-PIE database; 30] have made invaluable contribution to the field of face perception/encoding research. However, none of these facial emotional stimuli databases contain facial images of different age groups (e.g. older adult faces), which is crucial to studying Asian/Chinese adults’ face perceptual abilities across the different adult age groups. Thus, we aimed to go beyond existing databases mentioned above by establishing an Eastern Asian/Chinese database of faces with an age range wider than that of any database currently available. In particular, we included adults of age 60 and older in addition to traditional young adult individuals.

In this report we give a detailed description (including image acquisition and processing, validation procedure, analysis and results) of this freely available database. We hope it will be useful across a range of research domains and advances the discoveries of new scientific knowledge in face perception. Access to the database can be requested by emailing the corresponding authors. (Note: researchers must agree to terms of use).

## Methods

### Development of database

#### Face models

The face models were 67 young (*M = 23*.*82* years, *SD =* 4.18 years; age range, 19–35 years; 34 women), and 70 older (*M =* 64.40 years, *SD* = 3.51 years; age range, 60–76 years; 35 women) native-Chinese adults who have interest in acting. In addition, all face models were required 1) to not have tattoos and piercings on their faces; 2) and to be able to read texts. All face model participants were recruited through flyers, adverts or from word of mouth. All face models provided written permission to have photographs taken and distributed to other researchers for the purpose of scientific research (e.g., scientific experiment, publications and presentations). The individuals included in this manuscript has given written informed consent (as outlined in PLOS consent form) to publish their face images. All face model participants gave informed consent prior to beginning the experiment and were fully informed about the experimental procedure. The local ethics committee of Department of Psychology, Tsinghua University approved the study.

#### Image acquisition

Before photo-shooting, participants were asked not to wear colored makeup (such as eyeshadow, blusher and lipstick) that could potentially bias people’s perception of face age. We allowed participants to wear sunscreen and tinted moisturizer which does not disguise their age- or expression- related skin texture. As our requirements were stated during our recruitment adverts, all participants came along with no colored makeup. Glasses and jewelry such as necklaces and earrings were removed before photo-shooting. Photo-shooting sessions took place at the H^+^ Lab of Tsinghua University in a studio specifically set up for this purpose. High-quality coloured digital photographs (4928 × 3264 pixels) were taken with a Nikon D7000 camera that was positioned at a distance of approximately 130 cm in front of the model. In the studio, ceiling lights were kept off, and no camera flash was used. Matt white wall was used as the background. During each photo-shooting session, face model participants were seated at a comfortable chair with viewing distance from a 13 inch Macbook Pro laptop computer (Apple Inc., Cupertino, CA, USA), three professional studio photography spotlights were used for soft and smooth illumination: one was placed on the midline top of the model at a distance of 50 cm, the other two lights were at the height of the models and head lit the model from 45 degrees on each side of the model. Spotlights were softened with white photography umbrellas. After the photo shoot, all the images of each face model were saved, filed and coded according to age, gender, and the targeted facial expressions.

During the photo-shooting, models were required to portray eight facial expressions including Neutral, Happiness (smile with teeth), Anger, Disgust, Surprise, Fear, Content (smile without teeth), Sadness [31, 32]. According to Ekman & Friesen [4], there were two types of facial expression for the happy emotion–happy with a large smile with teeth visible and smile with a subtle smile without showing teeth (which were more referred as “content”). Similar to the Dartmouth Face database, we included both facial expressions and labeled them separately for the convenience of future research use. Each session started with Neutral facial expression, followed by Happiness, Surprise, Disgust, Sadness, Fear, Anger, and Content. In order to effectively elicit models’ facial expressions, a three-phase facial expression eliciting procedure was used by adapting the effective emotion induction methods in previous face database development studies [23, 32], including *scenario induction phase*, *personal event induction phase*, and *controlled facial expression*. It should be noted that, the order of the three induction stages and the order of the target emotions are not randomised across the participants during the recording. Therefore, the fixed sequence of inductions and/or emotions may affect the produced emotional facial expression of each participant.

The *scenario induction phase* is based on the approach used in the study of Dalrymple et al. [32], in which face models were encouraged to imagine some scenarios described verbally by the photo assistant. *The scenario induction phase* aimed to induce the emotional experience of the target emotion. For each emotion, the research/photography assistant described five or six scenarios that would elicit the corresponding emotion (e.g. Happiness: “Imagine that you just won a big lottery”, or Content: “Imagine you are having a piece of delicious cake”), the scenarios were also displayed on the laptop screen at the same time. The scenarios were selected from an online survey where people voted on the top three scenarios from around ten scenarios (gathered from a focused research group) that could best elicit their corresponding emotions. Face models were then instructed to display facial expression by imagining one of the scenarios that could best induce their target emotion. The *personal event induction phase* aimed to induce face models’ target emotion by encouraging them to imagine past personal events that had strongly elicited their own respective emotion [adapted from 23]. During the *controlled facial expression* phase [23], face models were presented the prototypical PoFA facial emotions [31] on the laptop with guidance on how to control facial muscles in order to optimally portray the target emotions. They were also shown prototypical facial expressions displayed by East Asian Face models [JACFEE; 27] for reference. Face models were provided with a mirror to practice.

Throughout the whole photo-shoot, face models were instructed to look directly at camera, the photographer stood behind the camera and the photo/research assistant stood next to the photographer. Photos were taken continuously during each of the induction [*scenario induction*, *personal event induction*, and *controlled facial expression*] phases. Instructions and feedback were given throughout the whole session, until the desired expression was captured. On average, 80 photos were captured for each emotion type for each face model.

#### Image selection and processing

During the image selection, we aimed to choose one or two images for each model that best represented each of the eight facial expressions based on the manual of Ekman & Friesen [4]. First, poor quality images (e.g., blurry, model was out of focus, ambiguous facial expression) were removed from the pool. Then, each remaining photo were assessed by two trained raters on the ‘purity’ and ‘intensity’ of facial expressions [23]. The trained raters had intensively studied the Ekman & Friesen’s [4] manual and they were familiar with the characteristics (e.g. Action Units: AUs) of each of prototypical facial expressions. For each image, two trained raters were asked to judge what type(s) of facial expression was displayed in the image (Anger, Disgust, Fear, Surprise, Neutral, Sadness and Surprise), and how intensive the facial expression was (1 = low, 2 = middle, 3 = high). After this, we chose images that displayed both “pure” and “high-intensity”. An expressions was regarded as “pure” when both raters chose the same type of facial expressions displayed in the image and they did not choose any other expressions (to make sure the displayed facial expression is not ambiguous or blended emotion). An expression was regarded as “high-intensity” when both raters rated “high” on the intensity option. The two most prototypical images for each model that best represented each of the eight facial expressions were chosen for the validation (or one image if the two most prototypical images are highly identical). After selection, a total of 1128 images were chosen for further processing and validation.

Letting naïve Chinese raters judge images that were not preselected by trained raters would be ideal as it can maximumly retain the potential culturally authentic facial movement features of different types of emotional facial expressions. However, in reality it is hard to achieve this as over several hundreds images per face model are needed to be rated by untrained raters, and the repetition of similar facial expression images might result in participants’ boredom and adaptation that can affect the results. Therefore we adopted the pre-selection procedure that was also used in most face database creation validation studies across different cultures and age groups (Matsumoto, 1988; Ebner, Riediger, & Lindenberger, 2010; Dalrymple, Gomez, & Duchaine, 2013), we followed this convention and conducted the validation study in untrained raters (who were not familiar to Facial Action Coding System (FACS) that involve identification of the facial muscle configurations). Only facial images that the emotions were consistently correctly perceived by untrained raters were included in the final database. In other words, our pre-selected images were validated by naïve Chinese raters who were not familiar with FACS to ensure the images do not only fulfill the FACS, but also culturally authentic as they are easily perceived by naïve local people.

The selected images with tilted head positions were straightened and all photos were adjusted to consistent levels of brightness. Photos were retouched by removing prominent details such as spots, moles, and pimples. Image editing was conducted using Adobe Photoshop CS (Adobe Systems Inc., San Jose, California, USA). Finally, the head-size cut-out images were resized to 1800 × 2200 pixel resolution and saved in JPEG format.

### Validation of the database

In order to validate the database and provide future users with the stimuli-related information, each of the 1128 images of faces were rated in terms of *perceived facial expression*, *perceived emotional intensity* and *perceived age* by two groups of young and older raters. None of the raters participated as face models in the photo-shooting sessions.

#### Face rater participants

35 young and 35 older face rater participants took part in the rating task. All face rater participants were native-Chinese adults, with no known history of neurological problems or language-related problems. Face rater Participants also had normal or corrected-to-normal vision. These sampling criteria were in place to ensure that face rater participants were typical adults without any difficulties in understanding task instructions or general visual impairment difficulties. The recruitment of native-Chinese adults was to avoid any potential confounding effect of the other-race effect on task performance in the face tasks [33]. Younger face rater participants were recruited through the university's undergraduate participant pool, and older face rater participants were recruited from fliers and adverts.

Face rater participants’ age and gender, were recorded at the beginning of the experiment; the Chinese version 20-item Toronto Alexithymia Scale [TAS-20; 34, 35] was also used as a screening evaluation to test all face rater participants for possible emotion-blindness. One young and four older face rater participants scored > 61 on the TAS-20 (indicating for possible difficulties in perceiving emotions) and they were excluded from the study. The Chinese version Mini-Mental State Examination (MMSE) was used as a screening evaluation to test older face rater participants for possible dementia [36, 37]. The MMSE is a commonly used measure to screen for cognitive status. No face rater participants were excluded from the study on the basis of this screening test.

After the exclusion, 34 young (mean age = 23.50 years, SD = 4.41 years, age range, 19–35, female 15) and 31 older (mean age = 65.06 years, SD = 3.50 years, age range, 58–72, female 18) face rater participants were included as raters in the validation test. All face rater participants gave informed consent prior to beginning the experiment and were fully informed about the experimental procedure. The local ethics committee of Department of Psychology, University of Tsinghua approved the study.

#### Procedure

**Fig 1:**
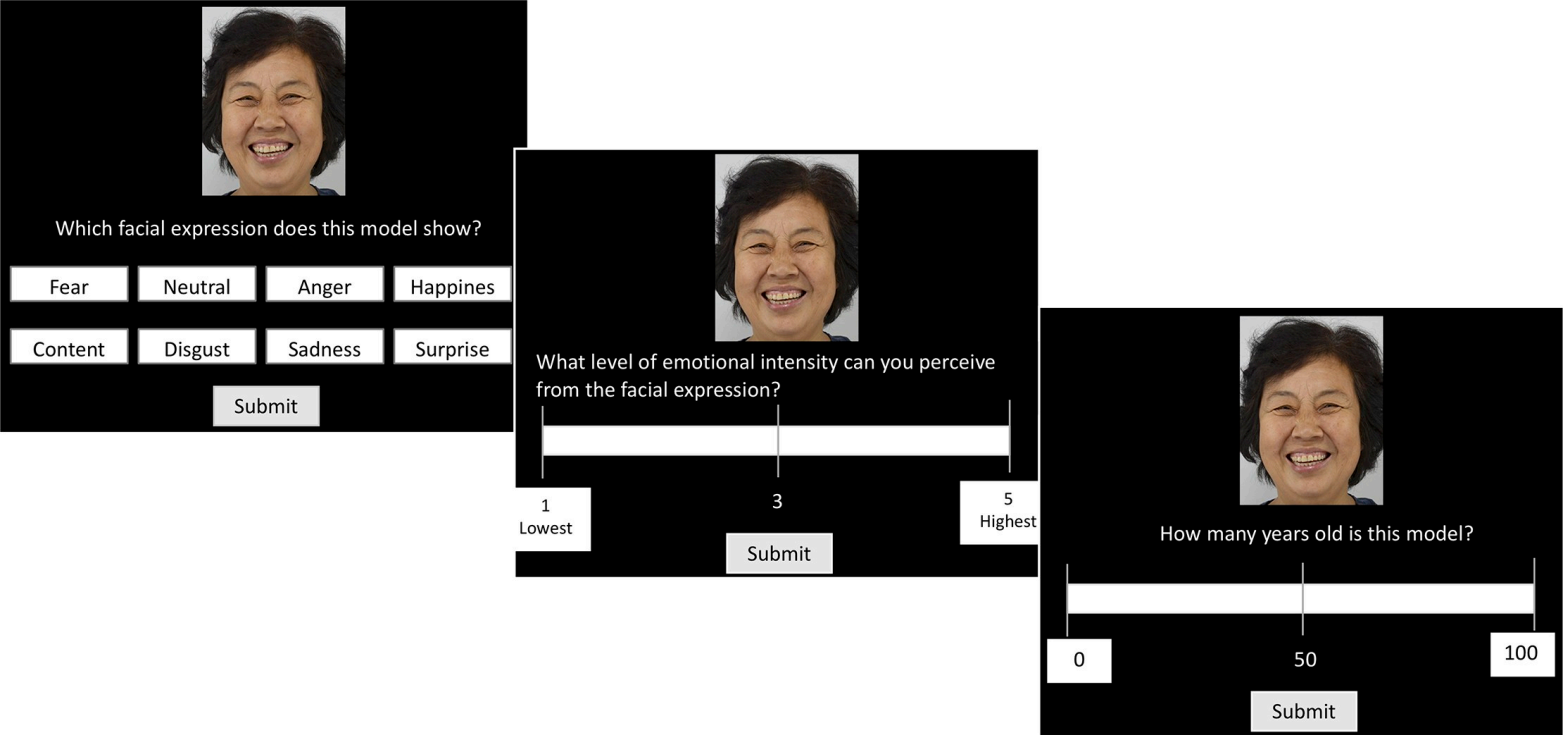
An example of a rating trial. Each selected image was rated by both young and older raters on their 1) perceived facial expression; 2) perceived level of emotional intensity and 3) perceived age of the model. The rating task was self-paced and all raters’ responses were recorded.

Each of the 1128 facial images were rated in terms of perceived facial expression (emotion purity), perceived emotion intensity and perceived age. All photos were randomly assigned to four sessions, each session consisted of 282 images and took around 80 minutes to complete. The experiment was programmed using E-prime 3.0 software (Psychology Software Tools, Inc., Sharpsburg, PA, USA). In each trial (Fig 1), raters were presented with a facial expression image on a laptop computer Lenovo screen (Lenovo Group Ltd., Hong Kong, China), and the task was to 1) decide what facial emotion they perceived by choosing an emotion type label out of eight by clicking the label using a mouse; then 2) decide what level of intensity they perceived from the same image by moving an indicator presented on the screen using a mouse (5 = most intense and 1 = least intense); then 3) they estimated the age of the model presented by moving an indicator presented on the screen using a mouse (from 0 to 100 years). The response keys were aligned in a negative/positive emotion counterbalanced-mixed order in order to avoid negative or positive emotion options clustered together in rows or columns (see Fig 1 for the order of response options). The experiment was self-paced, the screen would not change until a response was received.

The presentation order of the four sessions were randomised for each participant. Each session was further divided into four blocks so raters could rest between blocks. Each rater was required to complete one or more sessions according to their wish. After each session, raters were required to have enough rest interval (minimum of 60 minutes) to proceed to the next session. Raters were not recommended to complete two sessions within the same day. Most raters have completed all four sessions–on average, each image was rated by at least 30 young and 30 older raters.

### Analysis and results

#### Expression identification

**Fig 2:**
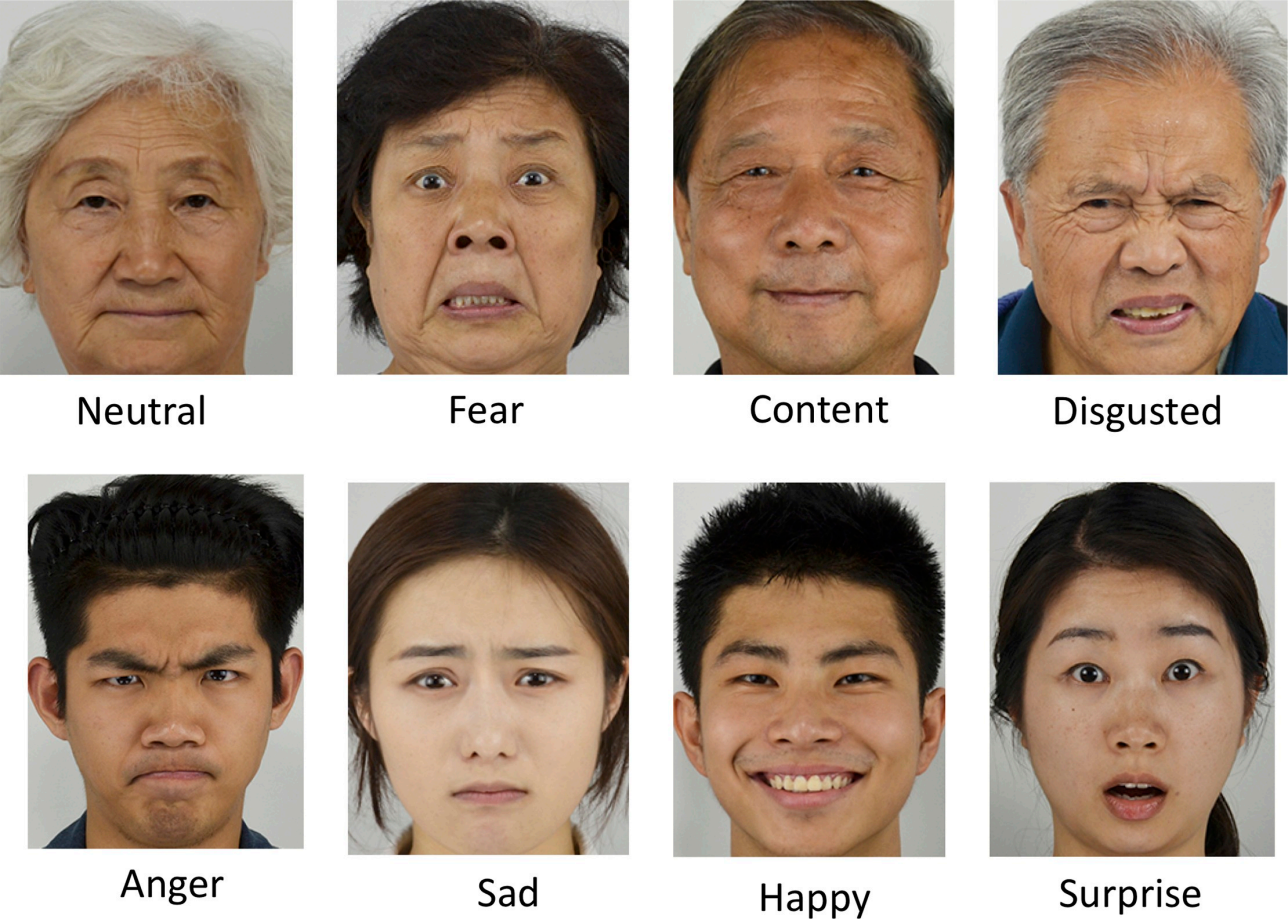
Examples of eight facial expressions by older (upper row) and young (lower row) models.

First, we calculated an percentage of correct identification (based on the mean number of times the model’s facial expressions were correctly identified by the raters) for each face model, which reflects the identifiability level of facial expressions. In the results, these percentages of correct identification ranged from 55.91% to 88.87%. We kept the models with the percentages of correct identification higher than 70% to ensure the facial expression identifiability of the images [similar criteria had been used in constructing other databases, e.g. 23, 32]. The best 47 older (21 male and 26 female) and 63 young (32 male and 31 female) models were retained in the database, and their percentages of correct identification ranged from 70.19% to 88.87%. The remaining analyses were performed using only these 110 models (Table 1). Examples of eight facial expressions by older (upper row) and young (lower row) models are shown in Fig 2.

**Table 1:** Basic demographic and descriptive characteristics of the two model groups (final database set).

	Older (n = 47)	Young (n = 63)
**Gender (male/female)**	21/26	32/31
**Age (years)**	M = 64.60 (range 60–76, SD = 3.49)	M = 23.68 (range 18–33, SD = 4.19)

**Fig 3:**
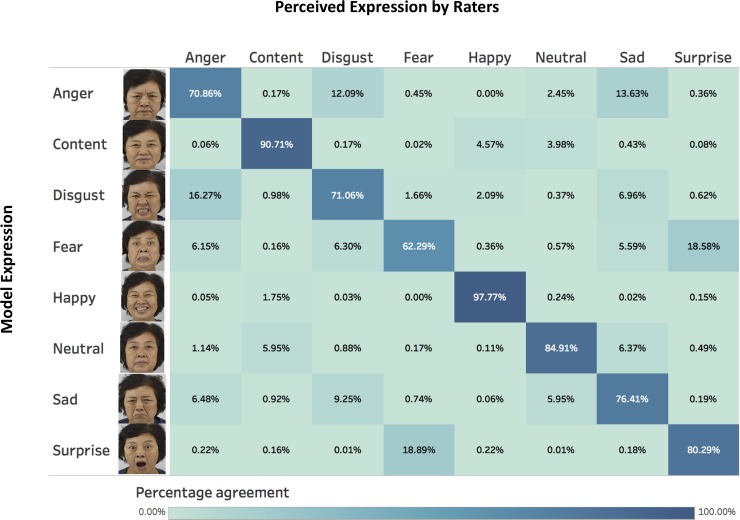
Confusion matrix with example model expressions. Columns represents perceived facial expressions by raters, and rows represents model intended expressions. Diagonal cells represents agreement between raters’ perceived expressions and model intended expressions, with deeper colours representing greater agreement (deepest colour = 100% agreement, lightest colour = 0% agreement).

We computed a confusion matrix to examine the proportion of correct/incorrect identifications of each type facial expression [32, see Fig 3]. The results indicated that Happiness (97.77% correct identification) and Content (90.71% correct identification) were the most accurately identified expressions, followed by Neutral (84.91% correct identification), Surprise (80.29% correct identification), Sad (76.41% correct identification), Disgust (71.06% correct identification), and Anger (70.86% correct identification). Fear (62.29% correct identification) was the least accurately identified expression as it is easily confused with Surprised (18.89% of the Fear images). On average, the expressions were correctly identified in 79.08% (SD = 18.13%) of the images, which is relatively high and are comparable to other facial databases [e.g. 23, 32, 38]. Cohen’s Kappa [39] indicated good agreement between rater-perceived expressions and model’s displayed expressions, Kappa = 0.761, 95% CI (0.757, 0.765), *p* < .001. The mean percentage of correct identification for each model (S1 Table) and each image (S2 Table) are indicated in the Supporting Information.

#### Perceived emotional intensity and perceived age

The overall mean perceived emotional intensity across different facial expressions was 3.844 (SE = .0035). The intraclass correlation coefficient between the raters was .754 (*p* < .001), indicating a high degree of interrater reliability. The mean perceived emotional intensity for each model (S1 Table) and each image (S3 Table) are indicated in the Supporting Information.

The overall mean perceived age of young faces were rated as M = 30.07 years (SE = .36 years, age range 26.4–42.2 years), and older faces as M = 62.64 years (SE = .46 years, age range 56.1–72.0 years) [The mean perceived age for each model is indicated in the S1 Table]. The perceived age results indicate the faces from the Tsinghua database adequately represent the target age groups. Consistent with Ebner et al.’s [23] finding, young faces were rated slightly older than their actual ages. The intraclass correlation coefficient between the raters was .995 (*p* < .001), indicating a high degree of interrater reliability.

## Discussion

In the study, we developed and validated a novel database of experimental stimuli–Tsinghua facial expression database (Tsinghua-FED) that comprises high-quality colour photographs of 110 Chinese young and older female and male faces, each portraying eight basic facial expressions (Neutral, Sadness, Disgust, Fear, Anger, Happiness, Content, and Surprise). In the validation, each image was been rated by at least 60 young and older adults in terms of perceived facial expression (emotion purity), perceived emotion intensity and perceived age. The results have shown that there is good agreement between rater-perceived expressions and model’s expressions. Overall, percentages of correct facial expression identification in the present study were relatively high (M = 79.08%, SD = 18.13%), which is comparable to those in validation studies of other facial databases [23, 32, 38]. This shows that the Tsinghua-FED comprises a valid set of faces displaying the eight different facial expressions. As shown in the results, 97.77% of the Happiness (teeth visible) images and 90.71% of the Content (teeth not visible) faces were correctly identified by raters, they were the most accurately identified expressions. The least accurately identified expression was Fear, only 62.29% of the images were correctly identified, and it was most often confused with Surprise (18.89% of the Fear images). This pattern is similar to other validated databases [31, 32]. In terms of ratings of perceived emotional intensity and perceived age, the intraclass correlation coefficient between raters suggest a high degree of interrater reliability. The overall mean perceived emotional intensity is relatively high and comparable to other face databases [23, 31, 32]. The results of perceived age indicated that the faces from the Tsinghua-FED database adequately represent the target age groups and therefore constitute a valid set of faces in terms of the age of the faces. Taken together, these findings support the validity of the images included in the database, and we also provide information for each model (identification accuracy, perceived emotional intensity, perceived age) which enables future users to choose suitable images to meet their own needs.

As mentioned before, none of existing East-Asian/Chinese face stimuli databases [e.g. JACFEE[27], TFEID [28], CAS-PEA [29], and CMU Multi-PIE database [30]] contain facial images of different age groups (e.g. older adult faces), which is crucial to studying Asian/Chinese adults’ face perceptual abilities across the different adult age groups due to the effect of ‘own-age advantage’. Thus, the current face database goes beyond existing databases by including adults of age 60 and older in addition to traditional young adult individuals. The high-quality coloured images were all validated by native people. In addition, we also provided information for each image (identification accuracy, perceived emotional intensity, perceived age) which enables future users to choose suitable images to meet their own needs.

This database can contribute to examining East Asian or Chinese people’s development of social perception of faces from early to late adulthood. It also provides the possibility to conduct cross-cultural comparison studies to determine the effect of culture on shaping people’s perception of facial expressions and facial identities across the lifespan. For example, Jack et al. [12] report that Eastern Asian (EA) adults have significant deficit in recognising fear and disgust compared to West Caucasians (WC) adults, which might be due to “predetermined social motivations and cultural concepts”. Jack et al.’s [12] conclusion was drawn from investigations that were restricted to West Caucasian and Eastern Asian young adults. It is not known if EA and WC aged observers would show a bigger difference gap since they are more shaped by different cultural rules and social motivations. In future studies, we can further investigate this question in different age groups. The database may also be useful to contribute to the development of related training materials of behavioural intervention that aids facial emotion perceptual skills [e.g. 40].

For both young and older face models, the facial expressions selected for the study were mostly induced by the scenario inductions (please see S5 Table). However, without self-reported data, we cannot entirely confidently say that those facial expressions (induced by scenario and personal event induction methods) are spontaneous or non-posed, since the face models could still pose facial expressions during these induction phases. In addition, based on the data (see S5 Table), we also found a trend that the final images for older face models (23.30%) were more likely to be drawn from controlled facial expression phase than final images for younger models (16.65%), which might reflects older models’ greater discomfort violating display rules during the scenario and personal event induction stages due to older Chinese people’s deeper rooted social norm/expectation and culture-related facial expression display rule [43], therefore images in these conditions were less likely to meet validation criteria for old than young participants, with more controlled expressions required to achieve an equal number of images for old and young participants. Furthermore, it should be noted that the emotion can be accumulated gradually along the induction stages, therefore we cannot guarantee that the emotional facial expression displayed on the final image were fully induced by the corresponding induction phase [especially the 2nd (personal event induction) and 3rd (controlled facial expression induction phase) due to the possible accumulation from previous induction/inductions].

### Limitations and avenues for future research

The pre-selection procedure has been a standardised and essential procedure in most face database creation validation studies across different cultures and age groups [23, 27, 32]. Although it has been established that the facial muscle movements of basic facial expressions are the same across different cultures and ages [4, 5, 11], it is possible that the facial muscle movements of emotional expressions might be subtly shaped by local culture [41, 42]. We will share the whole set of unselected images upon a request sent to the corresponding author and a promise for academic use only, which could permit researchers to conduct the rating procure in naïve local people to investigate whether there is a cultural dialect/accent associated with Chinese facial expressions. This investigation can be more feasible to achieve by asking native participants to rate a sub-set of unselected images and randomly assigning the images across participants.

Using the image inclusion criterion of “70% average correct identification” [23, 27, 32] across all emotion expressions, 47 older and 63 younger models were left in the database. The results showed a larger loss of older face models compared to Ebner et al’s [23] Caucasian face database. This might be due to the use of different types of models: in Ebner et al.’s study, they stated that the all their face models were professional actors/actresses who were recruited from a model agency; in our study, we used ordinary people as face models as we would like to collect the facial expressions from individuals who have not professionally trained to display facial expressions. This might lead to higher exclusion rate of face models during validation. In addition, the higher exclusion rate of older models might be due to Chinese older people’s uneasy feeling in displaying mid- to high- intensity of facial expression in experimental settings due to their deeper rooted social norm/expectation–not showing expressive facial expressions in front of strangers [43]. This might explain that we lost more face models (especially older models) compared to Ebner’s study [23]. However, future research users should be aware that the loss of older face models raises a concern regarding reduced generalizability of this sample of Chinese older faces.

In real life, facial expressions are continuous and dynamic [44]. In future, we intend to include multi-pose, multi-angle and dynamic facial expression stimuli in the database to make it a more comprehensive set of face stimuli database. In addition, the current database can be improved by including more older seniors in both models and raters whose age are older than the older models and raters in the current dataset (ranged from 60–76 years).

## Conclusion

In this study, we constructed and validated a novel facial expression database (Tsinghua-FED) that comprises high-quality colour photographs of 110 Chinese young and older female and male, each portraying eight basic facial expressions (Neutral, Sadness, Disgust, Fear, Anger, Happiness, Content, and Surprise). In the validation, each image was rated by at least 60 young and older adults in terms of perceived facial expression (emotion purity), perceived emotion intensity and perceived age. The validation results support the validity of the images included in the database. Supporting Information has provided information for each image (identification accuracy, perceived emotional intensity, perceived age, induction method) which enables future users to choose suitable images to meet their own needs.

## Supporting information

S1 Table Actual age, perceived age, overall percentage of correct identification, and overall perceived intensity for all valid young and older models included in the final database. (PDF)

S2 Table Identification score for each image (rated by different groups of raters) included in the final database. (PDF)

S3 Table Perceived emotional intensity for each image (rated by different groups of raters) included in the final database. (PDF)

S4 Table Induction method (1-scenario induction; 2-personal event induction; or 3-controlled facial expression) for each image. (PDF)

S5 Table Induction method proportions (%) for each age group by emotion type. (PDF)

S1 Data Scenarios used in scenario induction phase. (PDF)

S2 Data Additional findings (results and discussion) [45–49]. (DOCX)
