# Supplementary material for: Tsinghua facial expression database – A database of facial expressions in Chinese young and older women and men: Development and validation
Source: PLoS One. 2020 Apr 15;15(4):e0231304. doi: 10.1371/journal.pone.0231304 (PMC7159817; doi:10.1371/journal.pone.0231304)
Supplement: S1 Table — (PDF) [file pone.0231304.s001.pdf]

Age Group	Model Gender	Model Code	Actual Age (Years)	Perceived Age (Years)	Overall Percentage Of Correct Identification	Overall Perceived Intensity
**YOUNG**	Male	1	19	26.46	**84.16%**	3.72
**YOUNG**	Male	2	21	28.06	**86.14%**	3.68
**YOUNG**	Female	3	20	25.43	**80.19%**	4.02
**YOUNG**	Female	4	19	26.53	**86.48%**	3.74
**YOUNG**	Female	5	24	28.18	**87.73%**	3.75
**YOUNG**	Female	6	23	26.70	**86.33%**	3.78
**YOUNG**	Male	8	27	30.67	**85.45%**	3.78
**YOUNG**	Male	10	22	28.49	**82.35%**	3.63
**YOUNG**	Male	11	20	24.73	**85.03%**	4.07
**YOUNG**	Female	12	18	25.83	**80.44%**	3.69
**YOUNG**	Female	13	20	26.28	**81.64%**	3.69
**YOUNG**	Female	14	21	27.05	**86.73%**	4.01
**YOUNG**	Male	15	20	29.79	**80.71%**	3.68
**YOUNG**	Male	16	21	30.32	**86.54%**	4.16
**YOUNG**	Female	17	33	29.59	**88.94%**	4.12
**YOUNG**	Female	18	18	26.95	**73.33%**	3.67
**YOUNG**	Female	19	19	27.75	**75.09%**	3.53
**YOUNG**	Male	21	21	27.66	**77.71%**	3.89
**YOUNG**	Female	22	30	30.79	**83.70%**	3.98
**YOUNG**	Female	23	30	32.71	**83.31%**	4.33
**YOUNG**	Male	24	19	27.87	**86.47%**	3.91
**YOUNG**	Female	25	33	33.46	**81.71%**	4.23
**YOUNG**	Female	26	32	31.24	**84.67%**	3.87
**YOUNG**	Male	27	23	28.97	**83.85%**	3.84
**YOUNG**	Male	28	20	27.44	**86.70%**	3.75
**YOUNG**	Male	29	21	30.96	**80.08%**	4.19
**YOUNG**	Male	30	25	29.45	**83.01%**	4.11
**YOUNG**	Female	31	30	36.77	**76.75%**	4.01
**YOUNG**	Female	32	20	30.49	**78.58%**	3.74
**YOUNG**	Male	33	25	31.38	**84.82%**	4.02
**YOUNG**	Male	35	20	30.00	**74.97%**	3.87
**YOUNG**	Male	36	30	43.07	**83.40%**	3.94
**YOUNG**	Female	37	32	39.31	**80.02%**	3.81
**YOUNG**	Female	38	24	28.01	**81.03%**	3.81
**YOUNG**	Female	39	25	31.59	**82.81%**	3.95
**YOUNG**	Female	40	28	31.77	**85.05%**	3.73
**YOUNG**	Male	41	19	27.78	**75.04%**	3.63
**YOUNG**	Female	42	20	26.86	**83.82%**	3.86
**YOUNG**	Male	44	26	32.72	**75.54%**	3.83
**YOUNG**	Male	46	18	32.12	**79.96%**	3.77

**YOUNG**	Male	47	23	29.09	**84.28%**	3.89
**YOUNG**	Female	48	23	28.73	**80.54%**	4.02
**YOUNG**	Male	49	23	33.69	**85.85%**	4.05
**YOUNG**	Female	50	24	33.89	**75.18%**	3.54
**YOUNG**	Female	51	23	32.21	**79.05%**	3.60
**YOUNG**	Female	52	21	28.21	**84.47%**	3.96
**YOUNG**	Male	53	23	28.49	**76.80%**	3.66
**YOUNG**	Male	54	26	31.80	**72.85%**	3.65
**YOUNG**	Male	55	24	31.92	**83.20%**	4.10
**YOUNG**	Male	56	24	29.71	**80.29%**	3.69
**YOUNG**	Male	57	23	31.17	**86.73%**	4.03
**YOUNG**	Male	58	22	28.37	**81.23%**	3.85
**YOUNG**	Female	59	23	31.70	**81.25%**	3.97
**YOUNG**	Male	60	24	31.22	**83.95%**	3.98
**YOUNG**	Female	65	21	29.47	**83.00%**	3.85
**YOUNG**	Female	66	26	30.84	**86.44%**	3.80
**YOUNG**	Male	67	19	28.80	**80.32%**	3.68
**YOUNG**	Male	68	30	38.75	**85.72%**	4.05
**YOUNG**	Female	69	28	32.48	**83.83%**	3.79
**YOUNG**	Female	71	20	31.91	**80.60%**	3.68
**YOUNG**	Female	72	25	32.57	**79.45%**	3.51
**YOUNG**	Male	74	31	51.50	**72.21%**	3.53
**YOUNG**	Male	75	30	39.53	**77.91%**	3.53
**OLD**	Female	4	76	70.60	**77.80%**	3.90
**OLD**	Female	7	65	61.42	**72.30%**	3.84
**OLD**	Male	8	65	62.66	**80.03%**	3.93
**OLD**	Female	9	64	63.67	**72.45%**	3.87
**OLD**	Female	10	60	55.92	**73.62%**	3.59
**OLD**	Male	12	64	56.65	**77.61%**	4.02
**OLD**	Male	15	69	63.29	**84.55%**	4.23
**OLD**	Female	16	64	61.50	**73.11%**	3.73
**OLD**	Male	17	69	61.06	**82.07%**	3.90
**OLD**	Female	19	60	62.96	**87.04%**	4.06
**OLD**	Male	20	65	62.96	**77.13%**	3.80
**OLD**	Male	21	65	62.82	**77.76%**	3.73
**OLD**	Female	22	61	57.28	**70.67%**	3.79
**OLD**	Female	23	66	63.07	**74.00%**	3.79
**OLD**	Female	24	62	60.74	**70.82%**	4.11
**OLD**	Female	26	64	60.49	**78.60%**	3.81
**OLD**	Female	27	65	60.41	**71.35%**	3.58
**OLD**	Female	28	64	64.46	**76.62%**	3.58
**OLD**	Female	29	63	57.95	**85.35%**	4.14
**OLD**	Female	34	65	59.22	**72.63%**	3.63

**OLD**	Male	35	66	63.83	**74.96%**	3.72
**OLD**	Female	38	65	60.57	**76.27%**	3.76
**OLD**	Female	40	61	61.02	**75.07%**	3.76
**OLD**	Female	41	72	70.66	**79.78%**	4.08
**OLD**	Male	42	75	71.03	**74.12%**	4.22
**OLD**	Female	43	62	61.86	**77.37%**	3.73
**OLD**	Female	45	65	61.69	**80.22%**	3.86
**OLD**	Female	47	60	57.69	**82.57%**	3.86
**OLD**	Female	48	65	62.76	**75.30%**	3.97
**OLD**	Female	49	65	60.97	**79.10%**	3.98
**OLD**	Male	50	65	63.97	**79.08%**	3.95
**OLD**	Female	51	60	60.93	**72.90%**	3.91
**OLD**	Female	52	62	58.39	**78.67%**	3.76
**OLD**	Female	53	64	59.39	**78.70%**	3.89
**OLD**	Male	55	64	61.58	**72.67%**	4.13
**OLD**	Female	56	65	61.36	**71.82%**	3.54
**OLD**	Male	58	64	59.54	**77.03%**	4.00
**OLD**	Male	59	65	64.95	**73.92%**	3.72
**OLD**	Male	63	61	61.28	**75.58%**	3.67
**OLD**	Male	64	65	63.17	**80.68%**	3.93
**OLD**	Male	65	65	63.54	**75.10%**	3.60
**OLD**	Male	66	70	71.59	**72.77%**	3.74
**OLD**	Male	67	61	62.02	**79.18%**	3.76
**OLD**	Male	68	60	55.96	**70.05%**	3.69
**OLD**	Male	69	62	60.68	**74.88%**	3.75
**OLD**	Male	70	66	60.37	**74.23%**	3.59
**OLD**	Male	71	65	70.68	**73.61%**	3.99
