# Supplementary material for: Tsinghua facial expression database – A database of facial expressions in Chinese young and older women and men: Development and validation
Source: PLoS One. 2020 Apr 15;15(4):e0231304. doi: 10.1371/journal.pone.0231304 (PMC7159817; doi:10.1371/journal.pone.0231304)
Supplement: S2 Table — (PDF) [file pone.0231304.s002.pdf]

Image Information					Identification Score				
Image Name	Model Age Group	Model Gender	Model Code	Actual Age (Years)	All Raters	Older Female Raters	Young Female Raters	Older Male Raters	Young Male Raters
Y3F-20_happy	Young	Female	3	20	96.49%	93.75%	93.33%	100.00%	100.00%
Y4F-19_happy	Young	Female	4	19	98.28%	100.00%	100.00%	100.00%	92.31%
Y5F-24_happy	Young	Female	5	24	100.00%	100.00%	100.00%	100.00%	100.00%
Y6F-23_happy	Young	Female	6	23	98.28%	100.00%	100.00%	100.00%	92.86%
Y12F-18_happy	Young	Female	12	18	98.33%	100.00%	100.00%	92.31%	100.00%
Y13F-20_happy	Young	Female	13	20	92.98%	87.50%	93.33%	92.31%	100.00%
Y14F-21_happy	Young	Female	14	21	96.67%	93.75%	100.00%	100.00%	93.75%
Y17F-33_happy	Young	Female	17	33	100.00%	100.00%	100.00%	100.00%	100.00%
Y18F-18_happy	Young	Female	18	18	98.25%	93.75%	100.00%	100.00%	100.00%
Y19F-19_happy	Young	Female	19	19	100.00%	100.00%	100.00%	100.00%	100.00%
Y22F-30_happy	Young	Female	22	30	100.00%	100.00%	100.00%	100.00%	100.00%
Y23F-30_happy	Young	Female	23	30	100.00%	100.00%	100.00%	100.00%	100.00%
Y25F-33_happy	Young	Female	25	33	100.00%	100.00%	100.00%	100.00%	100.00%
Y26F-32_happy	Young	Female	26	32	98.28%	100.00%	100.00%	92.31%	100.00%
Y31F-30_happy	Young	Female	31	30	98.28%	100.00%	93.33%	100.00%	100.00%
Y32F-20_happy	Young	Female	32	20	100.00%	100.00%	100.00%	100.00%	100.00%
Y37F-32_happy	Young	Female	37	32	96.49%	100.00%	93.33%	92.31%	100.00%
Y38F-24_happy	Young	Female	38	24	96.49%	93.75%	100.00%	92.31%	100.00%
Y39F-25_happy	Young	Female	39	25	100.00%	100.00%	100.00%	100.00%	100.00%
Y40F-28_happy	Young	Female	40	28	98.28%	100.00%	100.00%	100.00%	92.86%
Y42F-20_happy	Young	Female	42	20	100.00%	100.00%	100.00%	100.00%	100.00%
Y48F-23_happy	Young	Female	48	23	96.55%	93.75%	100.00%	92.86%	100.00%
Y50F-24_happy	Young	Female	50	24	100.00%	100.00%	100.00%	100.00%	100.00%
Y51F-23_happy	Young	Female	51	23	89.47%	75.00%	100.00%	84.62%	100.00%
Y52F-21_happy	Young	Female	52	21	98.28%	93.75%	100.00%	100.00%	100.00%
Y59F-23_happy	Young	Female	59	23	100.00%	100.00%	100.00%	100.00%	100.00%
Y65F-21_happy	Young	Female	65	21	100.00%	100.00%	100.00%	100.00%	100.00%
Y66F-26_happy	Young	Female	66	26	98.25%	93.75%	100.00%	100.00%	100.00%
Y69F-28_happy	Young	Female	69	28	98.28%	93.75%	100.00%	100.00%	100.00%
Y71F-20_happy	Young	Female	71	20	100.00%	100.00%	100.00%	100.00%	100.00%
Y72F-25_happy	Young	Female	72	25	98.28%	93.75%	100.00%	100.00%	100.00%
Y1M-19_happy	Young	Male	1	19	93.10%	87.50%	100.00%	85.71%	100.00%
Y2M-21_happy	Young	Male	2	21	100.00%	100.00%	100.00%	100.00%	100.00%
Y8M-27_happy	Young	Male	8	27	100.00%	100.00%	100.00%	100.00%	100.00%
Y10M-22_happy	Young	Male	10	22	96.67%	93.75%	100.00%	92.31%	100.00%
Y11M-20_happy	Young	Male	11	20	100.00%	100.00%	100.00%	100.00%	100.00%
Y15M-20_happy	Young	Male	15	20	98.28%	100.00%	100.00%	100.00%	92.86%
Y16M-21_happy	Young	Male	16	21	98.28%	93.75%	100.00%	100.00%	100.00%
Y21M-21_happy	Young	Male	21	21	96.67%	100.00%	93.33%	100.00%	93.75%
Y24M-19_happy	Young	Male	24	19	100.00%	100.00%	100.00%	100.00%	100.00%
Y27M-23_happy	Young	Male	27	23	100.00%	100.00%	100.00%	100.00%	100.00%
Y28M-20_happy	Young	Male	28	20	98.33%	93.75%	100.00%	100.00%	100.00%
Y29M-21_happy	Young	Male	29	21	98.28%	100.00%	100.00%	100.00%	92.31%
Y30M-25_happy	Young	Male	30	25	100.00%	100.00%	100.00%	100.00%	100.00%
Y33M-25_happy	Young	Male	33	25	96.49%	93.75%	100.00%	92.31%	100.00%
Y35M-20_happy	Young	Male	35	20	98.28%	100.00%	100.00%	92.86%	100.00%
Y36M-30_happy	Young	Male	36	30	100.00%	100.00%	100.00%	100.00%	100.00%
Y41M-19_happy	Young	Male	41	19	98.25%	93.75%	100.00%	100.00%	100.00%
Y44M-26_happy	Young	Male	44	26	100.00%	100.00%	100.00%	100.00%	100.00%
Y46M-18_happy	Young	Male	46	18	93.10%	87.50%	100.00%	85.71%	100.00%
Y47M-23_happy	Young	Male	47	23	100.00%	100.00%	100.00%	100.00%	100.00%
Y49M-23_happy	Young	Male	49	23	100.00%	100.00%	100.00%	100.00%	100.00%
Y53M-23_happy	Young	Male	53	23	100.00%	100.00%	100.00%	100.00%	100.00%
Y54M-26_happy	Young	Male	54	26	98.28%	100.00%	100.00%	92.86%	100.00%
Y55M-24_happy	Young	Male	55	24	98.25%	100.00%	93.33%	100.00%	100.00%
Y56M-24_happy	Young	Male	56	24	100.00%	100.00%	100.00%	100.00%	100.00%
Y57M-23_happy	Young	Male	57	23	100.00%	100.00%	100.00%	100.00%	100.00%
Y58M-22_happy	Young	Male	58	22	96.49%	100.00%	100.00%	84.62%	100.00%
Y60M-24_happy	Young	Male	60	24	94.74%	93.75%	100.00%	84.62%	100.00%
Y67M-19_happy	Young	Male	67	19	98.33%	100.00%	93.33%	100.00%	100.00%
Y68M-30_happy	Young	Male	68	30	100.00%	100.00%	100.00%	100.00%	100.00%
Y74M-31_happy	Young	Male	74	31	94.74%	100.00%	93.33%	84.62%	100.00%
Y75M-30_happy	Young	Male	75	30	98.25%	93.75%	100.00%	100.00%	100.00%
O4F-76_happy	Old	Female	4	76	100.00%	100.00%	100.00%	100.00%	100.00%
O7F-65_happy	Old	Female	7	65	93.10%	93.75%	100.00%	78.57%	100.00%
O9F-64_happy	Old	Female	9	64	96.49%	93.75%	93.33%	100.00%	100.00%
O10F-60_happy	Old	Female	10	60	98.28%	93.75%	100.00%	100.00%	100.00%
O16F-64_happy	Old	Female	16	64	98.28%	93.75%	100.00%	100.00%	100.00%
O19F-60_happy	Old	Female	19	60	100.00%	100.00%	100.00%	100.00%	100.00%
O22F-61_happy	Old	Female	22	61	100.00%	100.00%	100.00%	100.00%	100.00%
O23F-66_happy	Old	Female	23	66	100.00%	100.00%	100.00%	100.00%	81.25%
O24F-62_happy	Old	Female	24	62	96.55%	100.00%	100.00%	85.71%	100.00%
O26F-64_happy	Old	Female	26	64	98.28%	100.00%	100.00%	92.86%	100.00%
O27F-65_happy	Old	Female	27	65	100.00%	100.00%	100.00%	100.00%	100.00%
O28F-64_happy	Old	Female	28	64	91.38%	93.75%	100.00%	78.57%	92.31%
O29F-63_happy	Old	Female	29	63	100.00%	100.00%	100.00%	100.00%	100.00%
O34F-65_happy	Old	Female	34	65	98.28%	100.00%	100.00%	100.00%	92.31%
O38F-65_happy	Old	Female	38	65	98.33%	93.75%	100.00%	100.00%	100.00%
O40F-61_happy	Old	Female	40	61	96.55%	100.00%	100.00%	85.71%	100.00%
O41F-72_happy	Old	Female	41	72	100.00%	100.00%	100.00%	100.00%	100.00%
O43F-62_happy	Old	Female	43	62	100.00%	100.00%	100.00%	100.00%	100.00%
O45F-65_happy	Old	Female	45	65	91.23%	87.50%	100.00%	84.62%	92.31%
O47F-60_happy	Old	Female	47	60	98.33%	100.00%	100.00%	92.31%	100.00%
O48F-65_happy	Old	Female	48	65	100.00%	100.00%	100.00%	100.00%	100.00%
O49F-65_happy	Old	Female	49	65	91.23%	87.50%	100.00%	84.62%	92.31%
O51F-60_happy	Old	Female	51	60	94.83%	93.75%	93.33%	92.86%	100.00%
O52F-62_happy	Old	Female	52	62	94.83%	93.75%	93.33%	92.86%	100.00%
O53F-64_happy	Old	Female	53	64	100.00%	100.00%	100.00%	100.00%	100.00%
O56F-65_happy	Old	Female	56	65	96.55%	100.00%	100.00%	85.71%	100.00%
O8M-65_happy	Old	Male	8	65	98.28%	100.00%	100.00%	92.86%	100.00%
O12M-64_happy	Old	Male	12	64	93.33%	100.00%	73.33%	100.00%	100.00%
O15M-69_happy	Old	Male	15	69	92.98%	87.50%	100.00%	92.31%	92.31%
O17M-69_happy	Old	Male	17	69	100.00%	100.00%	100.00%	100.00%	100.00%
O20M-65_happy	Old	Male	20	65	98.25%	93.75%	100.00%	100.00%	100.00%
O21M-65_happy	Old	Male	21	65	100.00%	100.00%	100.00%	100.00%	100.00%
O35M-66_happy	Old	Male	35	66	98.28%	93.75%	100.00%	100.00%	100.00%
O42M-75_happy	Old	Male	42	75	96.49%	100.00%	93.33%	100.00%	92.31%
O50M-65_happy	Old	Male	50	65	96.55%	93.75%	100.00%	92.86%	100.00%
O55M-64_happy	Old	Male	55	64	91.23%	93.75%	93.33%	84.62%	92.31%
O58M-64_happy	Old	Male	58	64	100.00%	100.00%	100.00%	100.00%	100.00%
O59M-65_happy	Old	Male	59	65	98.33%	100.00%	100.00%	92.31%	100.00%
O63M-61_happy	Old	Male	63	61	96.67%	100.00%	100.00%	92.31%	93.75%
O64M-65_happy	Old	Male	64	65	100.00%	100.00%	100.00%	100.00%	100.00%
O65M-65_happy	Old	Male	65	65	94.74%	93.75%	100.00%	100.00%	84.62%
O66M-70_happy	Old	Male	66	70	96.67%	93.75%	100.00%	100.00%	93.75%
O67M-61_happy	Old	Male	67	61	96.49%	93.75%	100.00%	100.00%	92.31%
O68M-60_happy	Old	Male	68	60	77.19%	68.75%	86.67%	69.23%	84.62%
O69M-62_happy	Old	Male	69	62	100.00%	100.00%	100.00%	100.00%	100.00%
O70M-66_happy	Old	Male	70	66	100.00%	100.00%	100.00%	100.00%	100.00%
O71M-65_happy	Old	Male	71	65	100.00%	100.00%	100.00%	100.00%	100.00%

Image Information					Identification Score					
Image Name	Model Age Group	Model Gender	Model Code	Actual Age (Years)	All Raters	Older Female Raters	Young Female Raters	Older Male Raters	Young Male Raters
Y3F-20_content	Young	Female	3	20	92.98%	87.50%	100.00%	92.31%	92.31%
Y4F-19_content	Young	Female	4	19	89.66%	100.00%	100.00%	84.62%	71.43%
Y5F-24_content	Young	Female	5	24	98.28%	93.75%	100.00%	100.00%	100.00%
Y6F-23_content	Young	Female	6	23	91.67%	93.75%	86.67%	92.31%	93.75%
Y12F-18_content	Young	Female	12	18	96.67%	100.00%	93.33%	100.00%	93.75%
Y13F-20_content	Young	Female	13	20	100.00%	100.00%	100.00%	100.00%	100.00%
Y14F-21_content	Young	Female	14	21	95.00%	93.75%	100.00%	92.31%	93.75%
Y17F-33_content	Young	Female	17	33	94.83%	93.75%	100.00%	92.31%	92.86%
Y18F-18_content	Young	Female	18	18	100.00%	100.00%	100.00%	100.00%	100.00%
Y19F-19_content	Young	Female	19	19	91.23%	81.25%	100.00%	92.31%	92.31%
Y22F-30_content	Young	Female	22	30	93.10%	93.75%	100.00%	85.71%	92.31%
Y23F-30_content	Young	Female	23	30	80.70%	75.00%	93.33%	76.92%	76.92%
Y25F-33_content	Young	Female	25	33	84.21%	81.25%	86.67%	76.92%	92.31%
Y26F-32_content	Young	Female	26	32	94.74%	93.75%	100.00%	92.31%	92.31%
Y31F-30_content	Young	Female	31	30	96.55%	100.00%	100.00%	92.31%	92.86%
Y32F-20_content	Young	Female	32	20	90.00%	93.75%	93.33%	76.92%	93.75%
Y37F-32_content	Young	Female	37	32	94.83%	87.50%	100.00%	100.00%	92.86%
Y38F-24_content	Young	Female	38	24	81.03%	68.75%	100.00%	71.43%	84.62%
Y39F-25_content	Young	Female	39	25	98.28%	100.00%	100.00%	92.86%	100.00%
Y40F-28_content	Young	Female	40	28	98.33%	100.00%	93.33%	100.00%	100.00%
Y42F-20_content	Young	Female	42	20	94.74%	81.25%	100.00%	100.00%	100.00%
Y48F-23_content	Young	Female	48	23	93.33%	100.00%	100.00%	92.31%	81.25%
Y50F-24_content	Young	Female	50	24	85.96%	81.25%	93.33%	84.62%	84.62%
Y51F-23_content	Young	Female	51	23	94.74%	87.50%	100.00%	100.00%	92.31%
Y52F-21_content	Young	Female	52	21	84.21%	68.75%	93.33%	76.92%	100.00%
Y59F-23_content	Young	Female	59	23	96.67%	100.00%	100.00%	92.31%	93.75%
Y65F-21_content	Young	Female	65	21	95.00%	93.75%	93.33%	100.00%	93.75%
Y66F-26_content	Young	Female	66	26	95.00%	93.75%	100.00%	92.31%	93.75%
Y69F-28_content	Young	Female	69	28	96.67%	100.00%	100.00%	100.00%	87.50%
Y71F-20_content	Young	Female	71	20	96.55%	93.75%	100.00%	100.00%	92.86%
Y72F-25_content	Young	Female	72	25	98.25%	100.00%	100.00%	92.31%	100.00%
Y1M-19_content	Young	Male	1	19	76.67%	87.50%	73.33%	69.23%	75.00%
Y2M-21_content	Young	Male	2	21	94.83%	93.75%	100.00%	92.31%	92.86%
Y8M-27_content	Young	Male	8	27	98.28%	100.00%	100.00%	92.86%	100.00%
Y10M-22_content	Young	Male	10	22	94.83%	87.50%	100.00%	92.31%	100.00%
Y11M-20_content	Young	Male	11	20	82.76%	87.50%	93.33%	71.43%	76.92%
Y15M-20_content	Young	Male	15	20	100.00%	100.00%	100.00%	100.00%	100.00%
Y16M-21_content	Young	Male	16	21	95.00%	93.75%	100.00%	100.00%	87.50%
Y21M-21_content	Young	Male	21	21	89.66%	93.75%	86.67%	85.71%	92.31%
Y24M-19_content	Young	Male	24	19	91.38%	81.25%	100.00%	100.00%	85.71%
Y27M-23_content	Young	Male	27	23	88.33%	62.50%	100.00%	100.00%	93.75%
Y28M-20_content	Young	Male	28	20	83.33%	81.25%	100.00%	69.23%	81.25%
Y29M-21_content	Young	Male	29	21	91.23%	93.75%	100.00%	92.31%	76.92%
Y30M-25_content	Young	Male	30	25	91.23%	81.25%	100.00%	92.31%	92.31%
Y33M-25_content	Young	Male	33	25	98.28%	100.00%	100.00%	92.31%	100.00%
Y35M-20_content	Young	Male	35	20	98.28%	100.00%	100.00%	100.00%	92.86%
Y36M-30_content	Young	Male	36	30	98.28%	100.00%	93.33%	100.00%	100.00%
Y41M-19_content	Young	Male	41	19	65.00%	56.25%	93.33%	53.85%	56.25%
Y44M-26_content	Young	Male	44	26	94.83%	93.75%	100.00%	92.86%	92.31%
Y46M-18_content	Young	Male	46	18	98.25%	93.75%	100.00%	100.00%	100.00%
Y47M-23_content	Young	Male	47	23	92.98%	81.25%	100.00%	92.31%	100.00%
Y49M-23_content	Young	Male	49	23	91.38%	81.25%	100.00%	92.31%	92.86%
Y53M-23_content	Young	Male	53	23	94.83%	100.00%	100.00%	76.92%	100.00%
Y54M-26_content	Young	Male	54	26	98.28%	100.00%	93.33%	100.00%	100.00%
Y55M-24_content	Young	Male	55	24	98.33%	100.00%	100.00%	100.00%	93.75%
Y56M-24_content	Young	Male	56	24	96.55%	87.50%	100.00%	100.00%	100.00%
Y57M-23_content	Young	Male	57	23	100.00%	100.00%	100.00%	100.00%	100.00%
Y58M-22_content	Young	Male	58	22	96.55%	93.75%	100.00%	92.31%	100.00%
Y60M-24_content	Young	Male	60	24	98.33%	100.00%	100.00%	100.00%	93.75%
Y67M-19_content	Young	Male	67	19	91.67%	93.75%	86.67%	92.31%	93.75%
Y68M-30_content	Young	Male	68	30	61.40%	37.50%	73.33%	53.85%	84.62%
Y74M-31_content	Young	Male	74	31	85.96%	87.50%	100.00%	84.62%	69.23%
Y75M-30_content	Young	Male	75	30	87.93%	81.25%	100.00%	85.71%	84.62%
O4F-76_content	Old	Female	4	76	87.93%	93.75%	86.67%	84.62%	85.71%
O7F-65_content	Old	Female	7	65	68.97%	56.25%	60.00%	64.29%	100.00%
O9F-64_content	Old	Female	9	64	96.67%	100.00%	100.00%	84.62%	100.00%
O10F-60_content	Old	Female	10	60	85.96%	81.25%	86.67%	92.31%	84.62%
O16F-64_content	Old	Female	16	64	81.03%	87.50%	86.67%	76.92%	71.43%
O19F-60_content	Old	Female	19	60	96.55%	93.75%	100.00%	100.00%	92.86%
O22F-61_content	Old	Female	22	61	96.55%	87.50%	100.00%	100.00%	100.00%
O23F-66_content	Old	Female	23	66	60.00%	75.00%	60.00%	76.92%	81.25%
O24F-62_content	Old	Female	24	62	70.00%	43.75%	86.67%	76.92%	75.00%
O26F-64_content	Old	Female	26	64	55.17%	50.00%	80.00%	50.00%	38.46%
O27F-65_content	Old	Female	27	65	94.83%	93.75%	93.33%	100.00%	92.86%
O28F-64_content	Old	Female	28	64	96.67%	100.00%	100.00%	100.00%	87.50%
O29F-63_content	Old	Female	29	63	94.83%	81.25%	100.00%	100.00%	100.00%
O34F-65_content	Old	Female	34	65	98.28%	93.75%	100.00%	100.00%	100.00%
O38F-65_content	Old	Female	38	65	93.10%	81.25%	100.00%	92.86%	100.00%
O40F-61_content	Old	Female	40	61	61.40%	56.25%	86.67%	61.54%	38.46%
O41F-72_content	Old	Female	41	72	94.83%	87.50%	100.00%	92.31%	100.00%
O43F-62_content	Old	Female	43	62	72.41%	81.25%	86.67%	57.14%	61.54%
O45F-65_content	Old	Female	45	65	96.67%	100.00%	93.33%	100.00%	93.75%
O47F-60_content	Old	Female	47	60	96.67%	100.00%	93.33%	92.31%	100.00%
O48F-65_content	Old	Female	48	65	96.67%	100.00%	100.00%	92.31%	93.75%
O49F-65_content	Old	Female	49	65	82.76%	75.00%	86.67%	84.62%	85.71%
O51F-60_content	Old	Female	51	60	87.72%	87.50%	93.33%	84.62%	84.62%
O52F-62_content	Old	Female	52	62	94.83%	87.50%	100.00%	92.86%	100.00%
O53F-64_content	Old	Female	53	64	94.74%	100.00%	100.00%	84.62%	92.31%
O56F-65_content	Old	Female	56	65	83.33%	87.50%	100.00%	69.23%	75.00%
O8M-65_content	Old	Male	8	65	96.67%	100.00%	100.00%	92.31%	93.75%
O12M-64_content	Old	Male	12	64	89.47%	87.50%	100.00%	69.23%	100.00%
O15M-69_content	Old	Male	15	69	100.00%	100.00%	100.00%	100.00%	100.00%
O17M-69_content	Old	Male	17	69	92.98%	87.50%	100.00%	92.31%	92.31%
O20M-65_content	Old	Male	20	65	100.00%	100.00%	100.00%	100.00%	100.00%
O21M-65_content	Old	Male	21	65	94.74%	81.25%	100.00%	100.00%	100.00%
O35M-66_content	Old	Male	35	66	93.10%	93.75%	100.00%	84.62%	92.86%
O42M-75_content	Old	Male	42	75	94.83%	93.75%	100.00%	85.71%	100.00%
O50M-65_content	Old	Male	50	65	73.68%	81.25%	80.00%	69.23%	61.54%
O55M-64_content	Old	Male	55	64	95.00%	100.00%	93.33%	100.00%	87.50%
O58M-64_content	Old	Male	58	64	92.98%	100.00%	100.00%	69.23%	100.00%
O59M-65_content	Old	Male	59	65	94.83%	93.75%	100.00%	92.86%	92.31%
O63M-61_content	Old	Male	63	61	91.23%	93.75%	86.67%	100.00%	84.62%
O64M-65_content	Old	Male	64	65	92.98%	93.75%	86.67%	92.31%	100.00%
O65M-65_content	Old	Male	65	65	94.74%	100.00%	93.33%	84.62%	100.00%
O66M-70_content	Old	Male	66	70	100.00%	100.00%	100.00%	100.00%	100.00%
O67M-61_content	Old	Male	67	61	87.93%	93.75%	93.33%	78.57%	84.62%
O68M-60_content	Old	Male	68	60	87.93%	87.50%	93.33%	92.31%	78.57%
O69M-62_content	Old	Male	69	62	89.66%	87.50%	93.33%	85.71%	92.31%
O70M-66_content	Old	Male	70	66	77.59%	75.00%	80.00%	71.43%	84.62%
O71M-65_content	Old	Male	71	65	84.21%	81.25%	93.33%	69.23%	92.31%

Image Information					Identification Score				
Image Name	Model Age Group	Model Gender	Model Code	Actual Age (Years)	All Raters	Older Female Raters	Young Female Raters	Older Male Raters	Young Male Raters
Y3F-20_Neutral	Young	Female	3	20	86.21%	81.25%	93.33%	76.92%	92.86%
Y4F-19_Neutral	Young	Female	4	19	88.33%	87.50%	86.67%	84.62%	93.75%
Y5F-24_Neutral	Young	Female	5	24	95.00%	93.75%	100.00%	92.31%	93.75%
Y6F-23_Neutral	Young	Female	6	23	93.10%	87.50%	100.00%	85.71%	100.00%
Y12F-18_Neutral	Young	Female	12	18	94.74%	81.25%	100.00%	100.00%	100.00%
Y13F-20_Neutral	Young	Female	13	20	70.00%	56.25%	80.00%	61.54%	81.25%
Y14F-21_Neutral	Young	Female	14	21	94.83%	93.75%	100.00%	84.62%	100.00%
Y17F-33_Neutral	Young	Female	17	33	98.33%	93.75%	100.00%	100.00%	100.00%
Y18F-18_Neutral	Young	Female	18	18	96.49%	100.00%	93.33%	100.00%	92.31%
Y19F-19_Neutral	Young	Female	19	19	93.10%	87.50%	100.00%	84.62%	100.00%
Y22F-30_Neutral	Young	Female	22	30	91.38%	93.75%	86.67%	85.71%	100.00%
Y23F-30_Neutral	Young	Female	23	30	92.98%	81.25%	100.00%	92.31%	100.00%
Y25F-33_Neutral	Young	Female	25	33	93.33%	87.50%	93.33%	92.31%	100.00%
Y26F-32_Neutral	Young	Female	26	32	72.41%	62.50%	93.33%	42.86%	92.31%
Y31F-30_Neutral	Young	Female	31	30	81.03%	87.50%	80.00%	84.62%	71.43%
Y32F-20_Neutral	Young	Female	32	20	90.00%	81.25%	93.33%	84.62%	100.00%
Y37F-32_Neutral	Young	Female	37	32	82.76%	93.75%	80.00%	71.43%	84.62%
Y38F-24_Neutral	Young	Female	38	24	94.83%	93.75%	100.00%	85.71%	100.00%
Y39F-25_Neutral	Young	Female	39	25	93.33%	87.50%	100.00%	92.31%	93.75%
Y40F-28_Neutral	Young	Female	40	28	89.66%	87.50%	93.33%	85.71%	92.31%
Y42F-20_Neutral	Young	Female	42	20	89.66%	81.25%	93.33%	92.86%	92.31%
Y48F-23_Neutral	Young	Female	48	23	81.67%	68.75%	100.00%	69.23%	87.50%
Y50F-24_Neutral	Young	Female	50	24	91.38%	81.25%	100.00%	84.62%	100.00%
Y51F-23_Neutral	Young	Female	51	23	91.67%	93.75%	93.33%	76.92%	100.00%
Y52F-21_Neutral	Young	Female	52	21	89.47%	87.50%	93.33%	76.92%	100.00%
Y59F-23_Neutral	Young	Female	59	23	71.67%	75.00%	66.67%	61.54%	81.25%
Y65F-21_Neutral	Young	Female	65	21	82.76%	87.50%	86.67%	61.54%	92.86%
Y66F-26_Neutral	Young	Female	66	26	98.33%	100.00%	100.00%	92.31%	100.00%
Y69F-28_Neutral	Young	Female	69	28	90.00%	81.25%	100.00%	92.31%	87.50%
Y71F-20_Neutral	Young	Female	71	20	96.49%	93.75%	100.00%	92.31%	100.00%
Y72F-25_Neutral	Young	Female	72	25	96.67%	93.75%	100.00%	100.00%	93.75%
Y1M-19_Neutral	Young	Male	1	19	96.55%	93.75%	100.00%	92.31%	100.00%
Y2M-21_Neutral	Young	Male	2	21	88.33%	87.50%	80.00%	92.31%	93.75%
Y8M-27_Neutral	Young	Male	8	27	98.28%	100.00%	100.00%	92.31%	100.00%
Y10M-22_Neutral	Young	Male	10	22	91.38%	93.75%	93.33%	76.92%	100.00%
Y11M-20_Neutral	Young	Male	11	20	96.67%	93.75%	100.00%	92.31%	100.00%
Y15M-20_Neutral	Young	Male	15	20	95.00%	81.25%	100.00%	100.00%	100.00%
Y16M-21_Neutral	Young	Male	16	21	86.21%	87.50%	93.33%	78.57%	84.62%
Y21M-21_Neutral	Young	Male	21	21	95.00%	93.75%	100.00%	84.62%	100.00%
Y24M-19_Neutral	Young	Male	24	19	90.00%	81.25%	93.33%	84.62%	100.00%
Y27M-23_Neutral	Young	Male	27	23	79.31%	87.50%	93.33%	42.86%	92.31%
Y28M-20_Neutral	Young	Male	28	20	98.28%	100.00%	100.00%	92.31%	100.00%
Y29M-21_Neutral	Young	Male	29	21	92.98%	87.50%	93.33%	92.31%	100.00%
Y30M-25_Neutral	Young	Male	30	25	80.00%	62.50%	100.00%	53.85%	100.00%
Y33M-25_Neutral	Young	Male	33	25	91.38%	87.50%	93.33%	85.71%	100.00%
Y35M-20_Neutral	Young	Male	35	20	43.10%	43.75%	60.00%	14.29%	53.85%
Y36M-30_Neutral	Young	Male	36	30	87.93%	81.25%	93.33%	76.92%	100.00%
Y41M-19_Neutral	Young	Male	41	19	95.00%	100.00%	93.33%	84.62%	100.00%
Y44M-26_Neutral	Young	Male	44	26	81.67%	75.00%	93.33%	69.23%	87.50%
Y46M-18_Neutral	Young	Male	46	18	89.47%	81.25%	93.33%	84.62%	100.00%
Y47M-23_Neutral	Young	Male	47	23	94.83%	87.50%	100.00%	92.31%	100.00%
Y49M-23_Neutral	Young	Male	49	23	94.83%	93.75%	93.33%	92.31%	100.00%
Y53M-23_Neutral	Young	Male	53	23	79.31%	81.25%	86.67%	71.43%	76.92%
Y54M-26_Neutral	Young	Male	54	26	91.38%	87.50%	100.00%	76.92%	100.00%
Y55M-24_Neutral	Young	Male	55	24	87.93%	93.75%	80.00%	92.86%	84.62%
Y56M-24_Neutral	Young	Male	56	24	94.83%	93.75%	100.00%	84.62%	100.00%
Y57M-23_Neutral	Young	Male	57	23	96.55%	100.00%	100.00%	84.62%	100.00%
Y58M-22_Neutral	Young	Male	58	22	87.72%	81.25%	93.33%	92.31%	84.62%
Y60M-24_Neutral	Young	Male	60	24	84.48%	87.50%	93.33%	64.29%	92.31%
Y67M-19_Neutral	Young	Male	67	19	53.45%	43.75%	46.67%	50.00%	76.92%
Y68M-30_Neutral	Young	Male	68	30	92.98%	87.50%	100.00%	84.62%	100.00%
Y74M-31_Neutral	Young	Male	74	31	92.98%	93.75%	86.67%	92.31%	100.00%
Y75M-30_Neutral	Young	Male	75	30	87.72%	81.25%	86.67%	84.62%	100.00%
O4F-76_Neutral	Old	Female	4	76	98.33%	100.00%	100.00%	100.00%	93.75%
O7F-65_Neutral	Old	Female	7	65	77.59%	75.00%	80.00%	71.43%	84.62%
O9F-64_Neutral	Old	Female	9	64	72.41%	68.75%	73.33%	57.14%	92.31%
O10F-60_Neutral	Old	Female	10	60	84.48%	87.50%	73.33%	78.57%	100.00%
O16F-64_Neutral	Old	Female	16	64	89.66%	93.75%	93.33%	71.43%	100.00%
O19F-60_Neutral	Old	Female	19	60	72.41%	75.00%	60.00%	71.43%	84.62%
O22F-61_Neutral	Old	Female	22	61	15.00%	18.75%	6.67%	30.77%	6.25%
O23F-66_Neutral	Old	Female	23	66	72.41%	75.00%	73.33%	64.29%	81.25%
O24F-62_Neutral	Old	Female	24	62	61.40%	56.25%	60.00%	46.15%	84.62%
O26F-64_Neutral	Old	Female	26	64	80.70%	81.25%	80.00%	69.23%	92.31%
O27F-65_Neutral	Old	Female	27	65	79.31%	81.25%	80.00%	64.29%	92.31%
O28F-64_Neutral	Old	Female	28	64	71.93%	81.25%	66.67%	84.62%	53.85%
O29F-63_Neutral	Old	Female	29	63	84.48%	81.25%	86.67%	78.57%	92.31%
O34F-65_Neutral	Old	Female	34	65	94.74%	93.75%	93.33%	100.00%	92.31%
O38F-65_Neutral	Old	Female	38	65	87.72%	81.25%	100.00%	76.92%	92.31%
O40F-61_Neutral	Old	Female	40	61	82.46%	87.50%	73.33%	92.31%	76.92%
O41F-72_Neutral	Old	Female	41	72	86.21%	81.25%	93.33%	71.43%	100.00%
O43F-62_Neutral	Old	Female	43	62	94.83%	93.75%	100.00%	85.71%	100.00%
O45F-65_Neutral	Old	Female	45	65	96.55%	100.00%	100.00%	92.31%	92.86%
O47F-60_Neutral	Old	Female	47	60	86.21%	93.75%	93.33%	57.14%	100.00%
O48F-65_Neutral	Old	Female	48	65	85.96%	93.75%	73.33%	76.92%	100.00%
O49F-65_Neutral	Old	Female	49	65	48.33%	37.50%	60.00%	23.08%	68.75%
O51F-60_Neutral	Old	Female	51	60	82.76%	81.25%	86.67%	71.43%	92.31%
O52F-62_Neutral	Old	Female	52	62	77.59%	62.50%	86.67%	69.23%	92.86%
O53F-64_Neutral	Old	Female	53	64	85.00%	75.00%	86.67%	76.92%	100.00%
O56F-65_Neutral	Old	Female	56	65	86.67%	75.00%	80.00%	100.00%	93.75%
O8M-65_Neutral	Old	Male	8	65	91.67%	81.25%	93.33%	92.31%	100.00%
O12M-64_Neutral	Old	Male	12	64	92.98%	87.50%	100.00%	84.62%	100.00%
O15M-69_Neutral	Old	Male	15	69	95.00%	93.75%	93.33%	92.31%	100.00%
O17M-69_Neutral	Old	Male	17	69	85.00%	87.50%	80.00%	76.92%	93.75%
O20M-65_Neutral	Old	Male	20	65	78.95%	75.00%	80.00%	61.54%	100.00%
O21M-65_Neutral	Old	Male	21	65	96.55%	93.75%	100.00%	92.31%	100.00%
O35M-66_Neutral	Old	Male	35	66	82.46%	81.25%	66.67%	100.00%	84.62%
O42M-75_Neutral	Old	Male	42	75	68.33%	68.75%	80.00%	46.15%	75.00%
O50M-65_Neutral	Old	Male	50	65	94.83%	93.75%	100.00%	85.71%	100.00%
O55M-64_Neutral	Old	Male	55	64	68.97%	68.75%	53.33%	64.29%	92.31%
O58M-64_Neutral	Old	Male	58	64	73.33%	43.75%	86.67%	76.92%	87.50%
O59M-65_Neutral	Old	Male	59	65	89.47%	87.50%	93.33%	76.92%	100.00%
O63M-61_Neutral	Old	Male	63	61	44.83%	50.00%	46.67%	23.08%	57.14%
O64M-65_Neutral	Old	Male	64	65	86.67%	81.25%	80.00%	92.31%	93.75%
O65M-65_Neutral	Old	Male	65	65	75.44%	87.50%	60.00%	84.62%	69.23%
O66M-70_Neutral	Old	Male	66	70	89.66%	100.00%	80.00%	84.62%	92.86%
O67M-61_Neutral	Old	Male	67	61	94.83%	100.00%	93.33%	84.62%	100.00%
O68M-60_Neutral	Old	Male	68	60	68.97%	75.00%	66.67%	71.43%	61.54%
O69M-62_Neutral	Old	Male	69	62	93.10%	93.75%	100.00%	84.62%	92.86%
O70M-66_Neutral	Old	Male	70	66	87.93%	93.75%	73.33%	92.86%	92.31%
O71M-65_Neutral	Old	Male	71	65	85.96%	81.25%	80.00%	84.62%	100.00%

Image Information					Identification Score				
Image Name	Model Age Group	Model Gender	Model Code	Actual Age (Years)	All Raters	Older Female Raters	Young Female Raters	Older Male Raters	Young Male Raters
Y3F-20_sad	Young	Female	3	20	98.33%	100.00%	100.00%	92.31%	100.00%
Y4F-19_sad	Young	Female	4	19	93.33%	100.00%	100.00%	69.23%	100.00%
Y5F-24_sad	Young	Female	5	24	91.23%	87.50%	100.00%	76.92%	100.00%
Y6F-23_sad	Young	Female	6	23	98.25%	100.00%	100.00%	92.31%	100.00%
Y12F-18_sad	Young	Female	12	18	63.79%	56.25%	86.67%	53.85%	57.14%
Y13F-20_sad	Young	Female	13	20	94.83%	100.00%	100.00%	78.57%	100.00%
Y14F-21_sad	Young	Female	14	21	81.67%	68.75%	93.33%	76.92%	87.50%
Y17F-33_sad	Young	Female	17	33	98.33%	93.75%	100.00%	100.00%	100.00%
Y18F-18_sad	Young	Female	18	18	82.76%	75.00%	100.00%	64.29%	92.31%
Y19F-19_sad	Young	Female	19	19	77.19%	68.75%	86.67%	61.54%	92.31%
Y22F-30_sad	Young	Female	22	30	91.38%	87.50%	93.33%	85.71%	100.00%
Y23F-30_sad	Young	Female	23	30	77.19%	81.25%	93.33%	53.85%	76.92%
Y25F-33_sad	Young	Female	25	33	91.23%	87.50%	100.00%	92.31%	84.62%
Y26F-32_sad	Young	Female	26	32	81.03%	68.75%	80.00%	100.00%	78.57%
Y31F-30_sad	Young	Female	31	30	55.17%	34.38%	63.33%	61.54%	64.29%
Y32F-20_sad	Young	Female	32	20	85.96%	75.00%	100.00%	69.23%	100.00%
Y37F-32_sad	Young	Female	37	32	68.33%	56.25%	86.67%	53.85%	75.00%
Y38F-24_sad	Young	Female	38	24	74.14%	68.75%	86.67%	64.29%	76.92%
Y39F-25_sad	Young	Female	39	25	87.93%	68.75%	100.00%	84.62%	100.00%
Y40F-28_sad	Young	Female	40	28	91.38%	93.75%	100.00%	78.57%	92.31%
Y42F-20_sad	Young	Female	42	20	93.10%	93.75%	100.00%	76.92%	100.00%
Y48F-23_sad	Young	Female	48	23	70.00%	68.75%	80.00%	38.46%	87.50%
Y50F-24_sad	Young	Female	50	24	21.05%	25.00%	13.33%	15.38%	30.77%
Y51F-23_sad	Young	Female	51	23	84.21%	87.50%	80.00%	84.62%	84.62%
Y52F-21_sad	Young	Female	52	21	86.21%	87.50%	100.00%	69.23%	85.71%
Y59F-23_sad	Young	Female	59	23	96.49%	100.00%	100.00%	84.62%	100.00%
Y65F-21_sad	Young	Female	65	21	94.83%	93.75%	100.00%	84.62%	100.00%
Y66F-26_sad	Young	Female	66	26	54.39%	62.50%	40.00%	38.46%	76.92%
Y69F-28_sad	Young	Female	69	28	86.21%	81.25%	93.33%	78.57%	92.31%
Y71F-20_sad	Young	Female	71	20	48.28%	43.75%	73.33%	30.77%	42.86%
Y72F-25_sad	Young	Female	72	25	63.33%	68.75%	60.00%	53.85%	68.75%
Y1M-19_sad	Young	Male	1	19	88.33%	81.25%	100.00%	69.23%	100.00%
Y2M-21_sad	Young	Male	2	21	88.33%	87.50%	93.33%	69.23%	100.00%
Y8M-27_sad	Young	Male	8	27	96.55%	93.75%	100.00%	92.86%	100.00%
Y10M-22_sad	Young	Male	10	22	70.18%	93.75%	66.67%	46.15%	69.23%
Y11M-20_sad	Young	Male	11	20	98.33%	100.00%	100.00%	100.00%	93.75%
Y15M-20_sad	Young	Male	15	20	46.55%	50.00%	46.67%	15.38%	71.43%
Y16M-21_sad	Young	Male	16	21	70.00%	56.25%	93.33%	38.46%	87.50%
Y21M-21_sad	Young	Male	21	21	63.33%	31.25%	93.33%	46.15%	81.25%
Y24M-19_sad	Young	Male	24	19	87.93%	87.50%	100.00%	78.57%	84.62%
Y27M-23_sad	Young	Male	27	23	84.21%	81.25%	93.33%	69.23%	92.31%
Y28M-20_sad	Young	Male	28	20	92.98%	93.75%	100.00%	84.62%	92.31%
Y29M-21_sad	Young	Male	29	21	87.72%	93.75%	93.33%	69.23%	92.31%
Y30M-25_sad	Young	Male	30	25	95.00%	93.75%	100.00%	100.00%	87.50%
Y33M-25_sad	Young	Male	33	25	92.98%	87.50%	100.00%	84.62%	100.00%
Y35M-20_sad	Young	Male	35	20	78.33%	68.75%	73.33%	84.62%	87.50%
Y36M-30_sad	Young	Male	36	30	65.52%	62.50%	33.33%	85.71%	84.62%
Y41M-19_sad	Young	Male	41	19	87.72%	93.75%	80.00%	84.62%	92.31%
Y44M-26_sad	Young	Male	44	26	96.55%	100.00%	100.00%	85.71%	100.00%
Y46M-18_sad	Young	Male	46	18	91.23%	87.50%	100.00%	76.92%	100.00%
Y47M-23_sad	Young	Male	47	23	95.00%	93.75%	100.00%	84.62%	100.00%
Y49M-23_sad	Young	Male	49	23	98.25%	100.00%	100.00%	92.31%	100.00%
Y53M-23_sad	Young	Male	53	23	38.60%	25.00%	60.00%	23.08%	46.15%
Y54M-26_sad	Young	Male	54	26	29.31%	37.50%	40.00%	7.14%	30.77%
Y55M-24_sad	Young	Male	55	24	73.33%	68.75%	66.67%	69.23%	87.50%
Y56M-24_sad	Young	Male	56	24	87.93%	81.25%	93.33%	78.57%	100.00%
Y57M-23_sad	Young	Male	57	23	93.33%	87.50%	100.00%	84.62%	100.00%
Y58M-22_sad	Young	Male	58	22	76.92%	78.13%	93.33%	46.15%	86.21%
Y60M-24_sad	Young	Male	60	24	94.83%	100.00%	93.33%	92.86%	92.31%
Y67M-19_sad	Young	Male	67	19	75.00%	87.50%	80.00%	69.23%	62.50%
Y68M-30_sad	Young	Male	68	30	98.33%	100.00%	100.00%	92.31%	100.00%
Y74M-31_sad	Young	Male	74	31	61.40%	62.50%	80.00%	30.77%	69.23%
Y75M-30_sad	Young	Male	75	30	51.67%	43.75%	46.67%	38.46%	75.00%
O4F-76_sad	Old	Female	4	76	77.59%	75.00%	93.33%	61.54%	78.57%
O7F-65_sad	Old	Female	7	65	94.83%	87.50%	100.00%	92.31%	100.00%
O9F-64_sad	Old	Female	9	64	45.61%	62.50%	40.00%	38.46%	38.46%
O10F-60_sad	Old	Female	10	60	73.33%	62.50%	93.33%	76.92%	62.50%
O16F-64_sad	Old	Female	16	64	64.91%	81.25%	33.33%	69.23%	76.92%
O19F-60_sad	Old	Female	19	60	100.00%	100.00%	100.00%	100.00%	100.00%
O22F-61_sad	Old	Female	22	61	41.38%	50.00%	40.00%	38.46%	35.71%
O23F-66_sad	Old	Female	23	66	78.95%	93.75%	73.33%	76.92%	81.25%
O24F-62_sad	Old	Female	24	62	70.00%	75.00%	86.67%	53.85%	62.50%
O26F-64_sad	Old	Female	26	64	98.28%	100.00%	100.00%	92.31%	100.00%
O27F-65_sad	Old	Female	27	65	29.31%	18.75%	13.33%	46.15%	42.86%
O28F-64_sad	Old	Female	28	64	90.00%	87.50%	100.00%	76.92%	93.75%
O29F-63_sad	Old	Female	29	63	86.21%	81.25%	93.33%	69.23%	100.00%
O34F-65_sad	Old	Female	34	65	60.34%	50.00%	66.67%	50.00%	76.92%
O38F-65_sad	Old	Female	38	65	70.00%	56.25%	86.67%	53.85%	81.25%
O40F-61_sad	Old	Female	40	61	63.16%	75.00%	66.67%	61.54%	46.15%
O41F-72_sad	Old	Female	41	72	94.83%	93.75%	100.00%	85.71%	100.00%
O43F-62_sad	Old	Female	43	62	32.76%	37.50%	33.33%	38.46%	21.43%
O45F-65_sad	Old	Female	45	65	92.98%	100.00%	93.33%	92.31%	84.62%
O47F-60_sad	Old	Female	47	60	81.03%	81.25%	80.00%	76.92%	85.71%
O48F-65_sad	Old	Female	48	65	35.09%	50.00%	20.00%	38.46%	30.77%
O49F-65_sad	Old	Female	49	65	96.55%	93.75%	93.33%	100.00%	100.00%
O51F-60_sad	Old	Female	51	60	93.10%	93.75%	93.33%	92.31%	92.86%
O52F-62_sad	Old	Female	52	62	93.10%	100.00%	93.33%	92.86%	84.62%
O53F-64_sad	Old	Female	53	64	84.48%	75.00%	80.00%	100.00%	84.62%
O56F-65_sad	Old	Female	56	65	58.62%	75.00%	53.33%	46.15%	57.14%
O8M-65_sad	Old	Male	8	65	48.33%	43.75%	60.00%	38.46%	50.00%
O12M-64_sad	Old	Male	12	64	91.38%	93.75%	100.00%	69.23%	100.00%
O15M-69_sad	Old	Male	15	69	87.93%	81.25%	100.00%	78.57%	92.31%
O17M-69_sad	Old	Male	17	69	89.66%	93.75%	93.33%	69.23%	100.00%
O20M-65_sad	Old	Male	20	65	93.33%	81.25%	100.00%	92.31%	100.00%
O21M-65_sad	Old	Male	21	65	68.97%	75.00%	60.00%	71.43%	69.23%
O35M-66_sad	Old	Male	35	66	84.48%	87.50%	93.33%	71.43%	84.62%
O42M-75_sad	Old	Male	42	75	55.00%	75.00%	53.33%	38.46%	50.00%
O50M-65_sad	Old	Male	50	65	87.93%	81.25%	93.33%	85.71%	92.31%
O55M-64_sad	Old	Male	55	64	98.28%	93.75%	100.00%	100.00%	100.00%
O58M-64_sad	Old	Male	58	64	53.45%	43.75%	60.00%	46.15%	64.29%
O59M-65_sad	Old	Male	59	65	56.14%	75.00%	40.00%	38.46%	69.23%
O63M-61_sad	Old	Male	63	61	90.00%	100.00%	86.67%	92.31%	81.25%
O64M-65_sad	Old	Male	64	65	79.31%	81.25%	86.67%	57.14%	92.31%
O65M-65_sad	Old	Male	65	65	75.86%	75.00%	66.67%	76.92%	85.71%
O66M-70_sad	Old	Male	66	70	17.24%	31.25%	6.67%	23.08%	7.14%
O67M-61_sad	Old	Male	67	61	91.38%	87.50%	93.33%	92.31%	92.86%
O68M-60_sad	Old	Male	68	60	94.83%	100.00%	93.33%	84.62%	100.00%
O69M-62_sad	Old	Male	69	62	33.33%	18.75%	53.33%	15.38%	46.15%
O70M-66_sad	Old	Male	70	66	74.14%	75.00%	86.67%	46.15%	85.71%
O71M-65_sad	Old	Male	71	65	58.33%	62.50%	53.33%	53.85%	62.50%

Image Information					Identification Score				
Image Name	Model Age Group	Model Gender	Model Code	Actual Age (Years)	All Raters	Older Female Raters	Young Female Raters	Older Male Raters	Young Male Raters
Y3F-20_anger	Young	Female	3	20	24.14%	6.25%	33.33%	14.29%	46.15%
Y4F-19_anger	Young	Female	4	19	84.21%	81.25%	93.33%	69.23%	92.31%
Y5F-24_anger	Young	Female	5	24	84.48%	81.25%	86.67%	69.23%	100.00%
Y6F-23_anger	Young	Female	6	23	79.31%	81.25%	86.67%	76.92%	71.43%
Y12F-18_anger	Young	Female	12	18	89.66%	93.75%	93.33%	85.71%	84.62%
Y13F-20_anger	Young	Female	13	20	91.38%	81.25%	100.00%	92.31%	92.86%
Y14F-21_anger	Young	Female	14	21	88.33%	75.00%	93.33%	84.62%	100.00%
Y17F-33_anger	Young	Female	17	33	86.67%	75.00%	100.00%	84.62%	87.50%
Y18F-18_anger	Young	Female	18	18	29.31%	31.25%	20.00%	28.57%	38.46%
Y19F-19_anger	Young	Female	19	19	63.16%	68.75%	73.33%	46.15%	61.54%
Y22F-30_anger	Young	Female	22	30	70.69%	68.75%	93.33%	42.86%	76.92%
Y23F-30_anger	Young	Female	23	30	96.67%	93.75%	100.00%	100.00%	93.75%
Y25F-33_anger	Young	Female	25	33	63.16%	56.25%	60.00%	53.85%	84.62%
Y26F-32_anger	Young	Female	26	32	85.00%	81.25%	86.67%	76.92%	93.75%
Y31F-30_anger	Young	Female	31	30	85.96%	81.25%	86.67%	84.62%	92.31%
Y32F-20_anger	Young	Female	32	20	18.97%	31.25%	13.33%	14.29%	15.38%
Y37F-32_anger	Young	Female	37	32	86.21%	93.75%	86.67%	78.57%	84.62%
Y38F-24_anger	Young	Female	38	24	79.31%	87.50%	80.00%	71.43%	76.92%
Y39F-25_anger	Young	Female	39	25	56.90%	50.00%	73.33%	50.00%	53.85%
Y40F-28_anger	Young	Female	40	28	73.91%	68.75%	83.33%	66.67%	76.92%
Y42F-20_anger	Young	Female	42	20	46.55%	37.50%	33.33%	57.14%	61.54%
Y48F-23_anger	Young	Female	48	23	83.33%	68.75%	100.00%	61.54%	100.00%
Y50F-24_anger	Young	Female	50	24	67.24%	43.75%	80.00%	53.85%	92.86%
Y51F-23_anger	Young	Female	51	23	63.33%	43.75%	93.33%	38.46%	75.00%
Y52F-21_anger	Young	Female	52	21	94.83%	87.50%	93.33%	100.00%	100.00%
Y59F-23_anger	Young	Female	59	23	39.66%	18.75%	46.67%	42.86%	53.85%
Y65F-21_anger	Young	Female	65	21	86.21%	81.25%	93.33%	78.57%	92.31%
Y66F-26_anger	Young	Female	66	26	85.00%	75.00%	93.33%	84.62%	87.50%
Y69F-28_anger	Young	Female	69	28	75.00%	68.75%	73.33%	76.92%	81.25%
Y71F-20_anger	Young	Female	71	20	96.55%	93.75%	100.00%	92.86%	100.00%
Y72F-25_anger	Young	Female	72	25	73.33%	75.00%	80.00%	61.54%	75.00%
Y1M-19_anger	Young	Male	1	19	98.25%	100.00%	100.00%	92.31%	100.00%
Y2M-21_anger	Young	Male	2	21	96.49%	100.00%	100.00%	84.62%	100.00%
Y8M-27_anger	Young	Male	8	27	46.67%	31.25%	53.33%	53.85%	50.00%
Y10M-22_anger	Young	Male	10	22	76.67%	75.00%	73.33%	76.92%	81.25%
Y11M-20_anger	Young	Male	11	20	77.19%	56.25%	86.67%	69.23%	100.00%
Y15M-20_anger	Young	Male	15	20	92.98%	93.75%	93.33%	84.62%	100.00%
Y16M-21_anger	Young	Male	16	21	91.67%	87.50%	100.00%	84.62%	93.75%
Y21M-21_anger	Young	Male	21	21	85.96%	75.00%	93.33%	76.92%	100.00%
Y24M-19_anger	Young	Male	24	19	89.66%	75.00%	100.00%	84.62%	100.00%
Y27M-23_anger	Young	Male	27	23	82.76%	87.50%	80.00%	85.71%	76.92%
Y28M-20_anger	Young	Male	28	20	87.93%	93.75%	93.33%	61.54%	100.00%
Y29M-21_anger	Young	Male	29	21	48.33%	25.00%	53.33%	46.15%	68.75%
Y30M-25_anger	Young	Male	30	25	93.10%	87.50%	93.33%	100.00%	92.31%
Y33M-25_anger	Young	Male	33	25	56.14%	50.00%	70.00%	50.00%	53.85%
Y35M-20_anger	Young	Male	35	20	74.14%	68.75%	86.67%	57.14%	84.62%
Y36M-30_anger	Young	Male	36	30	91.38%	90.63%	93.33%	88.46%	92.86%
Y41M-19_anger	Young	Male	41	19	46.55%	37.50%	80.00%	14.29%	53.85%
Y44M-26_anger	Young	Male	44	26	42.11%	25.00%	66.67%	30.77%	46.15%
Y46M-18_anger	Young	Male	46	18	95.00%	93.75%	100.00%	84.62%	100.00%
Y47M-23_anger	Young	Male	47	23	51.72%	56.25%	66.67%	35.71%	46.15%
Y49M-23_anger	Young	Male	49	23	89.66%	100.00%	93.33%	69.23%	92.86%
Y53M-23_anger	Young	Male	53	23	91.38%	93.75%	86.67%	92.31%	92.86%
Y54M-26_anger	Young	Male	54	26	84.48%	87.50%	80.00%	76.92%	92.86%
Y55M-24_anger	Young	Male	55	24	92.98%	75.00%	100.00%	100.00%	100.00%
Y56M-24_anger	Young	Male	56	24	55.17%	50.00%	60.00%	46.15%	64.29%
Y57M-23_anger	Young	Male	57	23	85.96%	87.50%	93.33%	84.62%	76.92%
Y58M-22_anger	Young	Male	58	22	75.86%	62.50%	86.67%	64.29%	92.31%
Y60M-24_anger	Young	Male	60	24	65.52%	75.00%	80.00%	35.71%	69.23%
Y67M-19_anger	Young	Male	67	19	86.21%	87.50%	93.33%	69.23%	92.86%
Y68M-30_anger	Young	Male	68	30	85.00%	81.25%	93.33%	76.92%	87.50%
Y74M-31_anger	Young	Male	74	31	58.62%	59.38%	60.00%	60.71%	53.85%
Y75M-30_anger	Young	Male	75	30	83.62%	90.63%	90.00%	74.07%	77.78%
O4F-76_anger	Old	Female	4	76	61.40%	62.50%	60.00%	46.15%	76.92%
O7F-65_anger	Old	Female	7	65	11.67%	25.00%	0.00%	23.08%	0.00%
O9F-64_anger	Old	Female	9	64	54.39%	56.25%	73.33%	30.77%	53.85%
O10F-60_anger	Old	Female	10	60	64.91%	81.25%	66.67%	30.77%	76.92%
O16F-64_anger	Old	Female	16	64	21.67%	31.25%	26.67%	7.69%	18.75%
O19F-60_anger	Old	Female	19	60	96.67%	87.50%	100.00%	100.00%	100.00%
O22F-61_anger	Old	Female	22	61	89.66%	93.75%	80.00%	85.71%	100.00%
O23F-66_anger	Old	Female	23	66	61.67%	75.00%	60.00%	61.54%	50.00%
O24F-62_anger	Old	Female	24	62	60.00%	68.75%	60.00%	84.62%	31.25%
O26F-64_anger	Old	Female	26	64	95.00%	100.00%	100.00%	100.00%	81.25%
O27F-65_anger	Old	Female	27	65	58.33%	50.00%	86.67%	30.77%	62.50%
O28F-64_anger	Old	Female	28	64	79.31%	87.50%	73.33%	69.23%	85.71%
O29F-63_anger	Old	Female	29	63	83.33%	75.00%	100.00%	61.54%	93.75%
O34F-65_anger	Old	Female	34	65	61.40%	56.25%	66.67%	38.46%	84.62%
O38F-65_anger	Old	Female	38	65	70.18%	68.75%	80.00%	53.85%	76.92%
O40F-61_anger	Old	Female	40	61	86.67%	87.50%	93.33%	76.92%	87.50%
O41F-72_anger	Old	Female	41	72	30.00%	12.50%	66.67%	15.38%	25.00%
O43F-62_anger	Old	Female	43	62	87.93%	87.50%	93.33%	69.23%	100.00%
O45F-65_anger	Old	Female	45	65	33.33%	25.00%	40.00%	15.38%	53.85%
O47F-60_anger	Old	Female	47	60	84.21%	75.00%	86.67%	76.92%	100.00%
O48F-65_anger	Old	Female	48	65	92.98%	93.75%	93.33%	92.31%	92.31%
O49F-65_anger	Old	Female	49	65	86.67%	81.25%	100.00%	69.23%	93.75%
O51F-60_anger	Old	Female	51	60	31.67%	50.00%	33.33%	23.08%	18.75%
O52F-62_anger	Old	Female	52	62	43.10%	50.00%	33.33%	50.00%	38.46%
O53F-64_anger	Old	Female	53	64	71.67%	62.50%	93.33%	61.54%	68.75%
O56F-65_anger	Old	Female	56	65	81.03%	81.25%	86.67%	53.85%	100.00%
O8M-65_anger	Old	Male	8	65	76.72%	84.38%	76.67%	65.38%	78.57%
O12M-64_anger	Old	Male	12	64	44.83%	18.75%	66.67%	42.86%	53.85%
O15M-69_anger	Old	Male	15	69	61.67%	62.50%	60.00%	76.92%	50.00%
O17M-69_anger	Old	Male	17	69	75.44%	87.50%	66.67%	76.92%	69.23%
O20M-65_anger	Old	Male	20	65	81.67%	75.00%	86.67%	69.23%	93.75%
O21M-65_anger	Old	Male	21	65	60.00%	75.00%	73.33%	61.54%	31.25%
O35M-66_anger	Old	Male	35	66	44.83%	37.50%	73.33%	14.29%	53.85%
O42M-75_anger	Old	Male	42	75	72.41%	75.00%	86.67%	50.00%	76.92%
O50M-65_anger	Old	Male	50	65	80.70%	87.50%	86.67%	76.92%	69.23%
O55M-64_anger	Old	Male	55	64	60.00%	68.75%	46.67%	84.62%	43.75%
O58M-64_anger	Old	Male	58	64	88.89%	81.25%	96.67%	80.77%	96.55%
O59M-65_anger	Old	Male	59	65	68.97%	68.75%	86.67%	57.69%	60.71%
O63M-61_anger	Old	Male	63	61	68.97%	68.75%	86.67%	38.46%	78.57%
O64M-65_anger	Old	Male	64	65	66.67%	56.25%	86.67%	69.23%	56.25%
O65M-65_anger	Old	Male	65	65	75.86%	75.00%	80.00%	69.23%	78.57%
O66M-70_anger	Old	Male	66	70	56.14%	31.25%	86.67%	61.54%	46.15%
O67M-61_anger	Old	Male	67	61	79.31%	62.50%	80.00%	100.00%	76.92%
O68M-60_anger	Old	Male	68	60	38.60%	62.50%	13.33%	53.85%	23.08%
O69M-62_anger	Old	Male	69	62	44.83%	56.25%	46.67%	42.86%	30.77%
O70M-66_anger	Old	Male	70	66	49.14%	71.88%	30.00%	55.56%	37.04%
O71M-65_anger	Old	Male	71	65	63.79%	75.00%	46.67%	69.23%	64.29%

Image Information					Identification Score				
Image Name	Model Age Group	Model Gender	Model Code	Actual Age (Years)	All Raters	Older Female Raters	Young Female Raters	Older Male Raters	Young Male Raters
Y3F-20_fear	Young	Female	3	20	65.52%	62.50%	80.00%	50.00%	69.23%
Y4F-19_fear	Young	Female	4	19	77.59%	62.50%	93.33%	64.29%	92.31%
Y5F-24_fear	Young	Female	5	24	61.40%	68.75%	80.00%	23.08%	69.23%
Y6F-23_fear	Young	Female	6	23	63.33%	68.75%	80.00%	46.15%	56.25%
Y12F-18_fear	Young	Female	12	18	22.41%	6.25%	60.00%	0.00%	21.43%
Y13F-20_fear	Young	Female	13	20	30.00%	25.00%	53.33%	15.38%	25.00%
Y14F-21_fear	Young	Female	14	21	50.88%	37.50%	66.67%	23.08%	76.92%
Y17F-33_fear	Young	Female	17	33	81.67%	81.25%	86.67%	69.23%	87.50%
Y18F-18_fear	Young	Female	18	18	41.38%	56.25%	33.33%	42.86%	30.77%
Y19F-19_fear	Young	Female	19	19	39.66%	25.00%	66.67%	15.38%	50.00%
Y22F-30_fear	Young	Female	22	30	63.16%	50.00%	86.67%	46.15%	69.23%
Y23F-30_fear	Young	Female	23	30	75.86%	68.75%	93.33%	46.15%	92.86%
Y25F-33_fear	Young	Female	25	33	72.41%	62.50%	93.33%	35.71%	100.00%
Y26F-32_fear	Young	Female	26	32	75.44%	75.00%	86.67%	53.85%	84.62%
Y31F-30_fear	Young	Female	31	30	63.16%	50.00%	73.33%	53.85%	76.92%
Y32F-20_fear	Young	Female	32	20	70.00%	56.25%	86.67%	53.85%	81.25%
Y37F-32_fear	Young	Female	37	32	56.90%	43.75%	73.33%	42.86%	69.23%
Y38F-24_fear	Young	Female	38	24	63.79%	50.00%	73.33%	38.46%	92.86%
Y39F-25_fear	Young	Female	39	25	83.33%	68.75%	93.33%	84.62%	87.50%
Y40F-28_fear	Young	Female	40	28	79.31%	75.00%	73.33%	84.62%	85.71%
Y42F-20_fear	Young	Female	42	20	72.41%	75.00%	80.00%	57.14%	76.92%
Y48F-23_fear	Young	Female	48	23	65.52%	62.50%	66.67%	61.54%	71.43%
Y50F-24_fear	Young	Female	50	24	57.89%	56.25%	73.33%	23.08%	76.92%
Y51F-23_fear	Young	Female	51	23	35.09%	37.50%	40.00%	30.77%	30.77%
Y52F-21_fear	Young	Female	52	21	75.86%	75.00%	73.33%	69.23%	85.71%
Y59F-23_fear	Young	Female	59	23	76.67%	56.25%	93.33%	61.54%	93.75%
Y65F-21_fear	Young	Female	65	21	58.62%	50.00%	86.67%	42.86%	53.85%
Y66F-26_fear	Young	Female	66	26	77.59%	75.00%	93.33%	38.46%	100.00%
Y69F-28_fear	Young	Female	69	28	56.14%	18.75%	93.33%	30.77%	84.62%
Y71F-20_fear	Young	Female	71	20	57.89%	68.75%	53.33%	30.77%	76.92%
Y72F-25_fear	Young	Female	72	25	49.12%	50.00%	66.67%	30.77%	46.15%
Y1M-19_fear	Young	Male	1	19	50.00%	31.25%	73.33%	38.46%	56.25%
Y2M-21_fear	Young	Male	2	21	53.45%	43.75%	80.00%	23.08%	64.29%
Y8M-27_fear	Young	Male	8	27	79.31%	75.00%	73.33%	69.23%	100.00%
Y10M-22_fear	Young	Male	10	22	56.67%	50.00%	60.00%	30.77%	81.25%
Y11M-20_fear	Young	Male	11	20	56.52%	53.13%	73.33%	34.62%	62.96%
Y15M-20_fear	Young	Male	15	20	59.65%	43.75%	66.67%	46.15%	84.62%
Y16M-21_fear	Young	Male	16	21	73.73%	65.63%	90.00%	55.56%	82.76%
Y21M-21_fear	Young	Male	21	21	58.33%	50.00%	66.67%	38.46%	75.00%
Y24M-19_fear	Young	Male	24	19	79.31%	68.75%	93.33%	53.85%	100.00%
Y28M-20_fear	Young	Male	28	20	77.59%	71.88%	90.00%	61.54%	85.71%
Y29M-21_fear	Young	Male	29	21	77.59%	87.50%	93.33%	42.86%	84.62%
Y30M-25_fear	Young	Male	30	25	81.03%	68.75%	93.33%	71.43%	92.31%
Y33M-25_fear	Young	Male	33	25	82.76%	75.00%	93.33%	61.54%	100.00%
Y35M-20_fear	Young	Male	35	20	73.33%	68.75%	93.33%	53.85%	75.00%
Y36M-30_fear	Young	Male	36	30	55.17%	43.75%	60.00%	38.46%	78.57%
Y41M-19_fear	Young	Male	41	19	56.90%	43.75%	93.33%	23.08%	64.29%
Y44M-26_fear	Young	Male	44	26	55.17%	31.25%	86.67%	30.77%	71.43%
Y46M-18_fear	Young	Male	46	18	50.00%	25.00%	73.33%	35.71%	69.23%
Y47M-23_fear	Young	Male	47	23	70.00%	56.25%	93.33%	38.46%	87.50%
Y49M-23_fear	Young	Male	49	23	71.93%	75.00%	80.00%	46.15%	84.62%
Y53M-23_fear	Young	Male	53	23	40.00%	37.50%	60.00%	7.69%	50.00%
Y54M-26_fear	Young	Male	54	26	48.33%	37.50%	73.33%	30.77%	50.00%
Y55M-24_fear	Young	Male	55	24	71.67%	68.75%	80.00%	61.54%	75.00%
Y56M-24_fear	Young	Male	56	24	54.39%	56.25%	80.00%	23.08%	53.85%
Y57M-23_fear	Young	Male	57	23	73.68%	62.50%	93.33%	61.54%	76.92%
Y58M-22_fear	Young	Male	58	22	72.41%	75.00%	86.67%	50.00%	76.92%
Y60M-24_fear	Young	Male	60	24	56.14%	56.25%	80.00%	23.08%	61.54%
Y67M-19_fear	Young	Male	67	19	51.67%	37.50%	73.33%	30.77%	62.50%
Y68M-30_fear	Young	Male	68	30	73.33%	68.75%	86.67%	61.54%	75.00%
Y74M-31_fear	Young	Male	74	31	35.09%	43.75%	40.00%	38.46%	15.38%
Y75M-30_fear	Young	Male	75	30	72.41%	68.75%	86.67%	64.29%	69.23%
O4F-76_fear	Old	Female	4	76	47.83%	21.88%	73.33%	22.22%	76.92%
O7F-65_fear	Old	Female	7	65	71.93%	56.25%	80.00%	69.23%	84.62%
O9F-64_fear	Old	Female	9	64	70.69%	62.50%	73.33%	53.85%	92.86%
O10F-60_fear	Old	Female	10	60	34.48%	43.75%	33.33%	0.00%	57.14%
O16F-64_fear	Old	Female	16	64	81.03%	81.25%	93.33%	61.54%	85.71%
O19F-60_fear	Old	Female	19	60	80.70%	75.00%	86.67%	61.54%	100.00%
O22F-61_fear	Old	Female	22	61	58.62%	50.00%	80.00%	46.15%	57.14%
O23F-66_fear	Old	Female	23	66	75.86%	81.25%	66.67%	53.85%	100.00%
O24F-62_fear	Old	Female	24	62	82.76%	75.00%	86.67%	85.71%	84.62%
O26F-64_fear	Old	Female	26	64	72.41%	62.50%	86.67%	57.14%	81.25%
O27F-65_fear	Old	Female	27	65	25.86%	12.50%	46.67%	14.29%	30.77%
O28F-64_fear	Old	Female	28	64	54.39%	56.25%	66.67%	15.38%	76.92%
O29F-63_fear	Old	Female	29	63	87.93%	75.00%	100.00%	92.31%	85.71%
O34F-65_fear	Old	Female	34	65	18.97%	18.75%	13.33%	7.69%	35.71%
O38F-65_fear	Old	Female	38	65	32.76%	12.50%	53.33%	23.08%	42.86%
O40F-61_fear	Old	Female	40	61	79.31%	68.75%	86.67%	78.57%	84.62%
O41F-72_fear	Old	Female	41	72	72.41%	75.00%	86.67%	50.00%	76.92%
O43F-62_fear	Old	Female	43	62	81.03%	87.50%	80.00%	76.92%	78.57%
O45F-65_fear	Old	Female	45	65	56.90%	37.50%	66.67%	50.00%	76.92%
O47F-60_fear	Old	Female	47	60	60.34%	68.75%	73.33%	30.77%	64.29%
O48F-65_fear	Old	Female	48	65	55.00%	50.00%	60.00%	30.77%	75.00%
O49F-65_fear	Old	Female	49	65	86.67%	75.00%	100.00%	84.62%	87.50%
O51F-60_fear	Old	Female	51	60	60.34%	56.25%	66.67%	46.15%	71.43%
O52F-62_fear	Old	Female	52	62	68.33%	43.75%	93.33%	46.15%	87.50%
O53F-64_fear	Old	Female	53	64	65.52%	50.00%	73.33%	46.15%	92.86%
O56F-65_fear	Old	Female	56	65	32.76%	43.75%	26.67%	28.57%	30.77%
O8M-65_fear	Old	Male	8	65	56.90%	56.25%	60.00%	38.46%	71.43%
O12M-64_fear	Old	Male	12	64	67.24%	68.75%	73.33%	50.00%	76.92%
O15M-69_fear	Old	Male	15	69	81.67%	62.50%	93.33%	92.31%	81.25%
O17M-69_fear	Old	Male	17	69	65.52%	56.25%	66.67%	69.23%	71.43%
O20M-65_fear	Old	Male	20	65	15.52%	12.50%	26.67%	0.00%	23.08%
O21M-65_fear	Old	Male	21	65	42.11%	12.50%	66.67%	38.46%	53.85%
O35M-66_fear	Old	Male	35	66	31.03%	6.25%	66.67%	7.69%	42.86%
O42M-75_fear	Old	Male	42	75	76.67%	81.25%	80.00%	61.54%	81.25%
O50M-65_fear	Old	Male	50	65	80.70%	81.25%	80.00%	69.23%	92.31%
O55M-64_fear	Old	Male	55	64	82.76%	68.75%	93.33%	78.57%	92.31%
O58M-64_fear	Old	Male	58	64	75.00%	68.75%	80.00%	69.23%	81.25%
O59M-65_fear	Old	Male	59	65	49.12%	43.75%	53.33%	46.15%	53.85%
O63M-61_fear	Old	Male	63	61	77.19%	62.50%	93.33%	53.85%	100.00%
O64M-65_fear	Old	Male	64	65	79.31%	68.75%	86.67%	71.43%	92.31%
O65M-65_fear	Old	Male	65	65	24.14%	18.75%	46.67%	21.43%	7.69%
O66M-70_fear	Old	Male	66	70	53.45%	43.75%	46.67%	46.15%	78.57%
O67M-61_fear	Old	Male	67	61	75.86%	68.75%	86.67%	64.29%	84.62%
O68M-60_fear	Old	Male	68	60	78.33%	62.50%	93.33%	76.92%	81.25%
O69M-62_fear	Old	Male	69	62	75.00%	68.75%	93.33%	53.85%	81.25%
O70M-66_fear	Old	Male	70	66	46.67%	50.00%	80.00%	23.08%	31.25%
O71M-65_fear	Old	Male	71	65	55.17%	50.00%	66.67%	35.71%	69.23%

Image Information					Identification Score				
Image Name	Model Age Group	Model Gender	Model Code	Actual Age (Years)	All Raters	Older Female Raters	Young Female Raters	Older Male Raters	Young Male Raters
Y3F-20_disgust	Young	Female	3	20	93.33%	100.00%	100.00%	76.92%	93.75%
Y4F-19_disgust	Young	Female	4	19	96.67%	100.00%	100.00%	92.31%	93.75%
Y5F-24_disgust	Young	Female	5	24	76.67%	62.50%	86.67%	69.23%	87.50%
Y6F-23_disgust	Young	Female	6	23	77.19%	62.50%	93.33%	76.92%	76.92%
Y12F-18_disgust	Young	Female	12	18	87.93%	93.75%	100.00%	84.62%	71.43%
Y13F-20_disgust	Young	Female	13	20	84.48%	87.50%	93.33%	71.43%	84.62%
Y14F-21_disgust	Young	Female	14	21	90.00%	87.50%	93.33%	92.31%	87.50%
Y17F-33_disgust	Young	Female	17	33	60.00%	75.00%	73.33%	38.46%	50.00%
Y18F-18_disgust	Young	Female	18	18	55.65%	59.38%	53.33%	50.00%	59.26%
Y19F-19_disgust	Young	Female	19	19	61.40%	43.75%	73.33%	46.15%	84.62%
Y22F-30_disgust	Young	Female	22	30	84.48%	93.75%	93.33%	71.43%	76.92%
Y23F-30_disgust	Young	Female	23	30	65.52%	56.25%	66.67%	46.15%	92.86%
Y25F-33_disgust	Young	Female	25	33	81.03%	93.75%	93.33%	69.23%	64.29%
Y26F-32_disgust	Young	Female	26	32	85.96%	87.50%	86.67%	76.92%	92.31%
Y31F-30_disgust	Young	Female	31	30	63.16%	68.75%	73.33%	46.15%	61.54%
Y32F-20_disgust	Young	Female	32	20	78.95%	81.25%	86.67%	76.92%	69.23%
Y37F-32_disgust	Young	Female	37	32	70.18%	81.25%	73.33%	61.54%	61.54%
Y38F-24_disgust	Young	Female	38	24	77.59%	68.75%	93.33%	71.43%	76.92%
Y39F-25_disgust	Young	Female	39	25	65.52%	68.75%	73.33%	46.15%	71.43%
Y40F-28_disgust	Young	Female	40	28	86.21%	93.75%	80.00%	92.31%	78.57%
Y42F-20_disgust	Young	Female	42	20	82.46%	81.25%	80.00%	76.92%	92.31%
Y48F-23_disgust	Young	Female	48	23	78.95%	87.50%	86.67%	69.23%	69.23%
Y50F-24_disgust	Young	Female	50	24	83.05%	87.50%	86.67%	80.77%	76.67%
Y51F-23_disgust	Young	Female	51	23	85.96%	87.50%	86.67%	76.92%	92.31%
Y52F-21_disgust	Young	Female	52	21	75.00%	87.50%	73.33%	61.54%	75.00%
Y59F-23_disgust	Young	Female	59	23	75.86%	93.75%	80.00%	53.85%	71.43%
Y65F-21_disgust	Young	Female	65	21	65.52%	75.00%	60.00%	64.29%	61.54%
Y66F-26_disgust	Young	Female	66	26	89.66%	87.50%	100.00%	71.43%	100.00%
Y69F-28_disgust	Young	Female	69	28	81.67%	75.00%	93.33%	61.54%	93.75%
Y71F-20_disgust	Young	Female	71	20	62.39%	68.75%	73.33%	53.85%	51.72%
Y72F-25_disgust	Young	Female	72	25	72.41%	75.00%	60.00%	78.57%	76.92%
Y1M-19_disgust	Young	Male	1	19	87.93%	87.50%	93.33%	76.92%	92.86%
Y2M-21_disgust	Young	Male	2	21	86.67%	93.75%	100.00%	76.92%	75.00%
Y8M-27_disgust	Young	Male	8	27	75.00%	62.50%	93.33%	53.85%	87.50%
Y10M-22_disgust	Young	Male	10	22	77.59%	87.50%	80.00%	71.43%	69.23%
Y11M-20_disgust	Young	Male	11	20	79.31%	81.25%	93.33%	64.29%	76.92%
Y15M-20_disgust	Young	Male	15	20	68.97%	81.25%	80.00%	57.14%	53.85%
Y16M-21_disgust	Young	Male	16	21	87.93%	75.00%	100.00%	78.57%	100.00%
Y21M-21_disgust	Young	Male	21	21	60.34%	81.25%	60.00%	64.29%	30.77%
Y24M-19_disgust	Young	Male	24	19	77.59%	81.25%	73.33%	71.43%	84.62%
Y27M-23_disgust	Young	Male	27	23	55.65%	68.75%	46.67%	62.96%	42.31%
Y28M-20_disgust	Young	Male	28	20	87.93%	87.50%	100.00%	69.23%	92.86%
Y29M-21_disgust	Young	Male	29	21	63.79%	62.50%	66.67%	69.23%	57.14%
Y30M-25_disgust	Young	Male	30	25	51.75%	43.75%	70.00%	53.85%	38.46%
Y33M-25_disgust	Young	Male	33	25	81.20%	78.13%	83.33%	84.62%	79.31%
Y35M-20_disgust	Young	Male	35	20	52.59%	71.88%	50.00%	61.54%	25.00%
Y36M-30_disgust	Young	Male	36	30	72.41%	75.00%	73.33%	64.29%	76.92%
Y41M-19_disgust	Young	Male	41	19	57.89%	56.25%	60.00%	53.85%	61.54%
Y44M-26_disgust	Young	Male	44	26	70.69%	75.00%	76.67%	60.71%	69.23%
Y46M-18_disgust	Young	Male	46	18	52.63%	62.50%	53.33%	53.85%	38.46%
Y47M-23_disgust	Young	Male	47	23	78.33%	87.50%	100.00%	61.54%	62.50%
Y49M-23_disgust	Young	Male	49	23	63.16%	56.25%	60.00%	69.23%	69.23%
Y53M-23_disgust	Young	Male	53	23	75.44%	75.00%	80.00%	61.54%	84.62%
Y54M-26_disgust	Young	Male	54	26	50.00%	75.00%	60.00%	21.43%	38.46%
Y55M-24_disgust	Young	Male	55	24	70.69%	81.25%	80.00%	61.54%	57.14%
Y56M-24_disgust	Young	Male	56	24	75.86%	75.00%	86.67%	76.92%	64.29%
Y57M-23_disgust	Young	Male	57	23	75.86%	87.50%	73.33%	69.23%	71.43%
Y58M-22_disgust	Young	Male	58	22	71.93%	87.50%	73.33%	61.54%	61.54%
Y60M-24_disgust	Young	Male	60	24	82.76%	75.00%	93.33%	64.29%	100.00%
Y67M-19_disgust	Young	Male	67	19	93.10%	87.50%	100.00%	92.31%	92.86%
Y68M-30_disgust	Young	Male	68	30	83.33%	93.75%	73.33%	76.92%	87.50%
Y75M-30_disgust	Young	Male	75	30	48.33%	43.75%	46.67%	46.15%	56.25%
O4F-76_disgust	Old	Female	4	76	70.00%	62.50%	86.67%	53.85%	75.00%
O7F-65_disgust	Old	Female	7	65	81.03%	75.00%	100.00%	76.92%	71.43%
O9F-64_disgust	Old	Female	9	64	66.67%	62.50%	80.00%	46.15%	76.92%
O10F-60_disgust	Old	Female	10	60	54.39%	31.25%	86.67%	23.08%	76.92%
O16F-64_disgust	Old	Female	16	64	63.79%	37.50%	86.67%	69.23%	64.29%
O19F-60_disgust	Old	Female	19	60	60.34%	62.50%	60.00%	61.54%	57.14%
O22F-61_disgust	Old	Female	22	61	74.14%	87.50%	73.33%	69.23%	64.29%
O23F-66_disgust	Old	Female	23	66	79.31%	87.50%	86.67%	50.00%	92.31%
O24F-62_disgust	Old	Female	24	62	58.62%	68.75%	53.33%	21.43%	92.31%
O26F-64_disgust	Old	Female	26	64	50.00%	25.00%	73.33%	35.71%	81.25%
O27F-65_disgust	Old	Female	27	65	88.33%	93.75%	100.00%	76.92%	81.25%
O28F-64_disgust	Old	Female	28	64	55.17%	56.25%	73.33%	46.15%	42.86%
O29F-63_disgust	Old	Female	29	63	75.86%	87.50%	86.67%	53.85%	71.43%
O34F-65_disgust	Old	Female	34	65	77.12%	84.38%	70.00%	84.62%	70.00%
O38F-65_disgust	Old	Female	38	65	96.67%	87.50%	100.00%	100.00%	100.00%
O40F-61_disgust	Old	Female	40	61	79.31%	68.75%	93.33%	69.23%	85.71%
O41F-72_disgust	Old	Female	41	72	77.19%	75.00%	86.67%	69.23%	76.92%
O43F-62_disgust	Old	Female	43	62	81.03%	87.50%	86.67%	69.23%	78.57%
O45F-65_disgust	Old	Female	45	65	77.59%	81.25%	73.33%	69.23%	85.71%
O47F-60_disgust	Old	Female	47	60	62.07%	62.50%	66.67%	61.54%	57.14%
O48F-65_disgust	Old	Female	48	65	55.00%	75.00%	53.33%	53.85%	37.50%
O49F-65_disgust	Old	Female	49	65	84.48%	81.25%	93.33%	71.43%	92.31%
O51F-60_disgust	Old	Female	51	60	51.72%	31.25%	66.67%	46.15%	64.29%
O52F-62_disgust	Old	Female	52	62	82.61%	87.50%	80.00%	80.77%	81.48%
O53F-64_disgust	Old	Female	53	64	69.83%	62.50%	86.67%	55.56%	74.07%
O56F-65_disgust	Old	Female	56	65	45.61%	56.25%	53.33%	23.08%	46.15%
O8M-65_disgust	Old	Male	8	65	86.67%	75.00%	93.33%	76.92%	100.00%
O12M-64_disgust	Old	Male	12	64	63.33%	87.50%	53.33%	53.85%	56.25%
O15M-69_disgust	Old	Male	15	69	75.44%	62.50%	86.67%	69.23%	84.62%
O17M-69_disgust	Old	Male	17	69	55.00%	56.25%	60.00%	38.46%	62.50%
O20M-65_disgust	Old	Male	20	65	59.65%	62.50%	73.33%	53.85%	46.15%
O21M-65_disgust	Old	Male	21	65	78.33%	68.75%	93.33%	53.85%	93.75%
O35M-66_disgust	Old	Male	35	66	86.21%	93.75%	93.33%	78.57%	76.92%
O42M-75_disgust	Old	Male	42	75	51.67%	68.75%	46.67%	30.77%	56.25%
O50M-65_disgust	Old	Male	50	65	70.00%	50.00%	86.67%	53.85%	87.50%
O55M-64_disgust	Old	Male	55	64	38.60%	25.00%	46.67%	46.15%	38.46%
O58M-64_disgust	Old	Male	58	64	52.59%	56.25%	60.00%	57.69%	35.71%
O59M-65_disgust	Old	Male	59	65	70.69%	56.25%	93.33%	35.71%	100.00%
O63M-61_disgust	Old	Male	63	61	46.09%	37.50%	50.00%	57.69%	40.74%
O64M-65_disgust	Old	Male	64	65	75.00%	68.75%	86.67%	69.23%	75.00%
O65M-65_disgust	Old	Male	65	65	82.46%	62.50%	100.00%	84.62%	84.62%
O66M-70_disgust	Old	Male	66	70	82.76%	75.00%	80.00%	76.92%	100.00%
O67M-61_disgust	Old	Male	67	61	43.86%	43.75%	53.33%	30.77%	46.15%
O68M-60_disgust	Old	Male	68	60	45.61%	31.25%	66.67%	53.85%	30.77%
O69M-62_disgust	Old	Male	69	62	78.63%	71.88%	90.00%	65.38%	86.21%
O70M-66_disgust	Old	Male	70	66	73.33%	50.00%	86.67%	61.54%	93.75%
O71M-65_disgust	Old	Male	71	65	82.76%	75.00%	86.67%	84.62%	85.71%

Image Information					Identification Score				
Image Name	Model Age Group	Model Gender	Model Code	Actual Age (Years)	All Raters	Older Female Raters	Young Female Raters	Older Male Raters	Young Male Raters
Y3F-20_surprise	Young	Female	3	20	84.48%	75.00%	100.00%	84.62%	78.57%
Y4F-19_surprise	Young	Female	4	19	63.79%	56.25%	66.67%	76.92%	57.14%
Y5F-24_surprise	Young	Female	5	24	94.74%	87.50%	100.00%	92.31%	100.00%
Y6F-23_surprise	Young	Female	6	23	89.47%	75.00%	100.00%	92.31%	92.31%
Y12F-18_surprise	Young	Female	12	18	90.00%	87.50%	93.33%	76.92%	100.00%
Y13F-20_surprise	Young	Female	13	20	89.47%	81.25%	93.33%	92.31%	92.31%
Y14F-21_surprise	Young	Female	14	21	96.49%	87.50%	100.00%	100.00%	100.00%
Y17F-33_surprise	Young	Female	17	33	91.67%	87.50%	93.33%	92.31%	93.75%
Y18F-18_surprise	Young	Female	18	18	82.76%	43.75%	100.00%	92.31%	100.00%
Y19F-19_surprise	Young	Female	19	19	75.00%	75.00%	80.00%	53.85%	87.50%
Y22F-30_surprise	Young	Female	22	30	75.44%	56.25%	80.00%	84.62%	84.62%
Y23F-30_surprise	Young	Female	23	30	77.59%	56.25%	93.33%	64.29%	100.00%
Y25F-33_surprise	Young	Female	25	33	68.33%	62.50%	66.67%	69.23%	75.00%
Y26F-32_surprise	Young	Female	26	32	84.48%	68.75%	93.33%	85.71%	92.31%
Y31F-30_surprise	Young	Female	31	30	70.69%	68.75%	86.67%	42.86%	84.62%
Y32F-20_surprise	Young	Female	32	20	94.74%	87.50%	93.33%	100.00%	100.00%
Y37F-32_surprise	Young	Female	37	32	84.48%	75.00%	93.33%	69.23%	100.00%
Y38F-24_surprise	Young	Female	38	24	81.03%	62.50%	100.00%	71.43%	92.31%
Y39F-25_surprise	Young	Female	39	25	77.19%	56.25%	86.67%	84.62%	84.62%
Y40F-28_surprise	Young	Female	40	28	63.33%	50.00%	73.33%	53.85%	75.00%
Y42F-20_surprise	Young	Female	42	20	91.67%	87.50%	100.00%	84.62%	93.75%
Y48F-23_surprise	Young	Female	48	23	75.00%	68.75%	86.67%	53.85%	87.50%
Y50F-24_surprise	Young	Female	50	24	94.83%	100.00%	100.00%	85.71%	92.31%
Y51F-23_surprise	Young	Female	51	23	87.93%	68.75%	100.00%	85.71%	100.00%
Y52F-21_surprise	Young	Female	52	21	71.93%	31.25%	86.67%	84.62%	92.31%
Y59F-23_surprise	Young	Female	59	23	92.98%	81.25%	93.33%	100.00%	100.00%
Y65F-21_surprise	Young	Female	65	21	81.03%	81.25%	73.33%	84.62%	85.71%
Y66F-26_surprise	Young	Female	66	26	93.33%	87.50%	100.00%	92.31%	93.75%
Y69F-28_surprise	Young	Female	69	28	86.67%	75.00%	86.67%	100.00%	87.50%
Y71F-20_surprise	Young	Female	71	20	86.67%	62.50%	93.33%	92.31%	100.00%
Y72F-25_surprise	Young	Female	72	25	84.21%	62.50%	100.00%	84.62%	92.31%
Y1M-19_surprise	Young	Male	1	19	82.46%	81.25%	86.67%	84.62%	76.92%
Y2M-21_surprise	Young	Male	2	21	81.03%	50.00%	93.33%	92.31%	92.86%
Y8M-27_surprise	Young	Male	8	27	89.47%	93.75%	86.67%	92.31%	84.62%
Y10M-22_surprise	Young	Male	10	22	94.83%	93.75%	100.00%	84.62%	100.00%
Y11M-20_surprise	Young	Male	11	20	89.47%	81.25%	86.67%	100.00%	92.31%
Y15M-20_surprise	Young	Male	15	20	84.21%	81.25%	100.00%	76.92%	76.92%
Y16M-21_surprise	Young	Male	16	21	89.47%	87.50%	93.33%	84.62%	92.31%
Y21M-21_surprise	Young	Male	21	21	72.41%	43.75%	86.67%	69.23%	92.86%
Y24M-19_surprise	Young	Male	24	19	75.86%	50.00%	100.00%	64.29%	92.31%
Y27M-23_surprise	Young	Male	27	23	96.67%	87.50%	100.00%	100.00%	100.00%
Y28M-20_surprise	Young	Male	28	20	67.24%	62.50%	80.00%	57.14%	69.23%
Y29M-21_surprise	Young	Male	29	21	80.70%	68.75%	86.67%	69.23%	100.00%
Y30M-25_surprise	Young	Male	30	25	71.93%	50.00%	86.67%	69.23%	84.62%
Y33M-25_surprise	Young	Male	33	25	79.31%	75.00%	80.00%	92.31%	71.43%
Y35M-20_surprise	Young	Male	35	20	81.67%	75.00%	86.67%	76.92%	87.50%
Y36M-30_surprise	Young	Male	36	30	96.49%	93.75%	100.00%	100.00%	92.31%
Y41M-19_surprise	Young	Male	41	19	92.98%	87.50%	100.00%	92.31%	92.31%
Y44M-26_surprise	Young	Male	44	26	63.33%	62.50%	60.00%	61.54%	68.75%
Y46M-18_surprise	Young	Male	46	18	70.00%	81.25%	60.00%	53.85%	81.25%
Y47M-23_surprise	Young	Male	47	23	91.38%	93.75%	100.00%	85.71%	84.62%
Y49M-23_surprise	Young	Male	49	23	77.59%	50.00%	100.00%	76.92%	85.71%
Y53M-23_surprise	Young	Male	53	23	94.83%	93.75%	100.00%	84.62%	100.00%
Y54M-26_surprise	Young	Male	54	26	82.76%	62.50%	100.00%	85.71%	84.62%
Y55M-24_surprise	Young	Male	55	24	72.41%	50.00%	100.00%	64.29%	76.92%
Y56M-24_surprise	Young	Male	56	24	77.59%	75.00%	93.33%	71.43%	69.23%
Y57M-23_surprise	Young	Male	57	23	68.42%	62.50%	66.67%	76.92%	69.23%
Y58M-22_surprise	Young	Male	58	22	71.93%	62.50%	73.33%	76.92%	76.92%
Y60M-24_surprise	Young	Male	60	24	94.83%	81.25%	100.00%	100.00%	100.00%
Y67M-19_surprise	Young	Male	67	19	93.10%	93.75%	100.00%	84.62%	92.86%
Y68M-30_surprise	Young	Male	68	30	91.38%	81.25%	93.33%	92.86%	100.00%
Y74M-31_surprise	Young	Male	74	31	76.67%	75.00%	93.33%	46.15%	87.50%
Y75M-30_surprise	Young	Male	75	30	93.33%	100.00%	93.33%	92.31%	87.50%
O4F-76_surprise	Old	Female	4	76	79.31%	62.50%	86.67%	85.71%	84.62%
O7F-65_surprise	Old	Female	7	65	79.31%	62.50%	93.33%	76.92%	85.71%
O9F-64_surprise	Old	Female	9	64	76.67%	75.00%	86.67%	69.23%	75.00%
O10F-60_surprise	Old	Female	10	60	93.10%	87.50%	93.33%	92.31%	100.00%
O16F-64_surprise	Old	Female	16	64	84.48%	75.00%	100.00%	64.29%	100.00%
O19F-60_surprise	Old	Female	19	60	89.66%	81.25%	93.33%	84.62%	100.00%
O22F-61_surprise	Old	Female	22	61	90.00%	87.50%	93.33%	76.92%	100.00%
O23F-66_surprise	Old	Female	23	66	63.79%	50.00%	93.33%	50.00%	81.25%
O24F-62_surprise	Old	Female	24	62	67.24%	43.75%	86.67%	64.29%	76.92%
O26F-64_surprise	Old	Female	26	64	78.95%	56.25%	86.67%	92.31%	84.62%
O27F-65_surprise	Old	Female	27	65	94.83%	87.50%	100.00%	100.00%	92.31%
O28F-64_surprise	Old	Female	28	64	74.14%	37.50%	86.67%	84.62%	92.86%
O29F-63_surprise	Old	Female	29	63	70.18%	43.75%	93.33%	61.54%	84.62%
O34F-65_surprise	Old	Female	34	65	71.93%	50.00%	93.33%	69.23%	76.92%
O38F-65_surprise	Old	Female	38	65	61.40%	43.75%	66.67%	69.23%	69.23%
O40F-61_surprise	Old	Female	40	61	51.72%	43.75%	53.33%	61.54%	50.00%
O41F-72_surprise	Old	Female	41	72	82.76%	68.75%	93.33%	92.31%	78.57%
O43F-62_surprise	Old	Female	43	62	68.97%	50.00%	93.33%	53.85%	78.57%
O45F-65_surprise	Old	Female	45	65	96.49%	93.75%	100.00%	92.31%	100.00%
O47F-60_surprise	Old	Female	47	60	91.67%	93.75%	100.00%	84.62%	87.50%
O48F-65_surprise	Old	Female	48	65	81.67%	62.50%	93.33%	76.92%	93.75%
O49F-65_surprise	Old	Female	49	65	56.14%	56.25%	66.67%	53.85%	46.15%
O51F-60_surprise	Old	Female	51	60	81.03%	75.00%	86.67%	78.57%	84.62%
O52F-62_surprise	Old	Female	52	62	75.00%	46.88%	90.00%	77.78%	88.89%
O53F-64_surprise	Old	Female	53	64	58.33%	62.50%	66.67%	38.46%	62.50%
O56F-65_surprise	Old	Female	56	65	90.00%	87.50%	100.00%	76.92%	93.75%
O8M-65_surprise	Old	Male	8	65	85.00%	81.25%	93.33%	84.62%	81.25%
O12M-64_surprise	Old	Male	12	64	78.33%	75.00%	86.67%	53.85%	93.75%
O15M-69_surprise	Old	Male	15	69	81.67%	68.75%	93.33%	69.23%	93.75%
O17M-69_surprise	Old	Male	17	69	92.98%	81.25%	100.00%	92.31%	100.00%
O20M-65_surprise	Old	Male	20	65	89.66%	75.00%	100.00%	85.71%	100.00%
O21M-65_surprise	Old	Male	21	65	81.36%	84.38%	80.00%	70.37%	89.66%
O35M-66_surprise	Old	Male	35	66	79.31%	68.75%	93.33%	76.92%	78.57%
O42M-75_surprise	Old	Male	42	75	77.59%	62.50%	93.33%	71.43%	84.62%
O50M-65_surprise	Old	Male	50	65	48.28%	31.25%	66.67%	35.71%	61.54%
O55M-64_surprise	Old	Male	55	64	46.55%	37.50%	73.33%	21.43%	53.85%
O58M-64_surprise	Old	Male	58	64	80.00%	81.25%	86.67%	69.23%	81.25%
O59M-65_surprise	Old	Male	59	65	63.79%	62.50%	73.33%	57.14%	61.54%
O63M-61_surprise	Old	Male	63	61	89.66%	68.75%	100.00%	92.86%	100.00%
O64M-65_surprise	Old	Male	64	65	65.52%	31.25%	100.00%	64.29%	69.23%
O65M-65_surprise	Old	Male	65	65	77.59%	75.00%	86.67%	71.43%	76.92%
O66M-70_surprise	Old	Male	66	70	86.21%	87.50%	93.33%	84.62%	78.57%
O67M-61_surprise	Old	Male	67	61	63.79%	62.50%	66.67%	61.54%	64.29%
O68M-60_surprise	Old	Male	68	60	68.97%	62.50%	80.00%	69.23%	64.29%
O69M-62_surprise	Old	Male	69	62	84.48%	75.00%	86.67%	76.92%	100.00%
O70M-66_surprise	Old	Male	70	66	85.00%	87.50%	73.33%	84.62%	93.75%
O71M-65_surprise	Old	Male	71	65	58.62%	50.00%	80.00%	42.86%	61.54%
