# Supplementary material for: Tsinghua facial expression database – A database of facial expressions in Chinese young and older women and men: Development and validation
Source: PLoS One. 2020 Apr 15;15(4):e0231304. doi: 10.1371/journal.pone.0231304 (PMC7159817; doi:10.1371/journal.pone.0231304)
Supplement: S3 Table — (PDF) [file pone.0231304.s003.pdf]

Image Information					Perceived emotional intensity (1=lowerst, 5=highest)				
Image Name	Model Age group	Model Gender	Model Code	Actual Age (Years)	All Raters	Older Female raters	Young Female raters	Older Male raters	Younger Male raters
Y3F-20_happy	Young	Female	3	20	4.44	4.75	4.13	4.69	4.15
Y4F-19_happy	Young	Female	4	19	4.40	4.50	4.53	4.50	4.00
Y5F-24_happy	Young	Female	5	24	4.27	4.38	4.33	4.31	4.06
Y6F-23_happy	Young	Female	6	23	4.21	4.50	4.20	4.31	3.79
Y12F-18_happy	Young	Female	12	18	4.38	4.50	4.33	4.46	4.25
Y13F-20_happy	Young	Female	13	20	4.39	4.56	4.27	4.54	4.15
Y14F-21_happy	Young	Female	14	21	4.52	4.56	4.47	4.69	4.38
Y17F-33_happy	Young	Female	17	33	4.28	4.50	4.13	4.64	3.77
Y18F-18_happy	Young	Female	18	18	4.30	4.56	4.07	4.23	4.31
Y19F-19_happy	Young	Female	19	19	4.41	4.69	4.47	4.57	3.85
Y22F-30_happy	Young	Female	22	30	4.50	4.75	4.33	4.54	4.36
Y23F-30_happy	Young	Female	23	30	4.58	4.88	4.60	4.54	4.23
Y25F-33_happy	Young	Female	25	33	4.26	4.50	4.20	4.50	3.77
Y26F-32_happy	Young	Female	26	32	4.05	4.38	3.93	4.46	3.43
Y31F-30_happy	Young	Female	31	30	4.57	4.69	4.53	4.69	4.36
Y32F-20_happy	Young	Female	32	20	4.39	4.50	4.33	4.54	4.15
Y37F-32_happy	Young	Female	37	32	4.02	4.56	3.53	4.38	3.54
Y38F-24_happy	Young	Female	38	24	4.05	4.44	3.80	4.23	3.69
Y39F-25_happy	Young	Female	39	25	3.97	4.31	3.93	4.08	3.50
Y40F-28_happy	Young	Female	40	28	3.95	4.13	3.73	4.15	3.79
Y42F-20_happy	Young	Female	42	20	4.08	4.25	3.93	4.31	3.88
Y48F-23_happy	Young	Female	48	23	4.00	4.31	3.87	4.43	3.31
Y50F-24_happy	Young	Female	50	24	3.82	4.13	3.40	4.15	3.63
Y51F-23_happy	Young	Female	51	23	3.42	3.94	3.13	3.92	2.62
Y52F-21_happy	Young	Female	52	21	4.17	4.50	4.00	4.57	3.54
Y59F-23_happy	Young	Female	59	23	4.35	4.31	4.33	4.54	4.25
Y65F-21_happy	Young	Female	65	21	4.33	4.38	4.33	4.62	4.00
Y66F-26_happy	Young	Female	66	26	4.33	4.56	4.00	4.54	4.23
Y69F-28_happy	Young	Female	69	28	3.60	4.00	3.33	4.08	3.00
Y71F-20_happy	Young	Female	71	20	4.19	4.50	4.20	4.50	3.46
Y72F-25_happy	Young	Female	72	25	3.62	3.81	3.47	4.31	2.93
Y1M-19_happy	Young	Male	1	19	3.48	4.06	3.33	3.86	2.54
Y2M-21_happy	Young	Male	2	21	3.98	4.06	4.20	4.29	3.31
Y8M-27_happy	Young	Male	8	27	4.42	4.75	4.27	4.38	4.23
Y10M-22_happy	Young	Male	10	22	3.88	4.25	3.60	4.31	3.44
Y11M-20_happy	Young	Male	11	20	4.42	4.69	4.27	4.62	4.08
Y15M-20_happy	Young	Male	15	20	3.81	4.06	3.80	4.31	3.07
Y16M-21_happy	Young	Male	16	21	4.10	4.38	4.07	4.36	3.54
Y21M-21_happy	Young	Male	21	21	4.32	4.19	4.53	4.38	4.19
Y24M-19_happy	Young	Male	24	19	4.28	4.31	4.33	4.46	4.06
Y27M-23_happy	Young	Male	27	23	4.49	4.94	4.20	4.62	4.15
Y28M-20_happy	Young	Male	28	20	3.48	3.94	3.47	3.92	2.69
Y29M-21_happy	Young	Male	29	21	4.43	4.56	4.40	4.57	4.15
Y30M-25_happy	Young	Male	30	25	4.32	4.63	4.13	4.46	4.00
Y33M-25_happy	Young	Male	33	25	4.11	4.63	3.67	4.46	3.62
Y35M-20_happy	Young	Male	35	20	3.90	4.25	3.73	4.21	3.31
Y36M-30_happy	Young	Male	36	30	4.12	4.25	4.00	4.38	3.88
Y41M-19_happy	Young	Male	41	19	4.53	4.81	4.33	4.69	4.23
Y44M-26_happy	Young	Male	44	26	4.07	4.38	4.07	4.23	3.63
Y46M-18_happy	Young	Male	46	18	3.78	4.25	3.53	4.21	3.00
Y47M-23_happy	Young	Male	47	23	4.02	4.25	3.80	4.38	3.69
Y49M-23_happy	Young	Male	49	23	4.02	4.19	4.13	4.23	3.56
Y53M-23_happy	Young	Male	53	23	4.45	4.50	4.40	4.46	4.44
Y54M-26_happy	Young	Male	54	26	3.79	4.13	3.87	4.07	3.00
Y55M-24_happy	Young	Male	55	24	4.00	4.25	3.80	4.38	3.54
Y56M-24_happy	Young	Male	56	24	4.15	4.25	4.07	4.46	3.88
Y57M-23_happy	Young	Male	57	23	4.44	4.69	4.47	4.38	4.15
Y58M-22_happy	Young	Male	58	22	3.54	4.38	3.00	4.00	2.69
Y60M-24_happy	Young	Male	60	24	4.04	4.19	3.87	4.46	3.62
Y67M-19_happy	Young	Male	67	19	4.07	4.19	4.00	4.38	3.75
Y68M-30_happy	Young	Male	68	30	4.17	4.31	4.27	4.31	3.81
Y74M-31_happy	Young	Male	74	31	3.95	4.06	3.80	4.38	3.54
Y75M-30_happy	Young	Male	75	30	4.47	4.75	4.33	4.62	4.15
O4F-76_happy	Old	Female	4	76	4.41	4.63	4.33	4.62	4.07
O7F-65_happy	Old	Female	7	65	4.05	4.31	4.07	4.29	3.46
O9F-64_happy	Old	Female	9	64	4.44	4.75	4.20	4.77	4.00
O10F-60_happy	Old	Female	10	60	4.34	4.56	4.33	4.62	3.86
O16F-64_happy	Old	Female	16	64	4.16	4.38	4.13	4.15	3.93
O19F-60_happy	Old	Female	19	60	4.12	4.31	4.20	4.38	3.63
O22F-61_happy	Old	Female	22	61	4.34	4.56	4.27	4.57	3.92
O23F-66_happy	Old	Female	23	66	4.29	4.44	4.47	4.36	3.85
O24F-62_happy	Old	Female	24	62	3.83	3.94	3.87	4.29	3.15
O26F-64_happy	Old	Female	26	64	3.74	4.25	3.40	4.21	3.00
O27F-65_happy	Old	Female	27	65	4.35	4.50	4.33	4.31	4.25
O28F-64_happy	Old	Female	28	64	3.50	3.81	3.27	4.07	2.77
O29F-63_happy	Old	Female	29	63	4.52	4.50	4.60	4.46	4.50
O34F-65_happy	Old	Female	34	65	4.24	4.56	4.13	4.29	3.92
O38F-65_happy	Old	Female	38	65	4.52	4.63	4.47	4.62	4.38
O40F-61_happy	Old	Female	40	61	4.03	4.13	4.00	4.14	3.85
O41F-72_happy	Old	Female	41	72	4.35	4.69	4.33	4.46	3.94
O43F-62_happy	Old	Female	43	62	4.23	4.31	4.33	4.31	4.00
O45F-65_happy	Old	Female	45	65	3.91	4.25	3.47	4.31	3.62
O47F-60_happy	Old	Female	47	60	4.13	4.38	3.80	4.46	3.94
O48F-65_happy	Old	Female	48	65	4.19	4.63	3.67	4.62	3.85
O49F-65_happy	Old	Female	49	65	3.72	4.06	3.13	4.23	3.46
O51F-60_happy	Old	Female	51	60	4.12	4.19	4.20	4.29	3.77
O52F-62_happy	Old	Female	52	62	3.47	3.81	3.40	4.00	2.54
O53F-64_happy	Old	Female	53	64	4.09	4.44	4.20	4.23	3.43
O56F-65_happy	Old	Female	56	65	3.91	4.06	4.13	4.00	3.38
O8M-65_happy	Old	Male	8	65	4.57	4.81	4.47	4.64	4.31
O12M-64_happy	Old	Male	12	64	3.85	4.19	3.53	4.15	3.56
O15M-69_happy	Old	Male	15	69	4.23	4.44	3.93	4.46	4.08
O17M-69_happy	Old	Male	17	69	4.23	4.63	4.07	4.54	3.75
O20M-65_happy	Old	Male	20	65	4.12	4.50	3.93	4.46	3.54
O21M-65_happy	Old	Male	21	65	4.07	4.25	4.07	4.29	3.62
O35M-66_happy	Old	Male	35	66	4.62	4.81	4.73	4.69	4.21
O42M-75_happy	Old	Male	42	75	4.23	4.38	4.07	4.38	4.08
O50M-65_happy	Old	Male	50	65	3.86	4.19	3.80	4.36	3.00
O55M-64_happy	Old	Male	55	64	4.02	4.31	3.73	4.46	3.54
O58M-64_happy	Old	Male	58	64	4.19	4.44	4.07	4.57	3.62
O59M-65_happy	Old	Male	59	65	3.77	3.94	3.53	4.23	3.44
O63M-61_happy	Old	Male	63	61	3.70	3.94	3.47	4.15	3.31
O64M-65_happy	Old	Male	64	65	4.16	4.38	4.13	4.43	3.62
O65M-65_happy	Old	Male	65	65	4.47	4.75	4.27	4.62	4.23
O66M-70_happy	Old	Male	66	70	4.32	4.50	4.20	4.46	4.13
O67M-61_happy	Old	Male	67	61	4.12	4.44	4.00	4.38	3.62
O68M-60_happy	Old	Male	68	60	3.47	3.94	3.13	3.69	3.08
O69M-62_happy	Old	Male	69	62	4.36	4.56	4.20	4.54	4.14
O70M-66_happy	Old	Male	70	66	4.25	4.56	4.13	4.31	3.92
O71M-65_happy	Old	Male	71	65	4.14	4.38	4.07	4.46	3.64

Image Information					Perceived emotional intensity (1=lowerst, 5=highest)				
Image Name	Model Age group	Model Gender	Model Code	Actual Age (Years)	All Raters	Older Female raters	Young Female raters	Older Male raters	Younger Male raters
Y3F-20_content	Young	Female	3	20	3.61	3.94	3.33	4.00	3.15
Y4F-19_content	Young	Female	4	19	3.29	3.63	3.00	3.77	2.79
Y5F-24_content	Young	Female	5	24	4.09	4.19	4.00	4.38	3.79
Y6F-23_content	Young	Female	6	23	4.02	4.25	3.73	4.23	3.88
Y12F-18_content	Young	Female	12	18	3.80	4.13	3.73	4.15	3.25
Y13F-20_content	Young	Female	13	20	3.84	3.81	3.73	4.31	3.57
Y14F-21_content	Young	Female	14	21	4.32	4.31	4.27	4.46	4.25
Y17F-33_content	Young	Female	17	33	4.09	4.31	4.00	4.38	3.64
Y18F-18_content	Young	Female	18	18	3.97	3.94	4.13	4.23	3.63
Y19F-19_content	Young	Female	19	19	3.86	4.38	3.67	4.23	3.08
Y22F-30_content	Young	Female	22	30	3.78	4.06	3.67	4.21	3.08
Y23F-30_content	Young	Female	23	30	4.07	4.50	3.80	4.23	3.69
Y25F-33_content	Young	Female	25	33	3.93	4.31	3.67	4.38	3.31
Y26F-32_content	Young	Female	26	32	3.74	4.19	3.27	4.08	3.38
Y31F-30_content	Young	Female	31	30	3.71	3.94	3.67	3.92	3.29
Y32F-20_content	Young	Female	32	20	4.08	4.13	4.13	4.38	3.75
Y37F-32_content	Young	Female	37	32	4.17	4.25	4.20	4.31	3.93
Y38F-24_content	Young	Female	38	24	3.74	4.00	3.93	3.86	3.08
Y39F-25_content	Young	Female	39	25	3.74	3.94	3.87	4.07	3.00
Y40F-28_content	Young	Female	40	28	3.75	3.94	3.40	4.23	3.50
Y42F-20_content	Young	Female	42	20	3.75	4.25	3.27	4.23	3.23
Y48F-23_content	Young	Female	48	23	3.60	3.81	3.40	4.15	3.13
Y50F-24_content	Young	Female	50	24	3.11	3.81	2.53	3.69	2.31
Y51F-23_content	Young	Female	51	23	3.65	4.31	3.27	4.08	2.85
Y52F-21_content	Young	Female	52	21	3.46	3.88	3.13	3.77	3.00
Y59F-23_content	Young	Female	59	23	4.07	4.13	3.93	4.38	3.88
Y65F-21_content	Young	Female	65	21	3.72	3.81	3.73	4.15	3.25
Y66F-26_content	Young	Female	66	26	3.80	3.75	3.67	4.38	3.50
Y69F-28_content	Young	Female	69	28	3.73	3.88	3.80	3.92	3.38
Y71F-20_content	Young	Female	71	20	3.28	3.50	2.93	3.77	2.93
Y72F-25_content	Young	Female	72	25	3.51	4.25	2.93	3.85	2.92
Y1M-19_content	Young	Male	1	19	3.17	3.38	3.13	3.77	2.50
Y2M-21_content	Young	Male	2	21	3.91	4.13	4.00	4.15	3.36
Y8M-27_content	Young	Male	8	27	4.26	4.50	4.27	4.43	3.77
Y10M-22_content	Young	Male	10	22	3.24	3.50	3.47	3.69	2.29
Y11M-20_content	Young	Male	11	20	4.21	4.44	4.53	4.07	3.69
Y15M-20_content	Young	Male	15	20	3.66	4.06	3.33	4.07	3.08
Y16M-21_content	Young	Male	16	21	4.08	4.31	4.07	4.31	3.69
Y21M-21_content	Young	Male	21	21	3.74	4.13	3.40	4.07	3.31
Y24M-19_content	Young	Male	24	19	3.33	3.38	3.20	3.92	2.86
Y27M-23_content	Young	Male	27	23	4.30	4.31	4.47	4.69	3.81
Y28M-20_content	Young	Male	28	20	3.28	3.50	3.07	3.77	2.88
Y29M-21_content	Young	Male	29	21	4.26	4.44	4.40	4.62	3.54
Y30M-25_content	Young	Male	30	25	4.09	4.31	4.07	4.31	3.62
Y33M-25_content	Young	Male	33	25	3.81	4.06	3.93	4.08	3.14
Y35M-20_content	Young	Male	35	20	4.00	4.38	4.07	4.08	3.43
Y36M-30_content	Young	Male	36	30	3.74	3.88	3.60	4.15	3.36
Y41M-19_content	Young	Male	41	19	3.12	3.44	2.73	3.46	2.88
Y44M-26_content	Young	Male	44	26	3.79	4.13	4.00	3.86	3.08
Y46M-18_content	Young	Male	46	18	3.67	4.00	3.47	4.08	3.08
Y47M-23_content	Young	Male	47	23	3.74	4.13	3.47	4.08	3.23
Y49M-23_content	Young	Male	49	23	4.09	4.13	4.13	4.08	4.00
Y53M-23_content	Young	Male	53	23	3.72	3.81	3.73	4.15	3.21
Y54M-26_content	Young	Male	54	26	3.52	3.81	3.47	3.86	2.85
Y55M-24_content	Young	Male	55	24	3.95	4.25	3.87	4.23	3.50
Y56M-24_content	Young	Male	56	24	3.52	3.75	3.53	4.00	2.79
Y57M-23_content	Young	Male	57	23	3.83	3.94	3.93	3.85	3.63
Y58M-22_content	Young	Male	58	22	4.07	4.25	4.13	4.23	3.64
Y60M-24_content	Young	Male	60	24	3.95	4.25	3.93	4.15	3.50
Y67M-19_content	Young	Male	67	19	3.45	3.50	3.47	4.00	2.94
Y68M-30_content	Young	Male	68	30	4.05	4.19	4.20	4.00	3.77
Y74M-31_content	Young	Male	74	31	3.02	3.69	2.60	3.69	2.00
Y75M-30_content	Young	Male	75	30	3.29	3.69	3.00	3.71	2.69
O4F-76_content	Old	Female	4	76	3.41	3.63	3.20	4.08	2.79
O7F-65_content	Old	Female	7	65	3.16	3.19	3.07	3.57	2.77
O9F-64_content	Old	Female	9	64	3.80	4.00	3.87	4.00	3.38
O10F-60_content	Old	Female	10	60	3.91	4.38	3.40	4.23	3.62
O16F-64_content	Old	Female	16	64	3.21	3.63	2.73	4.08	2.43
O19F-60_content	Old	Female	19	60	3.95	4.19	3.80	4.23	3.57
O22F-61_content	Old	Female	22	61	3.93	4.06	4.07	4.15	3.43
O23F-66_content	Old	Female	23	66	3.07	3.13	3.00	3.46	2.75
O24F-62_content	Old	Female	24	62	3.93	3.88	4.13	4.54	3.31
O26F-64_content	Old	Female	26	64	2.93	3.31	2.47	3.50	2.38
O27F-65_content	Old	Female	27	65	3.53	4.00	3.13	3.85	3.14
O28F-64_content	Old	Female	28	64	3.45	3.69	3.27	4.08	2.88
O29F-63_content	Old	Female	29	63	3.91	4.06	3.80	4.08	3.71
O34F-65_content	Old	Female	34	65	3.55	3.69	3.40	4.23	2.93
O38F-65_content	Old	Female	38	65	3.74	3.81	3.80	4.21	3.08
O40F-61_content	Old	Female	40	61	3.09	3.69	2.73	3.54	2.31
O41F-72_content	Old	Female	41	72	4.05	4.25	4.13	4.38	3.43
O43F-62_content	Old	Female	43	62	3.29	3.81	3.40	3.50	2.31
O45F-65_content	Old	Female	45	65	4.02	4.13	3.87	4.38	3.75
O47F-60_content	Old	Female	47	60	3.72	4.06	3.33	4.00	3.50
O48F-65_content	Old	Female	48	65	3.87	4.13	3.67	4.23	3.50
O49F-65_content	Old	Female	49	65	3.19	3.06	3.13	3.92	2.71
O51F-60_content	Old	Female	51	60	4.04	4.38	3.73	4.46	3.54
O52F-62_content	Old	Female	52	62	3.71	3.81	3.80	4.00	3.15
O53F-64_content	Old	Female	53	64	3.72	4.13	3.13	4.31	3.31
O56F-65_content	Old	Female	56	65	3.33	3.88	2.93	3.62	2.94
O8M-65_content	Old	Male	8	65	3.97	4.13	3.93	4.08	3.75
O12M-64_content	Old	Male	12	64	3.33	3.75	3.07	3.85	2.62
O15M-69_content	Old	Male	15	69	3.79	4.00	3.87	4.15	3.14
O17M-69_content	Old	Male	17	69	4.12	4.63	3.73	4.38	3.69
O20M-65_content	Old	Male	20	65	3.83	4.06	3.80	4.00	3.50
O21M-65_content	Old	Male	21	65	3.67	4.06	3.20	4.00	3.38
O35M-66_content	Old	Male	35	66	3.41	3.94	3.27	3.77	2.64
O42M-75_content	Old	Male	42	75	4.29	4.50	4.47	4.14	4.00
O50M-65_content	Old	Male	50	65	3.28	3.75	3.07	3.85	2.38
O55M-64_content	Old	Male	55	64	3.80	4.19	3.67	4.08	3.31
O58M-64_content	Old	Male	58	64	3.77	4.31	3.33	4.23	3.15
O59M-65_content	Old	Male	59	65	4.00	3.88	4.33	4.07	3.69
O63M-61_content	Old	Male	63	61	3.49	3.88	3.27	3.77	3.00
O64M-65_content	Old	Male	64	65	3.58	4.31	3.20	4.08	2.62
O65M-65_content	Old	Male	65	65	3.91	4.38	3.60	4.08	3.54
O66M-70_content	Old	Male	66	70	3.69	4.06	3.13	4.15	3.43
O67M-61_content	Old	Male	67	61	3.86	4.06	3.93	4.14	3.23
O68M-60_content	Old	Male	68	60	3.19	3.69	3.00	3.62	2.43
O69M-62_content	Old	Male	69	62	3.84	3.94	3.93	4.14	3.31
O70M-66_content	Old	Male	70	66	3.17	3.63	3.00	3.57	2.38
O71M-65_content	Old	Male	71	65	4.21	4.50	4.27	4.31	3.69

Image Information					Perceived emotional intensity (1=lowerst, 5=highest)				
Image Name	Model Age group	Model Gender	Model Code	Actual Age (Years)	All Raters	Older Female raters	Young Female raters	Older Male raters	Younger Male raters
Y3F-20_neutral	Young	Female	3	20	3.31	3.38	3.53	3.69	2.64
Y4F-19_neutral	Young	Female	4	19	3.38	3.69	3.13	3.62	3.13
Y5F-24_neutral	Young	Female	5	24	3.42	3.63	3.33	3.54	3.19
Y6F-23_neutral	Young	Female	6	23	3.59	3.63	3.60	3.86	3.23
Y12F-18_neutral	Young	Female	12	18	3.35	3.56	2.87	3.77	3.23
Y13F-20_neutral	Young	Female	13	20	3.22	3.19	3.00	3.77	3.00
Y14F-21_neutral	Young	Female	14	21	3.41	3.44	3.40	3.92	2.93
Y17F-33_neutral	Young	Female	17	33	3.43	3.81	3.40	3.77	2.81
Y18F-18_neutral	Young	Female	18	18	3.42	3.94	2.67	3.92	3.15
Y19F-19_neutral	Young	Female	19	19	3.26	3.25	3.33	3.23	3.21
Y22F-30_neutral	Young	Female	22	30	3.52	3.81	3.40	3.50	3.31
Y23F-30_neutral	Young	Female	23	30	3.33	3.63	2.93	3.54	3.23
Y25F-33_neutral	Young	Female	25	33	3.42	3.56	3.27	3.69	3.19
Y26F-32_neutral	Young	Female	26	32	3.21	3.19	3.20	3.21	3.23
Y31F-30_neutral	Young	Female	31	30	3.21	3.63	2.93	3.62	2.64
Y32F-20_neutral	Young	Female	32	20	3.38	3.69	3.40	3.54	2.94
Y37F-32_neutral	Young	Female	37	32	3.22	3.25	3.27	3.36	3.00
Y38F-24_neutral	Young	Female	38	24	3.52	3.63	3.53	3.64	3.23
Y39F-25_neutral	Young	Female	39	25	3.47	3.63	3.67	3.62	3.00
Y40F-28_neutral	Young	Female	40	28	3.48	3.50	3.53	3.50	3.38
Y42F-20_neutral	Young	Female	42	20	3.38	3.44	3.33	3.64	3.08
Y48F-23_neutral	Young	Female	48	23	3.25	3.31	3.13	3.62	3.00
Y50F-24_neutral	Young	Female	50	24	3.19	3.38	3.27	3.46	2.64
Y51F-23_neutral	Young	Female	51	23	3.23	3.63	2.93	3.38	3.00
Y52F-21_neutral	Young	Female	52	21	3.33	3.81	2.93	3.38	3.15
Y59F-23_neutral	Young	Female	59	23	3.03	3.31	3.07	3.15	2.63
Y65F-21_neutral	Young	Female	65	21	3.38	3.44	3.40	3.62	3.07
Y66F-26_neutral	Young	Female	66	26	3.38	3.50	3.33	3.77	3.00
Y69F-28_neutral	Young	Female	69	28	3.27	3.31	3.13	3.62	3.06
Y71F-20_neutral	Young	Female	71	20	3.42	3.63	3.33	3.31	3.38
Y72F-25_neutral	Young	Female	72	25	3.42	3.44	3.47	3.77	3.06
Y1M-19_neutral	Young	Male	1	19	3.50	3.44	3.40	3.92	3.29
Y2M-21_neutral	Young	Male	2	21	3.37	3.63	2.93	3.62	3.31
Y8M-27_neutral	Young	Male	8	27	3.36	3.44	3.40	3.69	2.93
Y10M-22_neutral	Young	Male	10	22	3.48	3.75	3.20	3.69	3.29
Y11M-20_neutral	Young	Male	11	20	3.45	3.75	3.07	3.69	3.31
Y15M-20_neutral	Young	Male	15	20	3.45	3.44	3.33	3.85	3.25
Y16M-21_neutral	Young	Male	16	21	3.36	3.75	3.13	3.50	3.00
Y21M-21_neutral	Young	Male	21	21	3.47	3.31	3.47	3.77	3.38
Y24M-19_neutral	Young	Male	24	19	3.48	3.56	3.20	3.62	3.56
Y27M-23_neutral	Young	Male	27	23	3.31	3.56	3.27	3.29	3.08
Y28M-20_neutral	Young	Male	28	20	3.26	3.44	3.27	3.54	2.79
Y29M-21_neutral	Young	Male	29	21	3.21	3.50	2.67	3.23	3.46
Y30M-25_neutral	Young	Male	30	25	3.22	3.31	3.33	3.23	3.00
Y33M-25_neutral	Young	Male	33	25	3.43	3.63	3.27	3.64	3.15
Y35M-20_neutral	Young	Male	35	20	2.95	2.94	3.00	3.14	2.69
Y36M-30_neutral	Young	Male	36	30	3.40	3.56	3.13	3.54	3.36
Y41M-19_neutral	Young	Male	41	19	3.50	3.88	3.20	3.77	3.19
Y44M-26_neutral	Young	Male	44	26	3.18	3.44	3.13	3.38	2.81
Y46M-18_neutral	Young	Male	46	18	3.28	3.44	3.00	3.62	3.08
Y47M-23_neutral	Young	Male	47	23	3.36	3.19	3.47	3.69	3.14
Y49M-23_neutral	Young	Male	49	23	3.21	3.56	3.13	3.46	2.64
Y53M-23_neutral	Young	Male	53	23	3.14	3.06	3.07	3.64	2.77
Y54M-26_neutral	Young	Male	54	26	3.29	3.38	3.33	3.38	3.07
Y55M-24_neutral	Young	Male	55	24	3.41	3.69	3.20	3.79	2.92
Y56M-24_neutral	Young	Male	56	24	3.21	3.25	3.20	3.62	2.79
Y57M-23_neutral	Young	Male	57	23	3.33	3.63	3.13	3.62	2.93
Y58M-22_neutral	Young	Male	58	22	3.18	3.50	2.73	3.69	2.77
Y60M-24_neutral	Young	Male	60	24	3.26	3.50	3.27	3.29	2.92
Y67M-19_neutral	Young	Male	67	19	3.07	3.63	2.67	3.29	2.62
Y68M-30_neutral	Young	Male	68	30	3.25	3.56	2.80	3.62	3.00
Y74M-31_neutral	Young	Male	74	31	3.23	3.81	2.53	3.62	2.92
Y75M-30_neutral	Young	Male	75	30	3.26	3.56	2.93	3.46	3.08
O4F-76_neutral	Old	Female	4	76	3.50	3.69	3.40	3.85	3.13
O7F-65_neutral	Old	Female	7	65	3.12	3.31	2.93	3.21	3.00
O9F-64_neutral	Old	Female	9	64	3.31	3.56	2.87	3.50	3.31
O10F-60_neutral	Old	Female	10	60	3.24	3.56	3.07	3.29	3.00
O16F-64_neutral	Old	Female	16	64	3.45	3.69	3.40	3.43	3.23
O19F-60_neutral	Old	Female	19	60	3.10	3.13	2.60	3.57	3.15
O22F-61_neutral	Old	Female	22	61	3.38	3.56	3.53	3.69	2.81
O23F-66_neutral	Old	Female	23	66	3.16	3.50	2.80	3.21	3.08
O24F-62_neutral	Old	Female	24	62	3.12	3.44	2.73	3.38	2.92
O26F-64_neutral	Old	Female	26	64	3.37	3.75	2.87	3.31	3.54
O27F-65_neutral	Old	Female	27	65	3.19	3.25	2.93	3.57	3.00
O28F-64_neutral	Old	Female	28	64	2.98	3.56	2.33	3.62	2.38
O29F-63_neutral	Old	Female	29	63	3.38	3.25	3.33	3.79	3.15
O34F-65_neutral	Old	Female	34	65	3.23	3.56	2.60	3.62	3.15
O38F-65_neutral	Old	Female	38	65	3.12	3.75	2.80	2.92	2.92
O40F-61_neutral	Old	Female	40	61	3.04	3.38	2.53	3.54	2.69
O41F-72_neutral	Old	Female	41	72	3.43	3.31	3.67	3.71	3.00
O43F-62_neutral	Old	Female	43	62	3.38	3.44	3.33	3.36	3.38
O45F-65_neutral	Old	Female	45	65	3.26	3.31	3.07	3.77	2.93
O47F-60_neutral	Old	Female	47	60	3.33	3.69	3.40	3.00	3.15
O48F-65_neutral	Old	Female	48	65	3.33	3.75	2.73	3.46	3.38
O49F-65_neutral	Old	Female	49	65	3.33	3.31	3.07	3.85	3.19
O51F-60_neutral	Old	Female	51	60	3.33	3.31	3.27	3.64	3.08
O52F-62_neutral	Old	Female	52	62	2.97	2.81	3.13	3.08	2.86
O53F-64_neutral	Old	Female	53	64	3.17	3.44	2.67	3.46	3.13
O56F-65_neutral	Old	Female	56	65	3.25	3.50	3.00	3.69	2.88
O8M-65_neutral	Old	Male	8	65	3.30	3.44	3.00	3.62	3.19
O12M-64_neutral	Old	Male	12	64	3.21	3.44	2.93	3.85	2.62
O15M-69_neutral	Old	Male	15	69	3.52	3.75	3.07	4.00	3.31
O17M-69_neutral	Old	Male	17	69	3.22	3.69	2.87	3.54	2.81
O20M-65_neutral	Old	Male	20	65	3.14	3.38	2.87	3.08	3.23
O21M-65_neutral	Old	Male	21	65	3.33	3.44	3.27	3.69	2.93
O35M-66_neutral	Old	Male	35	66	3.26	3.56	2.87	3.77	2.85
O42M-75_neutral	Old	Male	42	75	3.13	3.31	2.87	3.46	2.94
O50M-65_neutral	Old	Male	50	65	3.53	3.44	3.53	3.86	3.31
O55M-64_neutral	Old	Male	55	64	3.02	3.00	2.87	3.14	3.08
O58M-64_neutral	Old	Male	58	64	3.20	3.75	2.87	3.54	2.69
O59M-65_neutral	Old	Male	59	65	3.26	3.56	2.93	3.38	3.15
O63M-61_neutral	Old	Male	63	61	2.74	2.94	2.67	3.08	2.29
O64M-65_neutral	Old	Male	64	65	3.42	3.88	2.93	3.69	3.19
O65M-65_neutral	Old	Male	65	65	3.00	3.44	2.67	3.31	2.54
O66M-70_neutral	Old	Male	66	70	3.26	3.38	3.07	3.54	3.07
O67M-61_neutral	Old	Male	67	61	3.48	3.75	3.27	3.54	3.36
O68M-60_neutral	Old	Male	68	60	3.21	3.50	3.07	3.64	2.54
O69M-62_neutral	Old	Male	69	62	3.40	3.50	3.33	3.77	3.00
O70M-66_neutral	Old	Male	70	66	3.24	3.31	3.00	3.57	3.08
O71M-65_neutral	Old	Male	71	65	3.09	3.56	2.60	3.31	2.85

Image Information					Perceived emotional intensity (1=lowerst, 5=highest)				
Image Name	Model Age group	Model Gender	Model Code	Actual Age (Years)	All Raters	Older Female raters	Young Female raters	Older Male raters	Younger Male raters
Y3F-20_sad	Young	Female	3	20	4.10	4.25	4.00	4.23	3.94
Y4F-19_sad	Young	Female	4	19	3.17	3.06	3.40	3.08	3.13
Y5F-24_sad	Young	Female	5	24	4.00	4.13	4.20	3.92	3.69
Y6F-23_sad	Young	Female	6	23	3.04	2.94	3.00	3.23	3.00
Y12F-18_sad	Young	Female	12	18	2.71	3.06	2.67	2.69	2.36
Y13F-20_sad	Young	Female	13	20	3.60	3.44	3.93	3.79	3.23
Y14F-21_sad	Young	Female	14	21	3.37	3.44	3.33	3.54	3.19
Y17F-33_sad	Young	Female	17	33	3.70	3.69	4.07	3.46	3.56
Y18F-18_sad	Young	Female	18	18	2.95	3.13	3.27	3.07	2.23
Y19F-19_sad	Young	Female	19	19	2.82	3.00	2.73	3.00	2.54
Y22F-30_sad	Young	Female	22	30	3.28	3.31	3.53	3.57	2.62
Y23F-30_sad	Young	Female	23	30	3.70	3.69	4.07	3.77	3.23
Y25F-33_sad	Young	Female	25	33	3.96	3.75	4.27	4.08	3.77
Y26F-32_sad	Young	Female	26	32	3.19	3.38	3.27	3.46	2.64
Y31F-30_sad	Young	Female	31	30	3.00	3.13	3.10	3.42	2.36
Y32F-20_sad	Young	Female	32	20	3.14	3.00	3.33	3.54	2.69
Y37F-32_sad	Young	Female	37	32	2.53	2.69	2.60	2.62	2.25
Y38F-24_sad	Young	Female	38	24	3.21	3.38	3.33	3.36	2.69
Y39F-25_sad	Young	Female	39	25	3.67	3.50	4.00	3.85	3.36
Y40F-28_sad	Young	Female	40	28	3.05	3.44	2.87	3.29	2.54
Y42F-20_sad	Young	Female	42	20	3.40	3.31	3.67	3.54	3.07
Y48F-23_sad	Young	Female	48	23	3.58	3.75	3.47	3.92	3.25
Y50F-24_sad	Young	Female	50	24	2.74	3.06	2.20	3.31	2.38
Y51F-23_sad	Young	Female	51	23	3.54	3.94	3.33	3.85	3.00
Y52F-21_sad	Young	Female	52	21	3.12	3.44	3.20	3.38	2.43
Y59F-23_sad	Young	Female	59	23	3.32	3.25	3.53	3.31	3.15
Y65F-21_sad	Young	Female	65	21	2.91	2.75	3.27	3.38	2.29
Y66F-26_sad	Young	Female	66	26	2.56	2.63	2.53	2.85	2.23
Y69F-28_sad	Young	Female	69	28	3.90	3.69	4.20	4.00	3.69
Y71F-20_sad	Young	Female	71	20	3.09	3.31	3.20	3.38	2.43
Y72F-25_sad	Young	Female	72	25	2.95	3.13	2.80	3.46	2.50
Y1M-19_sad	Young	Male	1	19	3.10	3.25	3.33	2.85	2.94
Y2M-21_sad	Young	Male	2	21	2.93	2.94	3.27	3.15	2.44
Y8M-27_sad	Young	Male	8	27	3.00	3.19	3.13	3.00	2.62
Y10M-22_sad	Young	Male	10	22	3.19	3.44	3.07	3.54	2.69
Y11M-20_sad	Young	Male	11	20	3.87	4.00	4.00	4.08	3.44
Y15M-20_sad	Young	Male	15	20	2.66	2.94	2.80	3.00	1.86
Y16M-21_sad	Young	Male	16	21	3.45	3.25	3.67	4.15	2.88
Y21M-21_sad	Young	Male	21	21	2.75	2.75	3.00	3.15	2.19
Y24M-19_sad	Young	Male	24	19	3.19	3.19	3.53	3.36	2.62
Y27M-23_sad	Young	Male	27	23	3.49	3.50	3.47	3.69	3.31
Y28M-20_sad	Young	Male	28	20	3.72	3.81	3.93	3.85	3.23
Y29M-21_sad	Young	Male	29	21	3.63	3.81	3.47	3.62	3.62
Y30M-25_sad	Young	Male	30	25	3.53	3.69	3.47	3.92	3.13
Y33M-25_sad	Young	Male	33	25	4.12	4.19	4.27	4.23	3.77
Y35M-20_sad	Young	Male	35	20	3.23	3.50	3.20	3.46	2.81
Y36M-30_sad	Young	Male	36	30	3.16	3.31	3.00	3.50	2.77
Y41M-19_sad	Young	Male	41	19	3.58	3.75	3.53	3.85	3.15
Y44M-26_sad	Young	Male	44	26	3.83	3.63	4.07	4.00	3.62
Y46M-18_sad	Young	Male	46	18	2.93	3.25	2.67	3.08	2.69
Y47M-23_sad	Young	Male	47	23	3.93	3.81	4.07	4.15	3.75
Y49M-23_sad	Young	Male	49	23	3.89	3.88	4.33	4.15	3.15
Y53M-23_sad	Young	Male	53	23	2.63	3.13	2.27	3.08	2.00
Y54M-26_sad	Young	Male	54	26	2.98	3.31	2.80	3.50	2.23
Y55M-24_sad	Young	Male	55	24	3.20	3.44	3.40	3.08	2.88
Y56M-24_sad	Young	Male	56	24	3.48	3.50	3.93	3.36	3.08
Y57M-23_sad	Young	Male	57	23	3.15	3.19	3.40	3.15	2.88
Y58M-22_sad	Young	Male	58	22	3.19	3.28	3.23	3.38	2.86
Y60M-24_sad	Young	Male	60	24	3.95	4.31	3.93	4.00	3.46
Y67M-19_sad	Young	Male	67	19	2.95	2.94	3.20	3.23	2.50
Y68M-30_sad	Young	Male	68	30	3.65	3.31	3.87	3.85	3.63
Y74M-31_sad	Young	Male	74	31	3.00	3.31	3.27	3.23	2.08
Y75M-30_sad	Young	Male	75	30	2.90	3.00	2.80	3.31	2.56
O4F-76_sad	Old	Female	4	76	2.93	2.81	3.47	3.08	2.36
O7F-65_sad	Old	Female	7	65	4.41	4.56	4.47	4.46	4.14
O9F-64_sad	Old	Female	9	64	2.82	3.06	2.73	3.46	2.00
O10F-60_sad	Old	Female	10	60	2.63	2.88	2.73	2.69	2.25
O16F-64_sad	Old	Female	16	64	3.39	3.38	3.27	3.85	3.08
O19F-60_sad	Old	Female	19	60	3.89	3.75	4.27	4.08	3.46
O22F-61_sad	Old	Female	22	61	3.33	3.38	3.20	3.92	2.86
O23F-66_sad	Old	Female	23	66	3.28	3.63	2.93	3.69	2.85
O24F-62_sad	Old	Female	24	62	3.97	3.94	4.07	4.15	3.75
O26F-64_sad	Old	Female	26	64	3.78	3.56	4.20	3.92	3.43
O27F-65_sad	Old	Female	27	65	3.24	3.25	3.53	3.77	2.43
O28F-64_sad	Old	Female	28	64	3.07	3.06	3.20	2.85	3.13
O29F-63_sad	Old	Female	29	63	3.78	3.63	4.33	3.46	3.64
O34F-65_sad	Old	Female	34	65	2.79	2.94	2.47	3.43	2.31
O38F-65_sad	Old	Female	38	65	3.08	3.25	3.33	3.15	2.63
O40F-61_sad	Old	Female	40	61	3.23	3.19	3.20	3.77	2.77
O41F-72_sad	Old	Female	41	72	4.02	3.88	4.13	4.14	3.92
O43F-62_sad	Old	Female	43	62	2.83	2.63	3.13	3.00	2.57
O45F-65_sad	Old	Female	45	65	3.25	3.50	3.13	3.69	2.62
O47F-60_sad	Old	Female	47	60	2.95	3.19	3.13	3.31	2.14
O48F-65_sad	Old	Female	48	65	2.75	3.06	2.27	3.23	2.46
O49F-65_sad	Old	Female	49	65	3.52	3.81	3.53	3.85	2.86
O51F-60_sad	Old	Female	51	60	4.02	4.00	4.13	4.15	3.79
O52F-62_sad	Old	Female	52	62	3.76	3.81	3.67	3.86	3.69
O53F-64_sad	Old	Female	53	64	4.12	4.44	3.93	4.29	3.77
O56F-65_sad	Old	Female	56	65	2.84	2.88	2.87	3.38	2.29
O8M-65_sad	Old	Male	8	65	2.87	3.00	2.93	3.08	2.50
O12M-64_sad	Old	Male	12	64	4.36	4.31	4.67	4.15	4.29
O15M-69_sad	Old	Male	15	69	3.41	3.44	3.20	4.14	2.85
O17M-69_sad	Old	Male	17	69	3.21	3.06	3.53	3.46	2.79
O20M-65_sad	Old	Male	20	65	3.22	2.94	3.40	3.08	3.44
O21M-65_sad	Old	Male	21	65	3.64	3.75	3.60	4.29	2.85
O35M-66_sad	Old	Male	35	66	3.79	3.63	4.00	4.07	3.46
O42M-75_sad	Old	Male	42	75	3.97	4.25	3.73	4.46	3.50
O50M-65_sad	Old	Male	50	65	3.59	3.56	3.40	3.79	3.62
O55M-64_sad	Old	Male	55	64	4.05	4.00	4.00	4.15	4.07
O58M-64_sad	Old	Male	58	64	3.47	3.50	3.73	3.69	2.93
O59M-65_sad	Old	Male	59	65	3.07	3.25	2.87	3.54	2.62
O63M-61_sad	Old	Male	63	61	4.00	3.94	4.20	3.92	3.94
O64M-65_sad	Old	Male	64	65	3.62	3.63	3.67	3.64	3.54
O65M-65_sad	Old	Male	65	65	3.33	3.38	3.40	3.85	2.71
O66M-70_sad	Old	Male	66	70	2.93	2.94	3.20	3.15	2.43
O67M-61_sad	Old	Male	67	61	3.59	3.38	3.93	3.77	3.29
O68M-60_sad	Old	Male	68	60	3.91	3.94	4.07	4.00	3.64
O69M-62_sad	Old	Male	69	62	2.74	3.25	2.33	3.23	2.08
O70M-66_sad	Old	Male	70	66	2.86	2.94	3.33	3.00	2.14
O71M-65_sad	Old	Male	71	65	3.33	3.13	3.67	3.38	3.19

Image Information					Perceived emotional intensity (1=lowerst, 5=highest)				
Image Name	Model Age group	Model Gender	Model Code	Actual Age (Years)	All Raters	Older Female raters	Young Female raters	Older Male raters	Younger Male raters
Y3F-20_anger	Young	Female	3	20	3.52	3.56	3.60	4.00	2.85
Y4F-19_anger	Young	Female	4	19	3.30	3.56	3.07	3.46	3.08
Y5F-24_anger	Young	Female	5	24	2.90	2.75	3.00	3.31	2.57
Y6F-23_anger	Young	Female	6	23	3.45	3.81	3.33	3.69	2.93
Y12F-18_anger	Young	Female	12	18	4.21	4.56	4.20	4.57	3.38
Y13F-20_anger	Young	Female	13	20	3.53	3.63	3.60	4.23	2.71
Y14F-21_anger	Young	Female	14	21	3.70	3.75	3.60	4.15	3.38
Y17F-33_anger	Young	Female	17	33	3.50	3.69	3.53	3.85	3.00
Y18F-18_anger	Young	Female	18	18	2.47	2.50	2.33	2.86	2.15
Y19F-19_anger	Young	Female	19	19	3.12	3.38	3.07	3.77	2.23
Y22F-30_anger	Young	Female	22	30	3.38	3.50	3.53	3.71	2.69
Y23F-30_anger	Young	Female	23	30	4.48	4.44	4.47	4.92	4.19
Y25F-33_anger	Young	Female	25	33	3.79	4.00	3.73	3.92	3.46
Y26F-32_anger	Young	Female	26	32	3.57	3.81	3.33	3.77	3.38
Y31F-30_anger	Young	Female	31	30	3.98	4.44	3.80	4.31	3.31
Y32F-20_anger	Young	Female	32	20	3.12	3.56	3.13	3.36	2.31
Y37F-32_anger	Young	Female	37	32	3.14	3.44	2.60	3.86	2.62
Y38F-24_anger	Young	Female	38	24	3.43	3.81	3.20	3.71	2.92
Y39F-25_anger	Young	Female	39	25	3.29	3.56	2.80	3.93	2.85
Y40F-28_anger	Young	Female	40	28	2.80	3.16	2.60	2.96	2.42
Y42F-20_anger	Young	Female	42	20	3.50	3.63	3.60	3.79	2.92
Y48F-23_anger	Young	Female	48	23	3.77	3.69	3.53	4.31	3.63
Y50F-24_anger	Young	Female	50	24	3.09	3.31	3.00	3.54	2.50
Y51F-23_anger	Young	Female	51	23	2.98	3.38	2.73	3.54	2.38
Y52F-21_anger	Young	Female	52	21	3.91	4.25	3.73	4.38	3.29
Y59F-23_anger	Young	Female	59	23	3.28	3.75	3.00	3.86	2.38
Y65F-21_anger	Young	Female	65	21	3.69	3.69	3.60	4.07	3.38
Y66F-26_anger	Young	Female	66	26	3.50	3.50	3.33	3.69	3.50
Y69F-28_anger	Young	Female	69	28	3.55	3.75	3.33	4.00	3.19
Y71F-20_anger	Young	Female	71	20	3.34	3.94	3.00	3.93	2.38
Y72F-25_anger	Young	Female	72	25	3.42	3.44	3.40	3.92	3.00
Y1M-19_anger	Young	Male	1	19	4.23	4.50	4.07	4.46	3.85
Y2M-21_anger	Young	Male	2	21	3.98	4.25	3.60	4.46	3.62
Y8M-27_anger	Young	Male	8	27	2.80	3.00	2.67	3.31	2.31
Y10M-22_anger	Young	Male	10	22	2.72	3.19	2.40	3.15	2.19
Y11M-20_anger	Young	Male	11	20	4.05	4.06	4.00	4.38	3.77
Y15M-20_anger	Young	Male	15	20	4.00	4.13	3.53	4.62	3.77
Y16M-21_anger	Young	Male	16	21	4.33	4.56	4.27	4.54	4.00
Y21M-21_anger	Young	Male	21	21	4.23	4.31	4.20	4.46	3.92
Y24M-19_anger	Young	Male	24	19	3.43	3.81	3.33	3.77	2.79
Y27M-23_anger	Young	Male	27	23	3.48	3.75	3.33	3.71	3.08
Y28M-20_anger	Young	Male	28	20	3.07	3.44	2.87	3.46	2.50
Y29M-21_anger	Young	Male	29	21	4.05	4.25	3.67	4.38	3.94
Y30M-25_anger	Young	Male	30	25	3.84	4.00	3.47	4.36	3.54
Y33M-25_anger	Young	Male	33	25	3.58	3.94	3.47	4.00	2.85
Y35M-20_anger	Young	Male	35	20	3.50	3.81	3.33	3.86	2.92
Y36M-30_anger	Young	Male	36	30	3.46	3.84	3.67	3.73	2.54
Y41M-19_anger	Young	Male	41	19	2.86	3.38	2.53	3.50	1.92
Y44M-26_anger	Young	Male	44	26	3.32	3.63	2.93	4.00	2.69
Y46M-18_anger	Young	Male	46	18	3.73	4.13	3.47	4.23	3.19
Y47M-23_anger	Young	Male	47	23	3.45	3.75	3.27	3.71	3.00
Y49M-23_anger	Young	Male	49	23	3.43	3.56	3.47	4.00	2.71
Y53M-23_anger	Young	Male	53	23	3.33	3.69	3.20	3.92	2.50
Y54M-26_anger	Young	Male	54	26	3.24	3.50	2.93	3.77	2.79
Y55M-24_anger	Young	Male	55	24	4.72	4.63	4.93	4.69	4.62
Y56M-24_anger	Young	Male	56	24	2.86	2.75	3.00	3.23	2.50
Y57M-23_anger	Young	Male	57	23	3.46	3.63	3.40	3.92	2.85
Y58M-22_anger	Young	Male	58	22	3.09	3.19	2.87	3.93	2.31
Y60M-24_anger	Young	Male	60	24	2.93	3.25	2.73	3.71	1.92
Y67M-19_anger	Young	Male	67	19	3.47	3.81	3.53	3.54	2.93
Y68M-30_anger	Young	Male	68	30	3.45	3.56	3.53	3.85	2.94
Y74M-31_anger	Young	Male	74	31	3.18	3.28	3.00	3.86	2.54
Y75M-30_anger	Young	Male	75	30	3.13	3.53	2.63	3.70	2.63
O4F-76_anger	Old	Female	4	76	3.32	3.63	3.33	3.69	2.54
O7F-65_anger	Old	Female	7	65	3.68	3.69	3.80	4.00	3.31
O9F-64_anger	Old	Female	9	64	3.25	3.38	3.13	3.92	2.54
O10F-60_anger	Old	Female	10	60	3.28	3.50	3.27	3.46	2.85
O16F-64_anger	Old	Female	16	64	2.82	3.38	2.33	3.15	2.44
O19F-60_anger	Old	Female	19	60	4.27	4.56	3.93	4.77	3.88
O22F-61_anger	Old	Female	22	61	3.48	4.00	3.27	4.21	2.31
O23F-66_anger	Old	Female	23	66	3.13	3.38	3.13	3.62	2.50
O24F-62_anger	Old	Female	24	62	3.85	4.13	3.80	4.08	3.44
O26F-64_anger	Old	Female	26	64	4.10	4.31	3.93	4.54	3.69
O27F-65_anger	Old	Female	27	65	3.17	3.38	3.47	3.46	2.44
O28F-64_anger	Old	Female	28	64	3.72	4.19	3.60	4.08	3.00
O29F-63_anger	Old	Female	29	63	3.40	3.31	3.60	3.77	3.00
O34F-65_anger	Old	Female	34	65	2.96	3.13	2.80	3.15	2.77
O38F-65_anger	Old	Female	38	65	3.07	3.13	2.80	3.54	2.85
O40F-61_anger	Old	Female	40	61	3.15	3.19	3.00	3.85	2.69
O41F-72_anger	Old	Female	41	72	3.22	3.38	3.27	3.38	2.88
O43F-62_anger	Old	Female	43	62	3.29	3.75	3.07	3.62	2.71
O45F-65_anger	Old	Female	45	65	3.63	3.50	3.47	4.15	3.46
O47F-60_anger	Old	Female	47	60	3.86	4.00	3.93	4.15	3.31
O48F-65_anger	Old	Female	48	65	4.32	4.56	4.20	4.77	3.69
O49F-65_anger	Old	Female	49	65	4.02	4.25	4.07	4.46	3.38
O51F-60_anger	Old	Female	51	60	3.78	3.88	3.67	4.31	3.38
O52F-62_anger	Old	Female	52	62	3.57	3.69	3.47	4.21	2.85
O53F-64_anger	Old	Female	53	64	3.32	3.50	3.53	3.77	2.56
O56F-65_anger	Old	Female	56	65	3.10	3.25	3.33	3.46	2.36
O8M-65_anger	Old	Male	8	65	3.72	3.97	3.90	4.08	2.93
O12M-64_anger	Old	Male	12	64	4.02	4.06	3.93	4.29	3.77
O15M-69_anger	Old	Male	15	69	4.42	4.56	4.33	4.77	4.06
O17M-69_anger	Old	Male	17	69	3.95	4.25	3.67	4.46	3.38
O20M-65_anger	Old	Male	20	65	3.82	3.88	3.60	4.38	3.50
O21M-65_anger	Old	Male	21	65	3.43	3.63	3.47	3.85	2.88
O35M-66_anger	Old	Male	35	66	3.05	3.13	2.87	3.64	2.54
O42M-75_anger	Old	Male	42	75	3.98	4.31	3.60	4.50	3.46
O50M-65_anger	Old	Male	50	65	3.89	4.44	3.40	4.54	3.15
O55M-64_anger	Old	Male	55	64	3.70	4.00	3.53	4.23	3.13
O58M-64_anger	Old	Male	58	64	4.02	4.22	3.93	4.35	3.59
O59M-65_anger	Old	Male	59	65	3.45	3.56	3.63	4.00	2.61
O63M-61_anger	Old	Male	63	61	2.84	2.75	3.00	3.38	2.29
O64M-65_anger	Old	Male	64	65	3.48	3.38	3.53	3.92	3.19
O65M-65_anger	Old	Male	65	65	3.14	3.13	3.33	3.62	2.50
O66M-70_anger	Old	Male	66	70	3.81	4.13	3.53	4.15	3.38
O67M-61_anger	Old	Male	67	61	3.45	3.81	3.73	3.79	2.31
O68M-60_anger	Old	Male	68	60	3.72	3.69	3.73	3.92	3.54
O69M-62_anger	Old	Male	69	62	3.19	3.25	3.33	3.50	2.62
O70M-66_anger	Old	Male	70	66	3.28	3.53	3.17	3.74	2.67
O71M-65_anger	Old	Male	71	65	3.79	4.00	3.87	4.15	3.14

Image Information					Perceived emotional intensity (1=lowerst, 5=highest)				
Image Name	Model Age group	Model Gender	Model Code	Actual Age (Years)	All Raters	Older Female raters	Young Female raters	Older Male raters	Younger Male raters
Y3F-20_fear	Young	Female	3	20	3.74	3.88	3.93	4.14	2.92
Y4F-19_fear	Young	Female	4	19	4.05	4.25	4.07	4.21	3.62
Y5F-24_fear	Young	Female	5	24	3.49	3.81	3.53	3.77	2.77
Y6F-23_fear	Young	Female	6	23	3.82	3.81	4.07	4.15	3.31
Y12F-18_fear	Young	Female	12	18	2.81	3.06	2.53	3.15	2.50
Y13F-20_fear	Young	Female	13	20	3.60	3.56	3.87	3.77	3.25
Y14F-21_fear	Young	Female	14	21	3.68	4.00	3.47	4.00	3.23
Y17F-33_fear	Young	Female	17	33	4.48	4.75	4.47	4.62	4.13
Y18F-18_fear	Young	Female	18	18	4.41	4.63	4.20	4.64	4.15
Y19F-19_fear	Young	Female	19	19	2.98	3.13	2.93	3.69	2.21
Y22F-30_fear	Young	Female	22	30	4.26	4.44	4.20	4.31	4.08
Y23F-30_fear	Young	Female	23	30	4.28	4.56	4.33	4.31	3.86
Y25F-33_fear	Young	Female	25	33	4.43	4.44	4.53	4.57	4.15
Y26F-32_fear	Young	Female	26	32	4.14	4.25	3.93	4.46	3.92
Y31F-30_fear	Young	Female	31	30	4.26	4.44	4.07	4.54	4.00
Y32F-20_fear	Young	Female	32	20	3.98	4.19	4.27	3.85	3.63
Y37F-32_fear	Young	Female	37	32	4.26	4.38	4.40	4.57	3.62
Y38F-24_fear	Young	Female	38	24	4.22	4.38	4.47	4.15	3.86
Y39F-25_fear	Young	Female	39	25	4.55	4.31	4.80	4.77	4.38
Y40F-28_fear	Young	Female	40	28	4.52	4.69	4.40	4.62	4.36
Y42F-20_fear	Young	Female	42	20	4.22	4.63	4.07	4.50	3.62
Y48F-23_fear	Young	Female	48	23	4.38	4.56	4.40	4.54	4.00
Y50F-24_fear	Young	Female	50	24	4.05	4.19	3.80	4.38	3.85
Y51F-23_fear	Young	Female	51	23	3.63	3.94	3.40	4.08	3.08
Y52F-21_fear	Young	Female	52	21	4.52	4.44	4.53	4.85	4.29
Y59F-23_fear	Young	Female	59	23	4.22	4.25	4.40	4.54	3.75
Y65F-21_fear	Young	Female	65	21	4.19	4.25	4.53	4.43	3.46
Y66F-26_fear	Young	Female	66	26	4.00	4.19	4.27	4.00	3.50
Y69F-28_fear	Young	Female	69	28	3.37	3.44	3.40	3.62	3.00
Y71F-20_fear	Young	Female	71	20	3.65	3.56	3.27	4.15	3.69
Y72F-25_fear	Young	Female	72	25	3.18	3.69	2.87	3.62	2.46
Y1M-19_fear	Young	Male	1	19	3.58	3.81	3.53	3.62	3.38
Y2M-21_fear	Young	Male	2	21	2.97	3.00	3.67	2.62	2.50
Y8M-27_fear	Young	Male	8	27	4.05	4.06	4.33	4.38	3.43
Y10M-22_fear	Young	Male	10	22	3.85	3.94	3.80	4.00	3.69
Y11M-20_fear	Young	Male	11	20	4.05	4.03	4.10	4.23	3.85
Y15M-20_fear	Young	Male	15	20	3.79	4.00	3.80	3.85	3.46
Y16M-21_fear	Young	Male	16	21	4.22	4.16	4.37	4.48	3.90
Y21M-21_fear	Young	Male	21	21	3.80	4.13	3.87	4.31	3.00
Y24M-19_fear	Young	Male	24	19	4.34	4.44	4.47	4.54	3.93
Y28M-20_fear	Young	Male	28	20	4.12	4.19	4.43	4.12	3.71
Y29M-21_fear	Young	Male	29	21	4.34	4.38	4.73	4.36	3.85
Y30M-25_fear	Young	Male	30	25	4.72	4.75	4.80	4.93	4.38
Y33M-25_fear	Young	Male	33	25	4.24	4.31	4.47	4.31	3.86
Y35M-20_fear	Young	Male	35	20	4.12	4.25	4.00	4.15	4.06
Y36M-30_fear	Young	Male	36	30	4.45	4.50	4.60	4.54	4.14
Y41M-19_fear	Young	Male	41	19	3.59	3.63	3.80	3.92	3.00
Y44M-26_fear	Young	Male	44	26	3.24	3.56	3.53	3.46	2.36
Y46M-18_fear	Young	Male	46	18	3.64	4.06	3.53	3.93	2.92
Y47M-23_fear	Young	Male	47	23	4.02	4.06	4.07	4.23	3.75
Y49M-23_fear	Young	Male	49	23	4.56	4.31	4.67	4.77	4.54
Y53M-23_fear	Young	Male	53	23	3.50	3.75	3.40	3.92	3.00
Y54M-26_fear	Young	Male	54	26	3.90	4.31	4.00	4.15	3.19
Y55M-24_fear	Young	Male	55	24	3.88	3.88	4.07	4.08	3.56
Y56M-24_fear	Young	Male	56	24	3.51	3.56	3.47	3.92	3.08
Y57M-23_fear	Young	Male	57	23	4.65	4.63	4.67	4.77	4.54
Y58M-22_fear	Young	Male	58	22	4.60	4.75	4.73	4.71	4.15
Y60M-24_fear	Young	Male	60	24	4.19	4.31	4.20	4.38	3.85
Y67M-19_fear	Young	Male	67	19	3.50	3.38	3.53	4.08	3.13
Y68M-30_fear	Young	Male	68	30	4.48	4.38	4.33	4.77	4.50
Y74M-31_fear	Young	Male	74	31	3.79	4.00	3.40	4.00	3.77
Y75M-30_fear	Young	Male	75	30	3.52	3.63	3.60	3.86	2.92
O4F-76_fear	Old	Female	4	76	4.21	4.13	4.27	4.37	4.08
O7F-65_fear	Old	Female	7	65	3.68	3.81	3.53	4.08	3.31
O9F-64_fear	Old	Female	9	64	4.17	4.31	4.27	4.38	3.71
O10F-60_fear	Old	Female	10	60	3.31	3.31	3.67	3.69	2.57
O16F-64_fear	Old	Female	16	64	4.31	4.06	4.73	4.46	4.00
O19F-60_fear	Old	Female	19	60	4.23	4.31	4.20	4.31	4.08
O22F-61_fear	Old	Female	22	61	3.34	3.63	3.47	3.85	2.43
O23F-66_fear	Old	Female	23	66	4.14	4.25	4.07	4.54	3.71
O24F-62_fear	Old	Female	24	62	4.48	4.56	4.67	4.71	3.92
O26F-64_fear	Old	Female	26	64	3.97	4.13	4.13	4.36	3.15
O27F-65_fear	Old	Female	27	65	2.93	3.06	2.73	3.43	2.46
O28F-64_fear	Old	Female	28	64	3.54	3.50	3.27	4.15	3.31
O29F-63_fear	Old	Female	29	63	4.28	4.44	4.40	4.54	3.71
O34F-65_fear	Old	Female	34	65	3.41	3.56	3.27	3.92	2.93
O38F-65_fear	Old	Female	38	65	3.28	3.19	3.27	3.77	2.93
O40F-61_fear	Old	Female	40	61	4.34	4.19	4.40	4.64	4.15
O41F-72_fear	Old	Female	41	72	4.38	4.69	4.20	4.57	4.00
O43F-62_fear	Old	Female	43	62	3.71	3.88	4.07	4.00	2.86
O45F-65_fear	Old	Female	45	65	3.83	3.88	4.00	3.93	3.46
O47F-60_fear	Old	Female	47	60	3.79	3.81	4.00	4.31	3.07
O48F-65_fear	Old	Female	48	65	4.03	4.13	4.40	4.00	3.63
O49F-65_fear	Old	Female	49	65	4.52	4.50	4.60	4.62	4.38
O51F-60_fear	Old	Female	51	60	3.78	4.19	3.67	4.38	2.86
O52F-62_fear	Old	Female	52	62	3.85	3.88	3.73	4.23	3.63
O53F-64_fear	Old	Female	53	64	3.76	3.81	3.93	4.00	3.29
O56F-65_fear	Old	Female	56	65	3.02	3.44	2.93	3.36	2.23
O8M-65_fear	Old	Male	8	65	3.98	3.88	4.07	4.15	3.86
O12M-64_fear	Old	Male	12	64	4.03	4.13	4.13	4.43	3.38
O15M-69_fear	Old	Male	15	69	4.37	4.31	4.53	4.69	4.00
O17M-69_fear	Old	Male	17	69	3.59	3.50	3.93	4.08	2.86
O20M-65_fear	Old	Male	20	65	3.19	3.06	3.47	3.79	2.38
O21M-65_fear	Old	Male	21	65	3.42	3.56	3.27	3.92	2.92
O35M-66_fear	Old	Male	35	66	2.97	3.25	3.00	3.31	2.29
O42M-75_fear	Old	Male	42	75	4.18	4.25	4.33	4.38	3.81
O50M-65_fear	Old	Male	50	65	4.65	4.69	4.53	4.77	4.62
O55M-64_fear	Old	Male	55	64	4.53	4.63	4.73	4.64	4.08
O58M-64_fear	Old	Male	58	64	4.67	4.69	4.80	4.85	4.38
O59M-65_fear	Old	Male	59	65	3.84	3.69	4.13	3.92	3.62
O63M-61_fear	Old	Male	63	61	4.28	4.06	4.20	4.69	4.23
O64M-65_fear	Old	Male	64	65	4.05	4.31	3.93	4.21	3.69
O65M-65_fear	Old	Male	65	65	2.86	2.88	2.53	3.57	2.46
O66M-70_fear	Old	Male	66	70	3.45	3.44	3.67	4.00	2.71
O67M-61_fear	Old	Male	67	61	4.09	4.31	4.13	4.36	3.46
O68M-60_fear	Old	Male	68	60	3.87	3.69	4.13	4.08	3.63
O69M-62_fear	Old	Male	69	62	4.05	4.19	4.33	4.15	3.56
O70M-66_fear	Old	Male	70	66	3.57	3.88	3.73	4.00	2.75
O71M-65_fear	Old	Male	71	65	4.10	4.25	4.13	4.36	3.62

Image Information					Perceived emotional intensity (1=lowest, 5=highest)				
Image Name	Model Age group	Model Gender	Model Code	Actual Age (Years)	All Raters	Older Female raters	Young Female raters	Older Male raters	Younger Male raters
Y3F-20_disgust	Young	Female	3	20	4.40	4.19	4.67	4.31	4.44
Y4F-19_disgust	Young	Female	4	19	4.07	4.00	4.40	4.08	3.81
Y5F-24_disgust	Young	Female	5	24	4.02	4.06	3.93	4.23	3.88
Y6F-23_disgust	Young	Female	6	23	3.88	4.00	3.87	3.92	3.69
Y12F-18_disgust	Young	Female	12	18	3.67	3.81	4.07	3.77	3.00
Y13F-20_disgust	Young	Female	13	20	3.60	3.63	3.93	3.64	3.15
Y14F-21_disgust	Young	Female	14	21	4.38	4.13	4.73	4.23	4.44
Y17F-33_disgust	Young	Female	17	33	4.43	4.50	4.67	4.38	4.19
Y18F-18_disgust	Young	Female	18	18	3.37	3.28	3.40	3.81	3.00
Y19F-19_disgust	Young	Female	19	19	3.30	3.19	3.33	3.46	3.23
Y22F-30_disgust	Young	Female	22	30	4.57	4.63	4.60	4.64	4.38
Y23F-30_disgust	Young	Female	23	30	4.62	4.56	4.73	4.69	4.50
Y25F-33_disgust	Young	Female	25	33	4.71	4.88	4.53	4.69	4.71
Y26F-32_disgust	Young	Female	26	32	4.28	4.44	4.20	4.15	4.31
Y31F-30_disgust	Young	Female	31	30	4.25	4.31	4.33	4.31	4.00
Y32F-20_disgust	Young	Female	32	20	3.77	3.88	3.67	4.00	3.54
Y37F-32_disgust	Young	Female	37	32	4.28	4.31	4.53	4.31	3.92
Y38F-24_disgust	Young	Female	38	24	3.52	3.75	3.87	3.57	2.77
Y39F-25_disgust	Young	Female	39	25	3.98	3.75	4.73	3.62	3.79
Y40F-28_disgust	Young	Female	40	28	3.57	3.56	3.53	3.77	3.43
Y42F-20_disgust	Young	Female	42	20	4.11	4.06	4.20	4.08	4.08
Y48F-23_disgust	Young	Female	48	23	4.40	4.38	4.73	4.46	4.00
Y50F-24_disgust	Young	Female	50	24	3.99	4.00	4.20	4.08	3.70
Y51F-23_disgust	Young	Female	51	23	4.02	4.19	4.07	4.08	3.69
Y52F-21_disgust	Young	Female	52	21	4.23	4.13	4.40	4.31	4.13
Y59F-23_disgust	Young	Female	59	23	4.40	4.44	4.47	4.31	4.36
Y65F-21_disgust	Young	Female	65	21	4.57	4.63	4.87	4.50	4.23
Y66F-26_disgust	Young	Female	66	26	4.60	4.63	4.67	4.64	4.46
Y69F-28_disgust	Young	Female	69	28	4.33	4.25	4.73	4.23	4.13
Y71F-20_disgust	Young	Female	71	20	3.78	4.00	3.77	4.15	3.21
Y72F-25_disgust	Young	Female	72	25	3.62	3.56	3.80	4.00	3.08
Y1M-19_disgust	Young	Male	1	19	4.50	4.31	4.67	4.69	4.36
Y2M-21_disgust	Young	Male	2	21	4.02	4.06	4.20	3.92	3.88
Y8M-27_disgust	Young	Male	8	27	3.92	3.94	3.93	4.00	3.81
Y10M-22_disgust	Young	Male	10	22	4.09	4.13	4.07	4.29	3.85
Y11M-20_disgust	Young	Male	11	20	3.57	3.69	3.93	3.64	2.92
Y15M-20_disgust	Young	Male	15	20	3.88	4.00	3.87	4.14	3.46
Y16M-21_disgust	Young	Male	16	21	4.47	4.56	4.67	4.50	4.08
Y21M-21_disgust	Young	Male	21	21	4.10	4.19	4.13	4.07	4.00
Y24M-19_disgust	Young	Male	24	19	4.41	4.44	4.60	4.57	4.00
Y27M-23_disgust	Young	Male	27	23	3.89	4.09	3.67	4.30	3.46
Y28M-20_disgust	Young	Male	28	20	4.57	4.38	4.73	4.62	4.57
Y29M-21_disgust	Young	Male	29	21	4.29	4.31	4.33	4.31	4.21
Y30M-25_disgust	Young	Male	30	25	3.61	3.75	3.87	3.77	3.00
Y33M-25_disgust	Young	Male	33	25	4.26	4.38	4.37	4.38	3.90
Y35M-20_disgust	Young	Male	35	20	4.27	4.28	4.33	4.27	4.18
Y36M-30_disgust	Young	Male	36	30	4.45	4.56	4.33	4.57	4.31
Y41M-19_disgust	Young	Male	41	19	3.93	4.00	3.87	4.00	3.85
Y44M-26_disgust	Young	Male	44	26	4.16	4.19	4.00	4.54	3.92
Y46M-18_disgust	Young	Male	46	18	4.37	4.38	4.60	4.54	3.92
Y47M-23_disgust	Young	Male	47	23	4.32	4.38	4.53	4.31	4.06
Y49M-23_disgust	Young	Male	49	23	3.81	3.88	3.87	3.77	3.69
Y53M-23_disgust	Young	Male	53	23	3.68	3.94	4.00	3.31	3.38
Y54M-26_disgust	Young	Male	54	26	3.97	4.19	3.93	4.00	3.69
Y55M-24_disgust	Young	Male	55	24	4.45	4.31	4.67	4.54	4.29
Y56M-24_disgust	Young	Male	56	24	4.40	4.63	4.53	4.38	4.00
Y57M-23_disgust	Young	Male	57	23	4.36	4.25	4.33	4.54	4.36
Y58M-22_disgust	Young	Male	58	22	4.11	4.13	4.00	4.31	4.00
Y60M-24_disgust	Young	Male	60	24	4.52	4.50	4.60	4.43	4.54
Y67M-19_disgust	Young	Male	67	19	4.52	4.50	4.73	4.62	4.21
Y68M-30_disgust	Young	Male	68	30	4.27	4.31	4.33	4.38	4.06
Y75M-30_disgust	Young	Male	75	30	3.55	3.31	3.80	3.62	3.50
O4F-76_disgust	Old	Female	4	76	4.47	4.56	4.53	4.69	4.13
O7F-65_disgust	Old	Female	7	65	3.64	3.56	4.13	3.85	3.00
O9F-64_disgust	Old	Female	9	64	4.23	4.38	4.33	4.46	3.69
O10F-60_disgust	Old	Female	10	60	3.42	3.44	3.07	3.77	3.46
O16F-64_disgust	Old	Female	16	64	3.81	3.63	4.40	3.46	3.71
O19F-60_disgust	Old	Female	19	60	3.71	3.56	3.93	4.00	3.36
O22F-61_disgust	Old	Female	22	61	3.79	3.94	4.00	3.77	3.43
O23F-66_disgust	Old	Female	23	66	4.12	4.31	4.07	4.14	3.92
O24F-62_disgust	Old	Female	24	62	4.55	4.69	4.60	4.64	4.23
O26F-64_disgust	Old	Female	26	64	3.88	4.00	4.20	4.00	3.23
O27F-65_disgust	Old	Female	27	65	4.18	4.44	4.67	3.92	3.69
O28F-64_disgust	Old	Female	28	64	3.55	3.75	3.87	3.38	3.14
O29F-63_disgust	Old	Female	29	63	4.48	4.50	4.67	4.38	4.36
O34F-65_disgust	Old	Female	34	65	4.15	4.22	4.33	4.27	3.80
O38F-65_disgust	Old	Female	38	65	4.18	4.19	4.40	4.38	3.81
O40F-61_disgust	Old	Female	40	61	4.43	4.31	4.67	4.23	4.50
O41F-72_disgust	Old	Female	41	72	4.18	4.44	4.33	4.23	3.62
O43F-62_disgust	Old	Female	43	62	4.50	4.44	4.73	4.69	4.14
O45F-65_disgust	Old	Female	45	65	4.17	4.13	4.53	4.00	4.00
O47F-60_disgust	Old	Female	47	60	4.29	4.25	4.60	4.38	3.93
O48F-65_disgust	Old	Female	48	65	4.40	4.50	4.53	4.46	4.13
O49F-65_disgust	Old	Female	49	65	4.40	4.69	4.13	4.71	4.00
O51F-60_disgust	Old	Female	51	60	3.48	3.44	3.67	4.00	2.86
O52F-62_disgust	Old	Female	52	62	4.15	4.34	4.27	4.35	3.59
O53F-64_disgust	Old	Female	53	64	4.15	4.31	4.13	4.30	3.81
O56F-65_disgust	Old	Female	56	65	4.21	4.25	4.53	4.23	3.77
O8M-65_disgust	Old	Male	8	65	3.95	3.94	4.20	3.92	3.75
O12M-64_disgust	Old	Male	12	64	4.20	4.00	4.53	4.31	4.00
O15M-69_disgust	Old	Male	15	69	4.60	4.56	4.60	4.69	4.54
O17M-69_disgust	Old	Male	17	69	4.05	4.00	4.27	4.38	3.63
O20M-65_disgust	Old	Male	20	65	4.16	4.19	4.33	4.23	3.85
O21M-65_disgust	Old	Male	21	65	3.93	4.19	4.27	3.69	3.56
O35M-66_disgust	Old	Male	35	66	4.28	4.25	4.40	4.36	4.08
O42M-75_disgust	Old	Male	42	75	4.58	4.44	4.73	4.62	4.56
O50M-65_disgust	Old	Male	50	65	3.97	4.00	4.13	3.85	3.88
O55M-64_disgust	Old	Male	55	64	4.40	4.63	4.20	4.62	4.15
O58M-64_disgust	Old	Male	58	64	3.60	3.63	4.00	3.65	3.11
O59M-65_disgust	Old	Male	59	65	4.12	3.94	4.27	4.14	4.15
O63M-61_disgust	Old	Male	63	61	3.60	3.41	3.53	4.12	3.41
O64M-65_disgust	Old	Male	64	65	4.32	4.56	4.27	4.31	4.13
O65M-65_disgust	Old	Male	65	65	4.46	4.13	4.73	4.62	4.38
O66M-70_disgust	Old	Male	66	70	4.12	4.19	4.07	4.38	3.86
O67M-61_disgust	Old	Male	67	61	3.53	4.06	3.33	4.00	2.62
O68M-60_disgust	Old	Male	68	60	3.37	3.19	3.53	3.69	3.08
O69M-62_disgust	Old	Male	69	62	3.82	3.84	4.00	3.96	3.48
O70M-66_disgust	Old	Male	70	66	3.93	3.63	4.13	4.23	3.81
O71M-65_disgust	Old	Male	71	65	3.98	3.75	4.20	4.15	3.86

Image Information					Perceived emotional intensity (1=lowerst, 5=highest)				
Image Name	Model Age group	Model Gender	Model Code	Actual Age (Years)	All Raters	Older Female raters	Young Female raters	Older Male raters	Younger Male raters
Y3F-20_surprised	Young	Female	3	20	4.31	4.31	4.60	4.38	3.93
Y4F-19_surprised	Young	Female	4	19	3.93	3.81	4.00	4.46	3.50
Y5F-24_surprised	Young	Female	5	24	3.47	3.69	3.20	3.92	3.08
Y6F-23_surprised	Young	Female	6	23	4.02	4.31	3.87	4.46	3.38
Y12F-18_surprised	Young	Female	12	18	4.22	4.38	4.13	4.31	4.06
Y13F-20_surprised	Young	Female	13	20	3.30	3.63	2.93	3.85	2.77
Y14F-21_surprised	Young	Female	14	21	4.07	4.13	4.00	4.31	3.85
Y17F-33_surprised	Young	Female	17	33	4.37	4.50	4.27	4.62	4.13
Y18F-18_surprised	Young	Female	18	18	4.21	4.31	4.33	4.38	3.79
Y19F-19_surprised	Young	Female	19	19	4.22	4.00	4.53	4.31	4.06
Y22F-30_surprised	Young	Female	22	30	4.09	4.13	3.87	4.46	3.92
Y23F-30_surprised	Young	Female	23	30	4.60	4.63	4.47	4.93	4.38
Y25F-33_surprised	Young	Female	25	33	4.55	4.63	4.73	4.62	4.25
Y26F-32_surprised	Young	Female	26	32	4.12	4.13	4.20	4.36	3.77
Y31F-30_surprised	Young	Female	31	30	4.28	4.44	4.33	4.43	3.85
Y32F-20_surprised	Young	Female	32	20	3.70	3.81	3.67	3.92	3.38
Y37F-32_surprised	Young	Female	37	32	4.24	4.31	4.33	4.38	3.93
Y38F-24_surprised	Young	Female	38	24	4.48	4.69	4.47	4.64	4.08
Y39F-25_surprised	Young	Female	39	25	4.44	4.25	4.53	4.69	4.31
Y40F-28_surprised	Young	Female	40	28	4.50	4.63	4.60	4.69	4.13
Y42F-20_surprised	Young	Female	42	20	3.98	4.00	4.00	4.23	3.75
Y48F-23_surprised	Young	Female	48	23	4.43	4.56	4.73	4.38	4.06
Y50F-24_surprised	Young	Female	50	24	4.00	4.13	3.87	4.50	3.46
Y51F-23_surprised	Young	Female	51	23	3.97	4.31	3.93	4.14	3.38
Y52F-21_surprised	Young	Female	52	21	4.28	4.44	4.07	4.54	4.08
Y59F-23_surprised	Young	Female	59	23	4.12	3.88	4.27	4.46	3.92
Y65F-21_surprised	Young	Female	65	21	3.53	3.75	3.40	3.77	3.21
Y66F-26_surprised	Young	Female	66	26	3.80	3.69	3.47	4.15	3.94
Y69F-28_surprised	Young	Female	69	28	4.08	4.06	4.40	4.38	3.56
Y71F-20_surprised	Young	Female	71	20	4.40	4.44	4.33	4.62	4.25
Y72F-25_surprised	Young	Female	72	25	4.25	4.44	4.27	4.31	3.92
Y1M-19_surprised	Young	Male	1	19	3.98	4.00	4.00	4.15	3.77
Y2M-21_surprised	Young	Male	2	21	3.98	4.06	4.20	4.15	3.50
Y8M-27_surprised	Young	Male	8	27	4.02	4.19	4.07	4.00	3.77
Y10M-22_surprised	Young	Male	10	22	4.47	4.44	4.93	4.54	3.93
Y11M-20_surprised	Young	Male	11	20	4.35	4.19	4.53	4.38	4.31
Y15M-20_surprised	Young	Male	15	20	3.98	4.13	3.80	4.54	3.46
Y16M-21_surprised	Young	Male	16	21	4.44	4.38	4.60	4.54	4.23
Y21M-21_surprised	Young	Male	21	21	4.29	4.50	4.53	4.38	3.71
Y24M-19_surprised	Young	Male	24	19	4.40	4.56	4.33	4.43	4.23
Y27M-23_surprised	Young	Male	27	23	3.40	3.56	3.47	3.77	2.88
Y28M-20_surprised	Young	Male	28	20	4.02	4.19	4.00	4.21	3.62
Y29M-21_surprised	Young	Male	29	21	4.33	4.31	4.60	4.23	4.15
Y30M-25_surprised	Young	Male	30	25	4.65	4.63	4.60	4.92	4.46
Y33M-25_surprised	Young	Male	33	25	4.03	4.06	4.33	4.15	3.57
Y35M-20_surprised	Young	Male	35	20	4.08	4.00	4.20	4.23	3.94
Y36M-30_surprised	Young	Male	36	30	4.18	4.38	4.13	4.46	3.69
Y41M-19_surprised	Young	Male	41	19	3.82	3.81	3.67	4.38	3.46
Y44M-26_surprised	Young	Male	44	26	4.37	4.44	4.27	4.62	4.19
Y46M-18_surprised	Young	Male	46	18	4.25	4.19	4.20	4.62	4.06
Y47M-23_surprised	Young	Male	47	23	3.76	4.13	3.67	4.00	3.15
Y49M-23_surprised	Young	Male	49	23	4.52	4.38	4.67	4.85	4.21
Y53M-23_surprised	Young	Male	53	23	4.28	4.13	4.53	4.38	4.07
Y54M-26_surprised	Young	Male	54	26	4.12	4.13	4.13	4.36	3.85
Y55M-24_surprised	Young	Male	55	24	4.48	4.56	4.47	4.71	4.15
Y56M-24_surprised	Young	Male	56	24	3.90	3.94	3.93	4.29	3.38
Y57M-23_surprised	Young	Male	57	23	4.35	4.38	4.27	4.62	4.15
Y58M-22_surprised	Young	Male	58	22	4.35	4.38	4.33	4.62	4.08
Y60M-24_surprised	Young	Male	60	24	4.26	4.25	4.33	4.46	4.00
Y67M-19_surprised	Young	Male	67	19	3.78	3.81	4.13	4.08	3.07
Y68M-30_surprised	Young	Male	68	30	4.29	4.44	4.00	4.64	4.08
Y74M-31_surprised	Young	Male	74	31	4.23	4.31	4.40	4.15	4.06
Y75M-30_surprised	Young	Male	75	30	3.85	4.00	3.87	4.15	3.44
O4F-76_surprised	Old	Female	4	76	4.55	4.63	4.33	4.79	4.46
O7F-65_surprised	Old	Female	7	65	4.28	4.31	4.53	4.38	3.86
O9F-64_surprised	Old	Female	9	64	4.35	4.50	4.47	4.54	3.94
O10F-60_surprised	Old	Female	10	60	4.22	4.25	4.33	4.31	4.00
O16F-64_surprised	Old	Female	16	64	4.38	4.25	4.40	4.71	4.15
O19F-60_surprised	Old	Female	19	60	4.24	4.13	4.53	4.46	3.86
O22F-61_surprised	Old	Female	22	61	4.32	4.19	4.40	4.62	4.13
O23F-66_surprised	Old	Female	23	66	4.47	4.63	4.33	4.71	4.15
O24F-62_surprised	Old	Female	24	62	4.16	4.25	3.87	4.57	3.92
O26F-64_surprised	Old	Female	26	64	4.26	4.50	4.33	4.46	3.69
O27F-65_surprised	Old	Female	27	65	3.64	3.69	3.87	4.00	2.92
O28F-64_surprised	Old	Female	28	64	4.22	4.44	4.33	4.38	3.71
O29F-63_surprised	Old	Female	29	63	4.60	4.50	4.80	4.69	4.38
O34F-65_surprised	Old	Female	34	65	4.30	4.13	4.53	4.46	4.08
O38F-65_surprised	Old	Female	38	65	4.42	4.31	4.33	4.62	4.46
O40F-61_surprised	Old	Female	40	61	4.07	3.88	4.27	4.31	3.86
O41F-72_surprised	Old	Female	41	72	4.33	4.56	4.47	4.46	3.79
O43F-62_surprised	Old	Female	43	62	4.28	4.31	4.40	4.54	3.86
O45F-65_surprised	Old	Female	45	65	4.18	4.44	4.07	4.38	3.77
O47F-60_surprised	Old	Female	47	60	4.25	4.31	4.20	4.62	3.94
O48F-65_surprised	Old	Female	48	65	4.23	4.25	4.27	4.38	4.06
O49F-65_surprised	Old	Female	49	65	4.47	4.56	4.47	4.77	4.08
O51F-60_surprised	Old	Female	51	60	4.12	4.31	4.27	4.43	3.38
O52F-62_surprised	Old	Female	52	62	3.79	3.88	3.90	4.04	3.33
O53F-64_surprised	Old	Female	53	64	4.07	4.00	4.20	4.38	3.75
O56F-65_surprised	Old	Female	56	65	4.40	4.06	4.60	4.77	4.25
O8M-65_surprised	Old	Male	8	65	4.47	4.50	4.53	4.46	4.38
O12M-64_surprised	Old	Male	12	64	4.37	4.44	4.47	4.38	4.19
O15M-69_surprised	Old	Male	15	69	4.80	4.81	4.87	4.77	4.75
O17M-69_surprised	Old	Male	17	69	4.12	4.38	4.00	4.38	3.69
O20M-65_surprised	Old	Male	20	65	4.28	4.06	4.40	4.50	4.15
O21M-65_surprised	Old	Male	21	65	3.97	4.16	3.83	4.37	3.52
O35M-66_surprised	Old	Male	35	66	3.93	3.75	4.27	4.31	3.43
O42M-75_surprised	Old	Male	42	75	4.29	4.44	4.27	4.71	3.69
O50M-65_surprised	Old	Male	50	65	4.40	4.56	4.40	4.50	4.08
O55M-64_surprised	Old	Male	55	64	4.38	4.44	4.40	4.57	4.08
O58M-64_surprised	Old	Male	58	64	4.30	4.31	4.27	4.77	3.94
O59M-65_surprised	Old	Male	59	65	3.76	3.81	3.73	4.29	3.15
O63M-61_surprised	Old	Male	63	61	3.81	4.00	4.00	4.14	3.00
O64M-65_surprised	Old	Male	64	65	4.28	4.25	4.47	4.43	3.92
O65M-65_surprised	Old	Male	65	65	3.05	3.31	3.00	3.29	2.54
O66M-70_surprised	Old	Male	66	70	3.84	3.75	4.00	4.31	3.36
O67M-61_surprised	Old	Male	67	61	3.67	4.06	3.60	4.08	2.93
O68M-60_surprised	Old	Male	68	60	4.31	4.50	4.47	4.54	3.71
O69M-62_surprised	Old	Male	69	62	4.22	4.38	4.47	4.31	3.71
O70M-66_surprised	Old	Male	70	66	4.10	4.13	4.27	4.23	3.81
O71M-65_surprised	Old	Male	71	65	4.40	4.38	4.40	4.71	4.08
