# Supplementary material for: Tsinghua facial expression database – A database of facial expressions in Chinese young and older women and men: Development and validation
Source: PLoS One. 2020 Apr 15;15(4):e0231304. doi: 10.1371/journal.pone.0231304 (PMC7159817; doi:10.1371/journal.pone.0231304)
Supplement: S4 Table — (PDF) [file pone.0231304.s004.pdf]

Model Age Group	Model Gender	Model Code	Actual Age (Years)	Image Name	Induction Phase
Young	Female	3	20	Y3F-20_happy	2
Young	Female	4	19	Y4F-19_happy	1
Young	Female	5	24	Y5F-24_happy	1
Young	Female	6	23	Y6F-23_happy	1
Young	Female	12	18	Y12F-18_happy	1
Young	Female	13	20	Y13F-20_happy	1
Young	Female	14	21	Y14F-21_happy	1
Young	Female	17	33	Y17F-33_happy	1
Young	Female	18	18	Y18F-18_happy	1
Young	Female	19	19	Y19F-19_happy	1
Young	Female	22	30	Y22F-30_happy	1
Young	Female	23	30	Y23F-30_happy	2
Young	Female	25	33	Y25F-33_happy	1
Young	Female	26	32	Y26F-32_happy	1
Young	Female	31	30	Y31F-30_happy	1
Young	Female	32	20	Y32F-20_happy	3
Young	Female	37	32	Y37F-32_happy	1
Young	Female	38	24	Y38F-24_happy	1
Young	Female	39	25	Y39F-25_happy	1
Young	Female	40	28	Y40F-28_happy	1
Young	Female	42	20	Y42F-20_happy	1
Young	Female	48	23	Y48F-23_happy	1
Young	Female	50	24	Y50F-24_happy	1
Young	Female	51	23	Y51F-23_happy	1
Young	Female	52	21	Y52F-21_happy	1
Young	Female	59	23	Y59F-23_happy	1
Young	Female	65	21	Y65F-21_happy	1
Young	Female	66	26	Y66F-26_happy	1
Young	Female	69	28	Y69F-28_happy	1
Young	Female	71	20	Y71F-20_happy	2
Young	Female	72	25	Y72F-25_happy	1
Young	Male	1	19	Y1M-19_happy	1
Young	Male	2	21	Y2M-21_happy	2
Young	Male	8	27	Y8M-27_happy	1
Young	Male	10	22	Y10M-22_happy	1
Young	Male	11	20	Y11M-20_happy	1
Young	Male	15	20	Y15M-20_happy	1
Young	Male	16	21	Y16M-21_happy	1
Young	Male	21	21	Y21M-21_happy	1
Young	Male	24	19	Y24M-19_happy	2
Young	Male	27	23	Y27M-23_happy	2
Young	Male	28	20	Y28M-20_happy	2
Young	Male	29	21	Y29M-21_happy	1
Young	Male	30	25	Y30M-25_happy	1
Young	Male	33	25	Y33M-25_happy	1
Young	Male	35	20	Y35M-20_happy	1
Young	Male	36	30	Y36M-30_happy	1
Young	Male	41	19	Y41M-19_happy	1
Young	Male	44	26	Y44M-26_happy	1
Young	Male	46	18	Y46M-18_happy	1
Young	Male	47	23	Y47M-23_happy	1
Young	Male	49	23	Y49M-23_happy	1
Young	Male	53	23	Y53M-23_happy	1
Young	Male	54	26	Y54M-26_happy	1
Young	Male	55	24	Y55M-24_happy	1
Young	Male	56	24	Y56M-24_happy	1
Young	Male	57	23	Y57M-23_happy	1
Young	Male	58	22	Y58M-22_happy	1
Young	Male	60	24	Y60M-24_happy	1
Young	Male	67	19	Y67M-19_happy	1
Young	Male	68	30	Y68M-30_happy	1
Young	Male	74	31	Y74M-31_happy	2
Young	Male	75	30	Y75M-30_happy	3
Old	Female	4	76	O04F-76_happy	3
Old	Female	7	65	O07F-65_happy	1
Old	Female	9	64	O09F-64_happy	1
Old	Female	10	60	O10F-60_happy	1
Old	Female	16	64	O16F-64_happy	1
Old	Female	19	60	O19F-60_happy	1
Old	Female	22	61	O22F-61_happy	1
Old	Female	23	66	O23F-66_happy	1
Old	Female	24	62	O24F-62_happy	1
Old	Female	26	64	O26F-64_happy	1
Old	Female	27	65	O27F-65_happy	1
Old	Female	28	64	O28F-64_happy	1
Old	Female	29	63	O29F-63_happy	2
Old	Female	34	65	O34F-65_happy	2
Old	Female	38	65	O38F-65_happy	2
Old	Female	40	61	O40F-61_happy	2
Old	Female	41	72	O41F-72_happy	1
Old	Female	43	62	O43F-62_happy	2
Old	Female	45	65	O45F-65_happy	1
Old	Female	47	60	O47F-60_happy	3
Old	Female	48	65	O48F-65_happy	3
Old	Female	49	65	O49F-65_happy	1
Old	Female	51	60	O51F-60_happy	1
Old	Female	52	62	O52F-62_happy	1
Old	Female	53	64	O53F-64_happy	1
Old	Female	56	65	O56F-65_happy	1
Old	Male	8	65	O08M-65_happy	1
Old	Male	12	64	O12M-64_happy	1
Old	Male	15	69	O15M-69_happy	1
Old	Male	17	69	O17M-69_happy	1
Old	Male	20	65	O20M-65_happy	1
Old	Male	21	65	O21M-65_happy	1
Old	Male	35	66	O35M-66_happy	2
Old	Male	42	75	O42M-75_happy	2
Old	Male	50	65	O50M-65_happy	1
Old	Male	55	64	O55M-64_happy	1
Old	Male	58	64	O58M-64_happy	1
Old	Male	59	65	O59M-65_happy	1
Old	Male	63	61	O63M-61_happy	1
Old	Male	64	65	O64M-65_happy	1
Old	Male	65	65	O65M-65_happy	1
Old	Male	66	70	O66M-70_happy	1
Old	Male	67	61	O67M-61_happy	1
Old	Male	68	60	O68M-60_happy	1
Old	Male	69	62	O69M-62_happy	1
Old	Male	70	66	O70M-66_happy	1
Old	Male	71	65	O71M-65_happy	1

Model Age Group	Model Gender	Model Code	Actual Age (Years)	Image Name	Induction Phase
Young	Female	3	20	Y3F-20_content	1
Young	Female	4	19	Y4F-19_content	1
Young	Female	5	24	Y5F-24_content	1
Young	Female	6	23	Y6F-23_content	1
Young	Female	12	18	Y12F-18_content	1
Young	Female	13	20	Y13F-20_content	1
Young	Female	14	21	Y14F-21_content	1
Young	Female	17	33	Y17F-33_content	1
Young	Female	18	18	Y18F-18_content	1
Young	Female	19	19	Y19F-19_content	1
Young	Female	22	30	Y22F-30_content	1
Young	Female	23	30	Y23F-30_content	1
Young	Female	25	33	Y25F-33_content	1
Young	Female	26	32	Y26F-32_content	1
Young	Female	31	30	Y31F-30_content	2
Young	Female	32	20	Y32F-20_content	1
Young	Female	37	32	Y37F-32_content	1
Young	Female	38	24	Y38F-24_content	1
Young	Female	39	25	Y39F-25_content	1
Young	Female	40	28	Y40F-28_content	2
Young	Female	42	20	Y42F-20_content	1
Young	Female	48	23	Y48F-23_content	1
Young	Female	50	24	Y50F-24_content	1
Young	Female	51	23	Y51F-23_content	1
Young	Female	52	21	Y52F-21_content	1
Young	Female	59	23	Y59F-23_content	1
Young	Female	65	21	Y65F-21_content	1
Young	Female	66	26	Y66F-26_content	1
Young	Female	69	28	Y69F-28_content	1
Young	Female	71	20	Y71F-20_content	1
Young	Female	72	25	Y72F-25_content	3
Young	Male	1	19	Y1M-19_content	1
Young	Male	2	21	Y2M-21_content	2
Young	Male	8	27	Y8M-27_content	1
Young	Male	10	22	Y10M-22_content	1
Young	Male	11	20	Y11M-20_content	1
Young	Male	15	20	Y15M-20_content	1
Young	Male	16	21	Y16M-21_content	1
Young	Male	21	21	Y21M-21_content	3
Young	Male	24	19	Y24M-19_content	2
Young	Male	27	23	Y27M-23_content	1
Young	Male	28	20	Y28M-20_content	1
Young	Male	29	21	Y29M-21_content	1
Young	Male	30	25	Y30M-25_content	1
Young	Male	33	25	Y33M-25_content	1
Young	Male	35	20	Y35M-20_content	1
Young	Male	36	30	Y36M-30_content	2
Young	Male	41	19	Y41M-19_content	3
Young	Male	44	26	Y44M-26_content	1
Young	Male	46	18	Y46M-18_content	1
Young	Male	47	23	Y47M-23_content	1
Young	Male	49	23	Y49M-23_content	1
Young	Male	53	23	Y53M-23_content	1
Young	Male	54	26	Y54M-26_content	1
Young	Male	55	24	Y55M-24_content	1
Young	Male	56	24	Y56M-24_content	1
Young	Male	57	23	Y57M-23_content	1
Young	Male	58	22	Y58M-22_content	1
Young	Male	60	24	Y60M-24_content	1
Young	Male	67	19	Y67M-19_content	1
Young	Male	68	30	Y68M-30_content	2
Young	Male	74	31	Y74M-31_content	1
Young	Male	75	30	Y75M-30_content	1
Old	Female	7	65	O07F-65_content	1
Old	Female	9	64	O09F-64_content	1
Old	Female	10	60	O10F-60_content	1
Old	Female	16	64	O16F-64_content	1
Old	Female	19	60	O19F-60_content	1
Old	Female	22	61	O22F-61_content	1
Old	Female	23	66	O23F-66_content	1
Old	Female	24	62	O24F-62_content	1
Old	Female	26	64	O26F-64_content	1
Old	Female	27	65	O27F-65_content	1
Old	Female	28	64	O28F-64_content	1
Old	Female	29	63	O29F-63_content	1
Old	Female	34	65	O34F-65_content	1
Old	Female	38	65	O38F-65_content	2
Old	Female	40	61	O40F-61_content	2
Old	Female	41	72	O41F-72_content	2
Old	Female	43	62	O43F-62_content	3
Old	Female	45	65	O45F-65_content	2
Old	Female	47	60	O47F-60_content	1
Old	Female	48	65	O48F-65_content	2
Old	Female	49	65	O49F-65_content	1
Old	Female	51	60	O51F-60_content	1
Old	Female	52	62	O52F-62_content	1
Old	Female	53	64	O53F-64_content	1
Old	Female	56	65	O56F-65_content	1
Old	Male	8	65	O08M-65_content	1
Old	Male	12	64	O12M-64_content	1
Old	Male	15	69	O15M-69_content	1
Old	Male	17	69	O17M-69_content	1
Old	Male	20	65	O20M-65_content	1
Old	Male	21	65	O21M-65_content	1
Old	Male	35	66	O35M-66_content	2
Old	Male	42	75	O42M-75_content	1
Old	Male	50	65	O50M-65_content	1
Old	Male	55	64	O55M-64_content	1
Old	Male	58	64	O58M-64_content	1
Old	Male	59	65	O59M-65_content	1
Old	Male	63	61	O63M-61_content	1
Old	Male	64	65	O64M-65_content	1
Old	Male	65	65	O65M-65_content	1
Old	Male	66	70	O66M-70_content	3
Old	Male	67	61	O67M-61_content	1
Old	Male	68	60	O68M-60_content	1
Old	Male	69	62	O69M-62_content	1
Old	Male	70	66	O70M-66_content	1
Old	Male	71	65	O71M-65_content	1

Model Age Group	Model Gender	Model Code	Actual Age (Years)	Image Name	Induction Phase
Young	Female	3	20	Y3F-20_sad	3
Young	Female	4	19	Y4F-19_sad	1
Young	Female	5	24	Y5F-24_sad	1
Young	Female	6	23	Y6F-23_sad	1
Young	Female	12	18	Y12F-18_sad	2
Young	Female	13	20	Y13F-20_sad	3
Young	Female	14	21	Y14F-21_sad	1
Young	Female	17	33	Y17F-33_sad	1
Young	Female	18	18	Y18F-18_sad	1
Young	Female	19	19	Y19F-19_sad	1
Young	Female	22	30	Y22F-30_sad	1
Young	Female	23	30	Y23F-30_sad	1
Young	Female	25	33	Y25F-33_sad_2	1
Young	Female	26	32	Y26F-32_sad	1
Young	Female	31	30	Y31F-30_sad	3
Young	Female	32	20	Y32F-20_sad	1
Young	Female	37	32	Y37F-32_sad	3
Young	Female	38	24	Y38F-24_sad	1
Young	Female	39	25	Y39F-25_sad	1
Young	Female	40	28	Y40F-28_sad	1
Young	Female	42	20	Y42F-20_sad	1
Young	Female	48	23	Y48F-23_sad	1
Young	Female	50	24	Y50F-24_sad	1
Young	Female	51	23	Y51F-23_sad	1
Young	Female	52	21	Y52F-21_sad	1
Young	Female	59	23	Y59F-23_sad	1
Young	Female	65	21	Y65F-21_sad	1
Young	Female	66	26	Y66F-26_sad	2
Young	Female	69	28	Y69F-28_sad	1
Young	Female	71	20	Y71F-20_sad	3
Young	Female	72	25	Y72F-25_sad	3
Young	Male	1	19	Y1M-19_sad	1
Young	Male	2	21	Y2M-21_sad	3
Young	Male	8	27	Y8M-27_sad	3
Young	Male	10	22	Y10M-22_sad	1
Young	Male	11	20	Y11M-20_sad	1
Young	Male	15	20	Y15M-20_sad	1
Young	Male	16	21	Y16M-21_sad	1
Young	Male	21	21	Y21M-21_sad	1
Young	Male	24	19	Y24M-19_sad	2
Young	Male	27	23	Y27M-23_sad	2
Young	Male	28	20	Y28M-20_sad	3
Young	Male	29	21	Y29M-21_sad	2
Young	Male	33	25	Y33M-25_sad	1
Young	Male	35	20	Y35M-20_sad	3
Young	Male	36	30	Y36M-30_sad	1
Young	Male	41	19	Y41M-19_sad	3
Young	Male	44	26	Y44M-26_sad	1
Young	Male	46	18	Y46M-18_sad	3
Young	Male	47	23	Y47M-23_sad	1
Young	Male	49	23	Y49M-23_sad	1
Young	Male	53	23	Y53M-23_sad	3
Young	Male	54	26	Y54M-26_sad	3
Young	Male	55	24	Y55M-24_sad	2
Young	Male	56	24	Y56M-24_sad	1
Young	Male	57	23	Y57M-23_sad	1
Young	Male	58	22	Y58M-22_sad	1
Young	Male	60	24	Y60M-24_sad	1
Young	Male	67	19	Y67M-19_sad	2
Young	Male	68	30	Y68M-30_sad	2
Young	Male	74	31	Y74M-31_sad	3
Young	Male	75	30	Y75M-30_sad	1
Old	Female	4	76	O04F-76_sad	2
Old	Female	7	65	O07F-65_sad	1
Old	Female	9	64	O09F-64_sad	1
Old	Female	10	60	O10F-60_sad	1
Old	Female	16	64	O16F-64_sad	3
Old	Female	19	60	O19F-60_sad	1
Old	Female	22	61	O22F-61_sad	3
Old	Female	23	66	O23F-66_sad	1
Old	Female	24	62	O24F-62_sad	1
Old	Female	26	64	O26F-64_sad	1
Old	Female	27	65	O27F-65_sad	1
Old	Female	28	64	O28F-64_sad	2
Old	Female	29	63	O29F-63_sad	3
Old	Female	34	65	O34F-65_sad	3
Old	Female	38	65	O38F-65_sad	3
Old	Female	40	61	O40F-61_sad	3
Old	Female	41	72	O41F-72_sad	3
Old	Female	43	62	O43F-62_sad	1
Old	Female	45	65	O45F-65_sad	2
Old	Female	47	60	O47F-60_sad	3
Old	Female	48	65	O48F-65_sad	2
Old	Female	49	65	O49F-65_sad	3
Old	Female	51	60	O51F-60_sad	1
Old	Female	52	62	O52F-62_sad	1
Old	Female	53	64	O53F-64_sad	1
Old	Female	56	65	O56F-65_sad	1
Old	Male	8	65	O08M-65_sad	3
Old	Male	12	64	O12M-64_sad	1
Old	Male	15	69	O15M-69_sad	1
Old	Male	17	69	O17M-69_sad	1
Old	Male	20	65	O20M-65_sad	1
Old	Male	21	65	O21M-65_sad	1
Old	Male	35	66	O35M-66_sad	2
Old	Male	42	75	O42M-75_sad	3
Old	Male	50	65	O50M-65_sad	1
Old	Male	55	64	O55M-64_sad	1
Old	Male	58	64	O58M-64_sad	2
Old	Male	59	65	O59M-65_sad	1
Old	Male	63	61	O63M-61_sad	1
Old	Male	64	65	O64M-65_sad	1
Old	Male	65	65	O65M-65_sad	3
Old	Male	66	70	O66M-70_sad	2
Old	Male	67	61	O67M-61_sad	1
Old	Male	68	60	O68M-60_sad	1
Old	Male	69	62	O69M-62_sad	3
Old	Male	70	66	O70M-66_sad	2
Old	Male	71	65	O71M-65_sad	1

Model Age Group	Model Gender	Model Code	Actual Age (Years)	Image Name	Induction Phase
Young	Female	3	20	Y3F-20_anger	1
Young	Female	4	19	Y4F-19_anger	1
Young	Female	5	24	Y5F-24_anger	2
Young	Female	6	23	Y6F-23_anger	1
Young	Female	12	18	Y12F-18_anger	1
Young	Female	13	20	Y13F-20_anger	1
Young	Female	14	21	Y14F-21_anger	1
Young	Female	17	33	Y17F-33_anger	1
Young	Female	18	18	Y18F-18_anger	1
Young	Female	19	19	Y19F-19_anger	1
Young	Female	22	30	Y22F-30_anger	1
Young	Female	23	30	Y23F-30_anger	1
Young	Female	25	33	Y25F-33_anger	2
Young	Female	26	32	Y26F-32_anger	1
Young	Female	31	30	Y31F-30_anger	2
Young	Female	32	20	Y32F-20_anger	1
Young	Female	37	32	Y37F-32_anger	3
Young	Female	38	24	Y38F-24_anger	1
Young	Female	39	25	Y39F-25_anger	1
Young	Female	40	28	Y40F-28_anger	1
Young	Female	42	20	Y42F-20_anger	1
Young	Female	48	23	Y48F-23_anger	1
Young	Female	50	24	Y50F-24_anger	1
Young	Female	51	23	Y51F-23_anger	1
Young	Female	52	21	Y52F-21_anger	1
Young	Female	59	23	Y59F-23_anger	1
Young	Female	65	21	Y65F-21_anger	1
Young	Female	66	26	Y66F-26_anger	2
Young	Female	69	28	Y69F-28_anger	3
Young	Female	71	20	Y71F-20_anger	1
Young	Female	72	25	Y72F-25_anger	2
Young	Male	1	19	Y1M-19_anger	1
Young	Male	2	21	Y2M-21_anger	3
Young	Male	8	27	Y8M-27_anger	1
Young	Male	10	22	Y10M-22_anger	1
Young	Male	11	20	Y11M-20_anger	1
Young	Male	15	20	Y15M-20_anger	3
Young	Male	16	21	Y16M-21_anger	1
Young	Male	21	21	Y21M-21_anger	1
Young	Male	24	19	Y24M-19_anger	2
Young	Male	27	23	Y27M-23_anger	3
Young	Male	28	20	Y28M-20_anger	1
Young	Male	29	21	Y29M-21_anger	1
Young	Male	33	25	Y33M-25_anger	1
Young	Male	35	20	Y35M-20_anger	2
Young	Male	36	30	Y36M-30_anger	1
Young	Male	41	19	Y41M-19_anger_1	3
Young	Male	44	26	Y44M-26_anger	1
Young	Male	46	18	Y46M-18_anger	1
Young	Male	47	23	Y47M-23_anger	1
Young	Male	49	23	Y49M-23_anger	1
Young	Male	53	23	Y53M-23_anger	1
Young	Male	54	26	Y54M-26_anger	1
Young	Male	55	24	Y55M-24_anger	1
Young	Male	56	24	Y56M-24_anger	1
Young	Male	57	23	Y57M-23_anger	1
Young	Male	58	22	Y58M-22_anger	1
Young	Male	60	24	Y60M-24_anger	1
Young	Male	67	19	Y67M-19_anger	1
Young	Male	68	30	Y68M-30_anger	1
Young	Male	74	31	Y74M-31_anger_1	2
Young	Male	75	30	Y75M-30_anger	1
Old	Female	4	76	O04F-76_anger	3
Old	Female	7	65	O07F-65_anger	1
Old	Female	9	64	O09F-64_anger	1
Old	Female	10	60	O10F-60_anger	3
Old	Female	16	64	O16F-64_anger	3
Old	Female	19	60	O19F-60_anger	1
Old	Female	22	61	O22F-61_anger	1
Old	Female	23	66	O23F-66_anger	1
Old	Female	24	62	O24F-62_anger	1
Old	Female	26	64	O26F-64_anger	1
Old	Female	27	65	O27F-65_anger	1
Old	Female	28	64	O28F-64_anger	1
Old	Female	29	63	O29F-63_anger	2
Old	Female	34	65	O34F-65_anger	2
Old	Female	38	65	O38F-65_anger	2
Old	Female	40	61	O40F-61_anger	2
Old	Female	41	72	O41F-72_anger	2
Old	Female	43	62	O43F-62_anger	1
Old	Female	45	65	O45F-65_anger	3
Old	Female	47	60	O47F-60_anger	3
Old	Female	48	65	O48F-65_anger	2
Old	Female	49	65	O49F-65_anger	1
Old	Female	51	60	O51F-60_anger	3
Old	Female	52	62	O52F-62_anger	1
Old	Female	53	64	O53F-64_anger	1
Old	Female	56	65	O56F-65_anger	1
Old	Male	8	65	O08M-65_anger	1
Old	Male	12	64	O12M-64_anger	3
Old	Male	15	69	O15M-69_anger	1
Old	Male	17	69	O17M-69_anger	1
Old	Male	20	65	O20M-65_anger	1
Old	Male	21	65	O21M-65_anger	1
Old	Male	35	66	O35M-66_anger	3
Old	Male	42	75	O42M-75_anger	1
Old	Male	50	65	O50M-65_anger	1
Old	Male	55	64	O55M-64_anger	1
Old	Male	58	64	O58M-64_anger	1
Old	Male	59	65	O59M-65_anger	2
Old	Male	63	61	O63M-61_anger	1
Old	Male	64	65	O64M-65_anger	1
Old	Male	65	65	O65M-65_anger	1
Old	Male	66	70	O66M-70_anger	1
Old	Male	67	61	O67M-61_anger	2
Old	Male	68	60	O68M-60_anger	1
Old	Male	69	62	O69M-62_anger	1
Old	Male	70	66	O70M-66_anger	1
Old	Male	71	65	O71M-65_anger	1

Model Age Group	Model Gender	Model Code	Actual Age (Years)	Image Name	Induction Phase
Young	Female	3	20	Y3F-20_fear	2
Young	Female	4	19	Y4F-19_fear	1
Young	Female	5	24	Y5F-24_fear	1
Young	Female	6	23	Y6F-23_fear	1
Young	Female	12	18	Y12F-18_fear	2
Young	Female	13	20	Y13F-20_fear	3
Young	Female	14	21	Y14F-21_fear	1
Young	Female	17	33	Y17F-33_fear	1
Young	Female	18	18	Y18F-18_fear	3
Young	Female	19	19	Y19F-19_fear	3
Young	Female	22	30	Y22F-30_fear	3
Young	Female	23	30	Y23F-30_fear	3
Young	Female	25	33	Y25F-33_fear	1
Young	Female	26	32	Y26F-32_fear	1
Young	Female	31	30	Y31F-30_fear	2
Young	Female	32	20	Y32F-20_fear	2
Young	Female	37	32	Y37F-32_fear	3
Young	Female	38	24	Y38F-24_fear	1
Young	Female	39	25	Y39F-25_fear	3
Young	Female	40	28	Y40F-28_fear	1
Young	Female	42	20	Y42F-20_fear	1
Young	Female	48	23	Y48F-23_fear	3
Young	Female	50	24	Y50F-24_fear	1
Young	Female	51	23	Y51F-23_fear	1
Young	Female	52	21	Y52F-21_fear	1
Young	Female	59	23	Y59F-23_fear	1
Young	Female	65	21	Y65F-21_fear	3
Young	Female	66	26	Y66F-26_fear	3
Young	Female	69	28	Y69F-28_fear	3
Young	Female	71	20	Y71F-20_fear	3
Young	Female	72	25	Y72F-25_fear	3
Young	Male	1	19	Y1M-19_fear	3
Young	Male	2	21	Y2M-21_fear	3
Young	Male	8	27	Y8M-27_fear	1
Young	Male	10	22	Y10M-22_fear	2
Young	Male	11	20	Y11M-20_fear	1
Young	Male	15	20	Y15M-20_fear	1
Young	Male	16	21	Y16M-21_fear	1
Young	Male	21	21	Y21M-21_fear	1
Young	Male	24	19	Y24M-19_fear	3
Young	Male	28	20	Y28M-20_fear	2
Young	Male	29	21	Y29M-21_fear	3
Young	Male	33	25	Y33M-25_fear	2
Young	Male	35	20	Y35M-20_fear	3
Young	Male	36	30	Y36M-30_fear	3
Young	Male	41	19	Y41M-19_fear	3
Young	Male	44	26	Y44M-26_fear	1
Young	Male	46	18	Y46M-18_fear	3
Young	Male	47	23	Y47M-23_fear	1
Young	Male	49	23	Y49M-23_fear	1
Young	Male	53	23	Y53M-23_fear	1
Young	Male	54	26	Y54M-26_fear	3
Young	Male	55	24	Y55M-24_fear	2
Young	Male	56	24	Y56M-24_fear	1
Young	Male	57	23	Y57M-23_fear	1
Young	Male	58	22	Y58M-22_fear	1
Young	Male	60	24	Y60M-24_fear	1
Young	Male	67	19	Y67M-19_fear	2
Young	Male	68	30	Y68M-30_fear	3
Young	Male	74	31	Y74M-31_fear	1
Young	Male	75	30	Y75M-30_fear	1
Old	Female	4	76	O04F-76_fear	3
Old	Female	7	65	O07F-65_fear	3
Old	Female	9	64	O09F-64_fear	1
Old	Female	10	60	O10F-60_fear	1
Old	Female	16	64	O16F-64_fear	1
Old	Female	19	60	O19F-60_fear	1
Old	Female	22	61	O22F-61_fear	1
Old	Female	23	66	O23F-66_fear	1
Old	Female	24	62	O24F-62_fear	1
Old	Female	26	64	O26F-64_fear	1
Old	Female	27	65	O27F-65_fear	3
Old	Female	28	64	O28F-64_fear	3
Old	Female	29	63	O29F-63_fear	3
Old	Female	34	65	O34F-65_fear	2
Old	Female	38	65	O38F-65_fear	3
Old	Female	40	61	O40F-61_fear	1
Old	Female	41	72	O41F-72_fear	3
Old	Female	43	62	O43F-62_fear	3
Old	Female	45	65	O45F-65_fear	3
Old	Female	47	60	O47F-60_fear	3
Old	Female	48	65	O48F-65_fear	2
Old	Female	49	65	O49F-65_fear	1
Old	Female	51	60	O51F-60_fear	1
Old	Female	52	62	O52F-62_fear	1
Old	Female	53	64	O53F-64_fear	1
Old	Female	56	65	O56F-65_fear	1
Old	Male	8	65	O08M-65_fear	3
Old	Male	12	64	O12M-64_fear	1
Old	Male	15	69	O15M-69_fear	2
Old	Male	17	69	O17M-69_fear	1
Old	Male	20	65	O20M-65_fear	1
Old	Male	21	65	O21M-65_fear	1
Old	Male	35	66	O35M-66_fear	3
Old	Male	42	75	O42M-75_fear	3
Old	Male	50	65	O50M-65_fear	1
Old	Male	55	64	O55M-64_fear	1
Old	Male	58	64	O58M-64_fear	1
Old	Male	59	65	O59M-65_fear	1
Old	Male	63	61	O63M-61_fear	1
Old	Male	64	65	O64M-65_fear	1
Old	Male	65	65	O65M-65_fear	3
Old	Male	66	70	O66M-70_fear	1
Old	Male	67	61	O67M-61_fear	2
Old	Male	68	60	O68M-60_fear	3
Old	Male	69	62	O69M-62_fear	2
Old	Male	70	66	O70M-66_fear	3
Old	Male	71	65	O71M-65_fear	1

Model Age Group	Model Gender	Model Code	Actual Age (Years)	Image Name	nduction Phas
Young	Female	3	20	Y3F-20_disgust	1
Young	Female	4	19	Y4F-19_disgust	1
Young	Female	5	24	Y5F-24_disgust	1
Young	Female	6	23	Y6F-23_disgust	1
Young	Female	12	18	Y12F-18_disgust	2
Young	Female	13	20	Y13F-20_disgust	1
Young	Female	14	21	Y14F-21_disgust	1
Young	Female	17	33	Y17F-33_disgust	1
Young	Female	18	18	Y18F-18_disgust	1
Young	Female	19	19	Y19F-19_disgust	1
Young	Female	22	30	Y22F-30_disgust	3
Young	Female	23	30	Y23F-30_disgust	1
Young	Female	25	33	Y25F-33_disgust	2
Young	Female	26	32	Y26F-32_disgust	1
Young	Female	31	30	Y31F-30_disgust	1
Young	Female	32	20	Y32F-20_disgust	1
Young	Female	37	32	Y37F-32_disgust	1
Young	Female	38	24	Y38F-24_disgust	1
Young	Female	39	25	Y39F-25_disgust	1
Young	Female	40	28	Y40F-28_disgust	1
Young	Female	42	20	Y42F-20_disgust	1
Young	Female	48	23	Y48F-23_disgust	1
Young	Female	50	24	Y50F-24_disgust	1
Young	Female	51	23	Y51F-23_disgust	1
Young	Female	52	21	Y52F-21_disgust	3
Young	Female	59	23	Y59F-23_disgust	1
Young	Female	65	21	Y65F-21_disgust	2
Young	Female	66	26	Y66F-26_disgust	1
Young	Female	69	28	Y69F-28_disgust	3
Young	Female	71	20	Y71F-20_disgust	2
Young	Female	72	25	Y72F-25_disgust	3
Young	Male	1	19	Y1M-19_disgust	1
Young	Male	2	21	Y2M-21_disgust	3
Young	Male	8	27	Y8M-27_disgust	1
Young	Male	10	22	Y10M-22_disgust	1
Young	Male	11	20	Y11M-20_disgust	1
Young	Male	15	20	Y15M-20_disgust	1
Young	Male	16	21	Y16M-21_disgust	1
Young	Male	21	21	Y21M-21_disgust	1
Young	Male	24	19	Y24M-19_disgust	2
Young	Male	27	23	Y27M-23_disgust	3
Young	Male	28	20	Y28M-20_disgust	3
Young	Male	29	21	Y29M-21_disgust	3
Young	Male	33	25	Y33M-25_disgust	1
Young	Male	35	20	Y35M-20_disgust	3
Young	Male	36	30	Y36M-30_disgust_1	1
Young	Male	41	19	Y41M-19_disgust	1
Young	Male	44	26	Y44M-26_disgust	1
Young	Male	46	18	Y46M-18_disgust	1
Young	Male	47	23	Y47M-23_disgust	1
Young	Male	49	23	Y49M-23_disgust	1
Young	Male	53	23	Y53M-23_disgust	1
Young	Male	54	26	Y54M-26_disgust	3
Young	Male	55	24	Y55M-24_disgust	1
Young	Male	56	24	Y56M-24_disgust	1
Young	Male	57	23	Y57M-23_disgust	1
Young	Male	58	22	Y58M-22_disgust	1
Young	Male	60	24	Y60M-24_disgust	2
Young	Male	67	19	Y67M-19_disgust	2
Young	Male	68	30	Y68M-30_disgust	2
Young	Male	75	30	Y75M-30_disgust	3
Old	Female	4	76	O04F-76_disgust	1
Old	Female	7	65	O07F-65_disgust	1
Old	Female	9	64	O09F-64_disgust	1
Old	Female	10	60	O10F-60_disgust	3
Old	Female	16	64	O16F-64_disgust	1
Old	Female	19	60	O19F-60_disgust	1
Old	Female	22	61	O22F-61_disgust	1
Old	Female	23	66	O23F-66_disgust	1
Old	Female	24	62	O24F-62_disgust	1
Old	Female	26	64	O26F-64_disgust	1
Old	Female	27	65	O27F-65_disgust	1
Old	Female	28	64	O28F-64_disgust	3
Old	Female	29	63	O29F-63_disgust	2
Old	Female	34	65	O34F-65_disgust	2
Old	Female	38	65	O38F-65_disgust	3
Old	Female	40	61	O40F-61_disgust	2
Old	Female	41	72	O41F-72_disgust	2
Old	Female	43	62	O43F-62_disgust	1
Old	Female	45	65	O45F-65_disgust	2
Old	Female	47	60	O47F-60_disgust	3
Old	Female	48	65	O48F-65_disgust	3
Old	Female	49	65	O49F-65_disgust	1
Old	Female	51	60	O51F-60_disgust	1
Old	Female	52	62	O52F-62_disgust	1
Old	Female	53	64	O53F-64_disgust	1
Old	Female	56	65	O56F-65_disgust	1
Old	Male	8	65	O08M-65_disgust	3
Old	Male	12	64	O12M-64_disgust	1
Old	Male	15	69	O15M-69_disgust	1
Old	Male	17	69	O17M-69_disgust	1
Old	Male	20	65	O20M-65_disgust	1
Old	Male	21	65	O21M-65_disgust	1
Old	Male	35	66	O35M-66_disgust	3
Old	Male	42	75	O42M-75_disgust	3
Old	Male	50	65	O50M-65_disgust	1
Old	Male	55	64	O55M-64_disgust	1
Old	Male	58	64	O58M-64_disgust	1
Old	Male	59	65	O59M-65_disgust	1
Old	Male	63	61	O63M-61_disgust	3
Old	Male	64	65	O64M-65_disgust	1
Old	Male	65	65	O65M-65_disgust	1
Old	Male	66	70	O66M-70_disgust	1
Old	Male	67	61	O67M-61_disgust	2
Old	Male	68	60	O68M-60_disgust	2
Old	Male	69	62	O69M-62_disgust	3
Old	Male	70	66	O70M-66_disgust	3
Old	Male	71	65	O71M-65_disgust	3

Model Age Group	Model Gender	Model Code	Actual Age (Years)	Image Name	Induction Phase
Young	Female	3	20	Y3F-20_surprise	1
Young	Female	4	19	Y4F-19_surprise	1
Young	Female	5	24	Y5F-24_surprise	2
Young	Female	6	23	Y6F-23_surprise	1
Young	Female	12	18	Y12F-18_surprise	1
Young	Female	13	20	Y13F-20_surprise	3
Young	Female	14	21	Y14F-21_surprise	1
Young	Female	17	33	Y17F-33_surprise	1
Young	Female	18	18	Y18F-18_surprise	1
Young	Female	19	19	Y19F-19_surprise	3
Young	Female	22	30	Y22F-30_surprise	1
Young	Female	23	30	Y23F-30_surprise	1
Young	Female	25	33	Y25F-33_surprise	2
Young	Female	26	32	Y26F-32_surprise	1
Young	Female	31	30	Y31F-30_surprise	2
Young	Female	32	20	Y32F-20_surprise	3
Young	Female	37	32	Y37F-32_surprise	3
Young	Female	38	24	Y38F-24_surprise	1
Young	Female	39	25	Y39F-25_surprise	1
Young	Female	40	28	Y40F-28_surprise	3
Young	Female	42	20	Y42F-20_surprise	1
Young	Female	48	23	Y48F-23_surprise	1
Young	Female	50	24	Y50F-24_surprise	1
Young	Female	51	23	Y51F-23_surprise	3
Young	Female	52	21	Y52F-21_surprise	1
Young	Female	59	23	Y59F-23_surprise	1
Young	Female	65	21	Y65F-21_surprise	2
Young	Female	66	26	Y66F-26_surprise	1
Young	Female	69	28	Y69F-28_surprise	3
Young	Female	71	20	Y71F-20_surprise	2
Young	Female	72	25	Y72F-25_surprise	3
Young	Male	1	19	Y1M-19_surprise	2
Young	Male	2	21	Y2M-21_surprise	3
Young	Male	8	27	Y8M-27_surprise	1
Young	Male	10	22	Y10M-22_surprise	1
Young	Male	11	20	Y11M-20_surprise	1
Young	Male	15	20	Y15M-20_surprise	1
Young	Male	16	21	Y16M-21_surprise	1
Young	Male	21	21	Y21M-21_surprise	1
Young	Male	24	19	Y24M-19_surprise	3
Young	Male	27	23	Y27M-23_surprise	3
Young	Male	28	20	Y28M-20_surprise	1
Young	Male	29	21	Y29M-21_surprise	1
Young	Male	30	25	Y30M-25_surprise	1
Young	Male	33	25	Y33M-25_surprise	2
Young	Male	35	20	Y35M-20_surprise	1
Young	Male	36	30	Y36M-30_surprise	2
Young	Male	41	19	Y41M-19_surprise	1
Young	Male	44	26	Y44M-26_surprise	1
Young	Male	46	18	Y46M-18_surprise	1
Young	Male	47	23	Y47M-23_surprise	1
Young	Male	49	23	Y49M-23_surprise	1
Young	Male	53	23	Y53M-23_surprise	1
Young	Male	54	26	Y54M-26_surprise	1
Young	Male	55	24	Y55M-24_surprise	1
Young	Male	56	24	Y56M-24_surprise	1
Young	Male	57	23	Y57M-23_surprise	1
Young	Male	58	22	Y58M-22_surprise	1
Young	Male	60	24	Y60M-24_surprise	1
Young	Male	67	19	Y67M-19_surprise	1
Young	Male	68	30	Y68M-30_surprise	3
Young	Male	74	31	Y74M-31_surprise	2
Young	Male	75	30	Y75M-30_surprise	1
Old	Female	4	76	O04F-76_surprise	3
Old	Female	7	65	O07F-65_surprise	3
Old	Female	9	64	O09F-64_surprise	1
Old	Female	10	60	O10F-60_surprise	1
Old	Female	16	64	O16F-64_surprise	1
Old	Female	19	60	O19F-60_surprise	1
Old	Female	22	61	O22F-61_surprise	1
Old	Female	23	66	O23F-66_surprise	1
Old	Female	24	62	O24F-62_surprise	1
Old	Female	26	64	O26F-64_surprise	1
Old	Female	27	65	O27F-65_surprise	1
Old	Female	28	64	O28F-64_surprise	3
Old	Female	29	63	O29F-63_surprise	3
Old	Female	34	65	O34F-65_surprise	2
Old	Female	38	65	O38F-65_surprise	3
Old	Female	40	61	O40F-61_surprise	3
Old	Female	41	72	O41F-72_surprise	3
Old	Female	43	62	O43F-62_surprise	3
Old	Female	45	65	O45F-65_surprise	2
Old	Female	47	60	O47F-60_surprise	2
Old	Female	48	65	O48F-65_surprise	2
Old	Female	49	65	O49F-65_surprise	1
Old	Female	51	60	O51F-60_surprise	1
Old	Female	52	62	O52F-62_surprise	1
Old	Female	53	64	O53F-64_surprise	1
Old	Female	56	65	O56F-65_surprise	1
Old	Male	8	65	O08M-65_surprise	1
Old	Male	12	64	O12M-64_surprise	1
Old	Male	15	69	O15M-69_surprise	1
Old	Male	17	69	O17M-69_surprise	1
Old	Male	20	65	O20M-65_surprise	1
Old	Male	21	65	O21M-65_surprise	1
Old	Male	35	66	O35M-66_surprise	2
Old	Male	42	75	O42M-75_surprise	2
Old	Male	50	65	O50M-65_surprise	1
Old	Male	55	64	O55M-64_surprise	1
Old	Male	58	64	O58M-64_surprise	1
Old	Male	59	65	O59M-65_surprise	1
Old	Male	63	61	O63M-61_surprise	1
Old	Male	64	65	O64M-65_surprise	1
Old	Male	65	65	O65M-65_surprise	3
Old	Male	66	70	O66M-70_surprise	1
Old	Male	67	61	O67M-61_surprise	1
Old	Male	68	60	O68M-60_surprise	2
Old	Male	69	62	O69M-62_surprise	2
Old	Male	70	66	O70M-66_surprise	2
Old	Male	71	65	O71M-65_surprise	2

Note: It should be noted that the emotion can be accumulated gradually along the inductions, therefore we cannot confidently say that the emotional facial expression displayed on the final image were fully induced by the corresponding induction phase [especially the 2nd (personal event induction) and 3rd (controlled facial expression induction phase) due to the possible accumulation from previous induction/inductions].
