# Supplementary material for: Tsinghua facial expression database – A database of facial expressions in Chinese young and older women and men: Development and validation
Source: PLoS One. 2020 Apr 15;15(4):e0231304. doi: 10.1371/journal.pone.0231304 (PMC7159817; doi:10.1371/journal.pone.0231304)
Supplement: S5 Table — (PDF) [file pone.0231304.s005.pdf]

		scenario induction	personal event induction	controlled facial expression
Happy	Young	84.1	12.7	3.2
	Old	78.7	14.9	6.4
Content	Young	85.7	9.5	4.8
	Old	82.6	13.0	4.4
Sad	Young	62.9	12.9	24.2
	Old	55.3	17.0	27.7
Anger	Young	77.4	12.9	9.7
	Old	66.0	17.0	17.0
Fear	Young	47.5	14.8	37.7
	Old	55.3	10.6	34.0
Disgust	Young	68.9	13.1	18.0
	Old	59.6	14.9	25.5
Surprise	Young	66.7	14.3	19.1
	Old	59.6	21.3	19.2
Overall	Young	70.5	12.9	16.7
	Old	65.3	15.5	19.2
