# Supplementary material for: Tsinghua facial expression database – A database of facial expressions in Chinese young and older women and men: Development and validation
Source: PLoS One. 2020 Apr 15;15(4):e0231304. doi: 10.1371/journal.pone.0231304 (PMC7159817; doi:10.1371/journal.pone.0231304)
Supplement: S1 Data — (PDF) [file pone.0231304.s006.pdf]

Scenarios used in the *scenario induction phase*

Target emotion	Scenarios
contentment:	▪ Enjoying a piece of your favorite cake.<br>▪ Having a warm shower after being soaked by rain outside.<br>▪ Sitting in a cozy balcony and enjoying your favorite book in a beautiful day.<br>▪ Just completed your work task that you have put off for a long time.<br>▪ Eating a big meal when you feel very hungry.<br>▪ Drinking a big glass of water when you feel very thirsty.
anger	▪ You or your family are being insulted/unreasonably treated (e.g. rude words or actions) by others in public.<br>▪ Your own holiday plan (you have expected for a long time) is suddenly cancelled by others.<br>▪ Your mobile was just stolen.<br>▪ Seeing someone abusing/maltreating your pets/animals.<br>▪ Seeing someone abusing/maltreating older people/their own parents.<br>▪ Someone is opening your drawer/touching your personal belongings without your permission.
fear	▪ The lift you are taking suddenly drops very fast.<br>▪ Seeing a rat/spider/snake in your room.<br>▪ A big fire just broke out in your building.<br>▪ Someone started to follow you at night when you walk by yourself in a quiet road where crime rate is high.<br>▪ Hearing loud and rushed door knocks during the night when you are alone at home.<br>▪ You are just told that a large-scale infectious disease broke out in your city.
sad	▪ You were just told that your close family member/friend was diagnosed with serious illness today.<br>▪ You were just told that your close family member/friend passed away today.<br>▪ Seeing poor young children who lost their parents during 512 earthquake on TV.<br>▪ Breaking up with your boyfriend/girlfriend/married partner.<br>▪ You just failed a very important exam.<br>▪ Lost a gift that is very important to you.
disgust	▪ Seeing a lot of dead flies on your desk.<br>▪ You just saw some bugs/flies in your food that you are eating.<br>▪ Someone is eating maggots on TV.

	▪ Stepping on a worm with bare feet and feeling the liquid ejected from the worm.<br>▪ Accidentally picked up a piece of paper on the table that was covered with slimy liquid.<br>▪ Someone is sneezing directly in front of your face without covering their mouth and nose.
surprise	▪ Seeing a UFO.<br>▪ Just bought something for 10 yuan, and others said it cost him 100 yuan.<br>▪ Just heard that your neighbor won a big lottery and he decided to donate it all to the public.<br>▪ You only got to know a big shocking news that happened last week (an important celebrity/politician passed away).<br>▪ Just heard your friend/family who is not good academically received an offer from Harvard.<br>▪ Just heard that a 90-year old person is applying for a university entry examination.
happy	▪ You just won a 500 million lottery.<br>▪ You/your child just got his/her dream job offer/university offer.<br>▪ Someone you like just confessed their love to you.<br>▪ Meeting your loved one/best friend who you have not seen for a long time.<br>▪ You are having a wonderful time with your loved one/family/grandchildren.<br>▪ You are celebrating your best friend's wedding/birthday.

[Note 1: These scenarios were top rated six scenarios from an online survey we conducted that aimed to identify scenarios that can elicit people's different emotional arousal. In the online survey (Wenjuanxing, China), participants [n=188 (male: 30.85%, female: 69.15%), mean age: 24.55 years, age range: 18 – 56 years] were asked to read around twelve scenarios (gathered from a research discussion group) for each target emotion (anger, content, happiness, sadness, fear, surprise, disgust), and they were asked to choose the top three scenarios that could maximumly elicit their corresponding emotional arousal.]

[Note 2: the scenarios/events trigger the specific emotion might differ between cultures (Ekman, 1970). Therefore, it might be suitable to be used to trigger the facial expression of people of other cultures].
