# Supplementary material for: Tsinghua facial expression database – A database of facial expressions in Chinese young and older women and men: Development and validation
Source: PLoS One. 2020 Apr 15;15(4):e0231304. doi: 10.1371/journal.pone.0231304 (PMC7159817; doi:10.1371/journal.pone.0231304)
Supplement: S2 Data — (DOCX) [file pone.0231304.s007.docx]

**Additional Findings**

**Results**

**Identification accuracy**

To examine the effects of Rater Age, Face Age and Facial Expression on modulating identification accuracy, the perceptual performance of the two rater groups was analysed using a 2 (Rater Age: Young, Older) × 2 (Face Age: Young, Older) × 8 (Facial Expression: Anger, Content, Disgust, Fear, Happiness, Neutral, Sadness, Surprise) mixed ANOVA (Table 1 and Fig 1 presents the results).

**Table 1. Results of ANOVA identifying the effects of facial expression, face age, and rater age (young vs. old) on identification accuracy.**

(Note: the Greenhouse-Geisser correction was employed when the assumption of sphericity had been violated. This also applies to other analysis reported in this study when necessary)

The results revealed that young raters’ overall mean identification accuracy (*M* = 82.65, *SD* = 12.11) was significantly higher than older raters (*M* = 73.24, *SD* = 16.22) [the main effect of Rater Age was significant, F(1, 63) = 26.082*, p <* .001*, η^2^=* .293], regardless of Facial Expression and Face Age. The main effect of Facial Expression was significant [F(4.241, 267.207) = 54.424*, p <* .001*, η^2^=* .463] (the Greenhouse-Geisser correction was employed when the assumption of sphericity had been violated. This also applies to other analysis reported in this study when necessary), indicating that identification accuracy for different facial expressions significantly differed, regardless of Rater Age and Face Age. The overall accuracy pattern indicated that raters were best at identifying Happiness and Content, while worst at identifying Fear and Disgust. The main effect of Face Age was significant [F(1, 63) =182.397*, p <* .001*, η^2^=* .743], indicating that raters’ overall accuracy for identifying young faces (*M* = 80.67, *SD* = 13.43) was significantly higher than for old faces (*M* = 75.22, *SD* = 14.90). This finding in parallel with prior studies stating that the wrinkles and folds of older faces can reduce the signal clarity of facial expressions [45, 46].

The interaction of Rater Age and Face Age was significant [F(1, 63) = 5.693*, p =* .020*, η^2^ =* .083], further analysis revealed that older raters were significantly worse than young raters at identifying facial expressions from both older (older raters: *M* = 70.99, *SD* = 16.70, young raters: *M* = 79.44, *SD* = 13.10) and young faces (older raters: *M* = 75.48, *SD* = 15.74, young raters: *M* = 85.86, *SD* = 11.12), and older raters’ poorer perceptual performance was more pronounced for older faces (*M* = 70.99, *SD* = 16.70) than for young faces (*M* = 75.48, *SD* = 15.74) [*p* < .001, *d* = 1.406, with Bonferroni correction]. In addition, within-group comparisons revealed that both young and older raters were better at identifying facial expressions from young faces (older raters: *M* = 75.48, *SD* = 15.74, young raters: *M* = 85.86, *SD* = 11.12) than from older faces (older raters: *M* = 70.99, *SD* = 16.70, young raters: *M* = 79.44, *SD* = 13.10) [young raters: *p* < .001, *d* = 2.693; older raters: *p* < .001, *d* = 1.944].

The interaction of Facial Expression × Face Age was significant [F(5.136, 323.588) = 6.567, *p <* .001*, η^2^=* .094]. Pairwise comparisons (with Bonferroni correction) revealed that the overall identification accuracy for young faces (*M* = 80.67, *SD* = 13.43) was significantly higher than for older faces (*M* = 75.22, *SD* = 14.90) when identifying all facial expressions except Fear and Happiness [Anger, *p* < .001, *d* = 1.761; Content, *p* < .001, *d* = 1.023; Disgust, *p* = .008, *d* = .852; Neutral, *p* < .001, *d* = 1.832; Sadness, *p* < .001, *d* = .941; Surprise, *p* < .001, *d* = 1.216], regardless of Rater Age.

The interaction of Facial Expression × Rater Age × Face Age was significant [F(7, 441) = 355.171, *p <* .001, *η^2^=* .101]. On the basis of findings that older adults have declined ability in identifying facial expressions [see a review 19], planned pairwise comparisons (Table 2 and Fig 1) revealed that older raters’ performance at identifying Happiness and Content did not significantly differ from young raters, while older rater were significantly worse than younger raters at identifying Anger, Disgust, Fear, Surprise, Neutral, Sadness and Surprise from either young/older faces, or both. Specifically, older raters performed significantly poorer than young raters at identifying Anger (*p* = .018, *d* = .601) and Sadness (*p* < .001, *d* = 1.261) from young faces; identifying Disgust (*p* = .009, *d* = .674) and Happiness (*p* = .028, *d* = .528) from older faces; and identifying Neutral (young face: *p* = .002, *d* = .802; older face: *p* = .037, *d* = .528), Fear (young face: *p* < .001, *d* = 1.140; older face: *p* = .002, *d* = .796) and Surprise (young face: *p* < .001, *d* = 1.000; older face: *p* < .001, *d* =1.046) from both young and older faces. There is a tendency that older raters’ poorer performance compared to younger raters in recognising facial expressions fear and neutral is more pronounced for young than for older faces; while older raters’ worse performance compared to younger raters in recognising surprise is more affected for older than for young faces.

**Table 2. Planned comparisons of older and young raters’ identification accuracy on all types of images.**

**
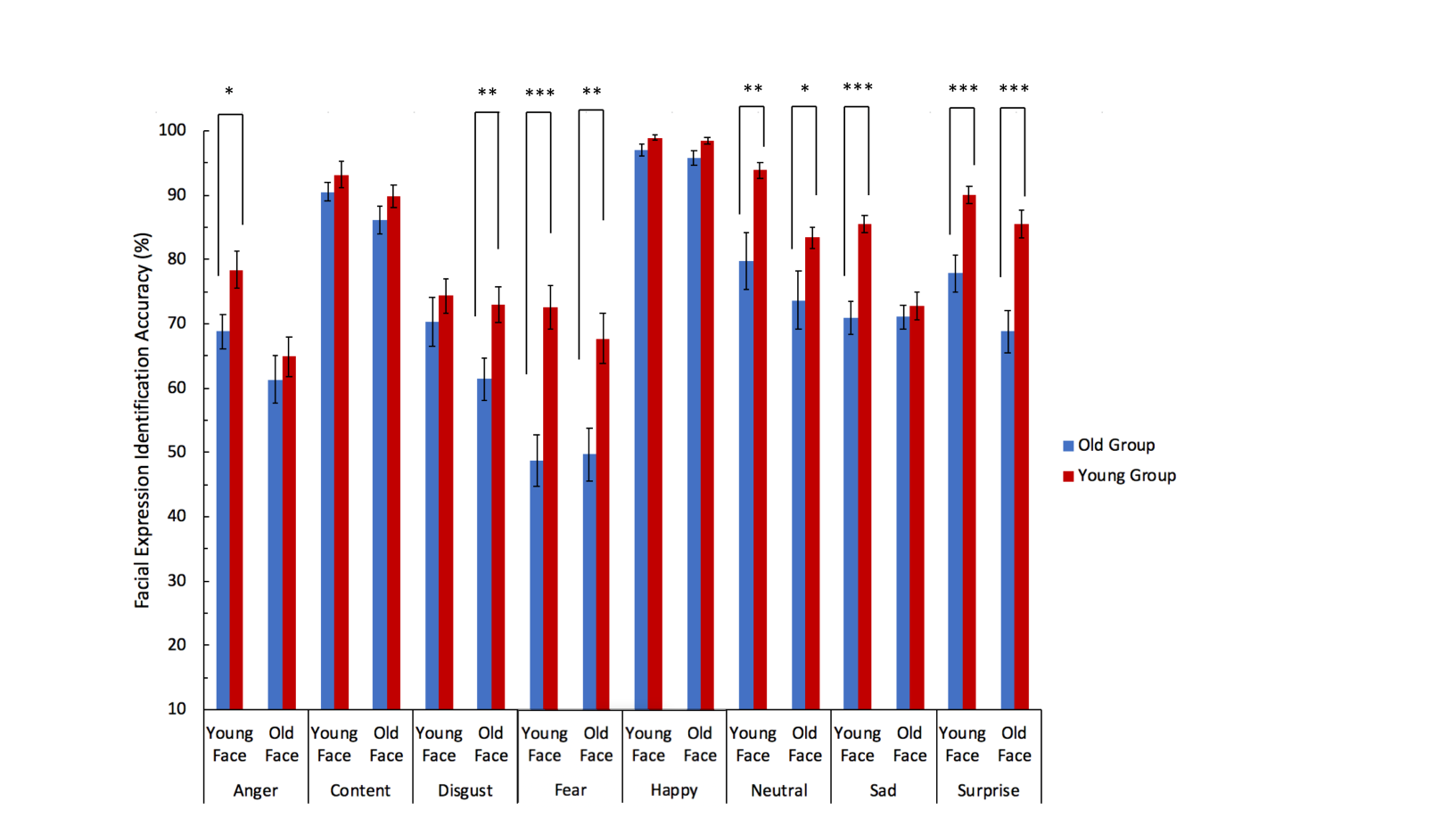
**

**Fig 1. Mean facial expression identification accuracy (%) of older and young groups. Blue bars represent old group, red bars represent young group.** Older raters performed significantly poorer than young raters at 1) identifying Anger and Sadness from young faces; 2) identifying Disgust from older faces; and 3) identifying Neutral, Fear and Surprise from both young and older faces. **(**Note: Error bars represents S.E. *** represents *p* <.001. ** represents *p* <.01. * represents *p* <.05.).

***Perceived emotional intensity***

To examine the effects of Rater Age, Facial Expression, and Face Age on modulating perceived intensity, the perceived intensity scores of the two rater groups were analysed using a 2 (Rater Age: Young, Older) × 2 (Face Age: Young, Older) × 7 (Facial Expression: Anger, Content, Disgust, Fear, Happiness, Neutral, Sadness, and Surprise)(Neutral was not included in this analysis) mixed ANOVA (Table 3 and Fig 2 present the results).

The main effect of Rater Age was significant, indicating that older raters’ overall mean perceived intensity (*M* = 4.05, *SD* = .52) was significantly higher than younger raters (*M* = 3.67 *SD* = .64), regardless of Facial Expression and Face Age. The main effect of Facial Expression was significant, indicating that raters’ perceived intensity differed between different facial expressions. The interaction between Facial Expression × Rater Age was significant. Pairwise comparisons (with Bonferroni correction) revealed that older raters’ mean perceived intensity was significantly higher than younger raters on facial expressions of Anger (older raters: *M* = 3.83, *SD* = .58, young raters: *M* = 3.24, *SD* = .77) (*p* = .007, *d* = .869), Content (older raters: *M* = 4.02, *SD* = .58, young raters: *M* = 3.41, *SD* = .69) (*p* <.001, *d* = .969 ) and Happiness (older raters: *M* = 4.39, *SD* = .47, young raters: *M* = 3.93 , *SD* = .63) (*p* =.007, *d* =.829), not on other facial expressions (Fig 2). In addition, the interaction of Facial Expression × Face Age was significant. Pairwise comparison (with Bonferroni correction) revealed that raters’ overall mean perceived intensity for Content (older faces: *M* = 3.66, *SD* = .72, young faces: *M* = 3.75, *SD* = .70) (*p* = .014, *d* = .810) and Fear (older faces: *M* = 3.93, *SD* = .54, young faces: *M* = 4.01, *SD* = .51) (*p* < .001, *d* = .935) were significantly higher from young faces than from older faces; whereas raters’ overall mean perceived intensity for Anger (older faces: *M* = 3.58, *SD* = .73, young faces: *M* = 3.46, *SD* = .75) (*p* = .007, *d* = .871) and Sadness (older faces: *M* = 3.44, *SD* = .71, young faces: *M* = 3.27, *SD* = .72) (*p* < .001, *d* = 1.103) were significantly higher from older faces than from young faces (Fig 2). No other effects were significant.

**Table 3. Results of ANOVA identifying the effects of facial expression, face age, and rater age (young vs. old) on perceived emotional intensity.**

**
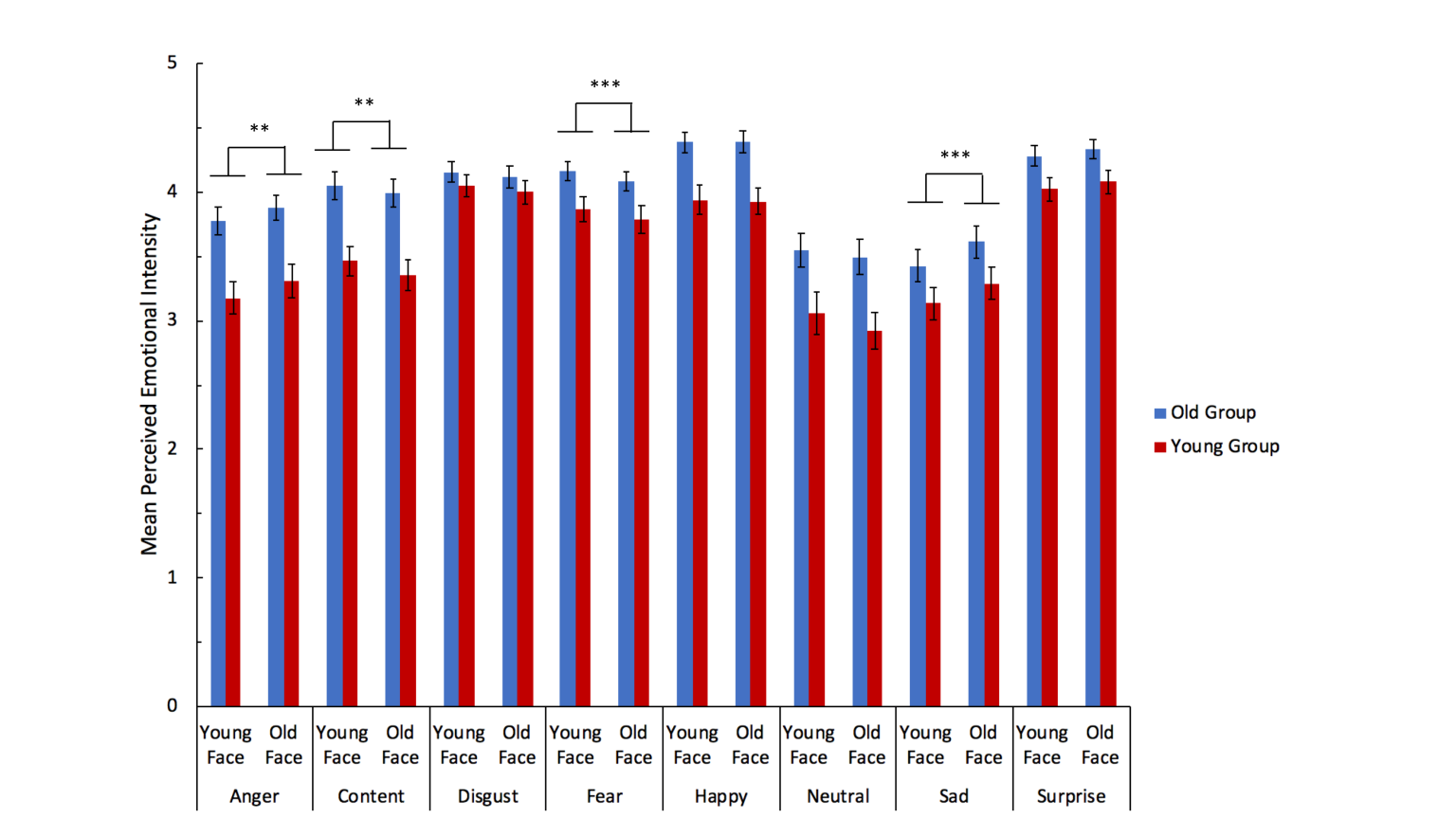
**

**Fig 2. Mean perceived emotional intensity of older and young groups. Blue bars represent old group, red bars represent young group.** 1) Raters’ overall mean perceived intensity for Content and Fear were significantly higher from young faces than from older faces; whereas raters’ overall mean perceived intensity for Anger and Sadness were significantly higher from older faces than from young faces. 2) Older raters’ mean perceived intensity was significantly higher than younger raters on facial expressions of Anger, Content and Happiness, not on other facial expressions. **(**Note: Error bars represents S.E. *** represents *p* <.001. ** represents *p* <.01. * represents *p* <.05.).

***Perceived age***

We also computed the mean difference score (mean estimated age minus actual age) for each facial expression type for each rater, a positive value indicating the perceived age was older than actual age, whereas a negative value indicating the perceived age was younger than actual age. Using these difference scores, a 2 (Rater Age: Young, Older) × 2 (Face Age: Young, Older) × 8 (Facial Expression: Anger, Content, Disgust, Fear, Happiness, Neutral, Sadness, Surprise) mixed ANOVA was performed to examine the effects of Rater Age, Face Age and Facial Expression on modulating the perceived age (Table 4 and Fig 3 present the results).

**Table 4. Results of ANOVA identifying the effects of facial expression, face age, and rater age (young vs. old) on perceived age.**

The main effect of Rater Age was significant, which was due to older raters’ estimated ages (*M* = 5.09, *SD* = 3.72) being higher than young raters (*M* = 0.03, *SD* = 3.54). The main effect of Facial Expression was significant, pairwise comparisons (all with Bonferroni correction, Fig 3) revealed that overall mean perceived ages from Happiness, Content and Neutral faces were significantly younger than from negative facial expressions such as Sadness (vs. Happiness, *p* < .001 , *d* = 1.195; vs. Content, *p* < .001 , *d* = 1.247; vs. Neutral, *p* < .001 , *d* = 1.820), Anger (vs. Happiness, *p* < .001 , *d* = 1.363; vs. Content, *p* < .001 , *d* = 1.343; vs. Neutral, *p* < .001 , *d* = 2.046), Disgust (vs. Happiness, *p* < .001, *d* =1.152 ; vs. Content, *p* < .001, *d* = 1.167; vs. Neutral, *p* < .001 , *d* = 1.821), and Fear (vs. Happiness, *p* < .001 , *d* = 2.432; vs. Content, *p* < .001 , *d* = 2.210; vs. Neutral, *p* < .001 , *d* = 2.651), except Surprise. Interestingly, the overall mean perceived ages from Happiness (*M* =2.12, *SD* = 4.37) (*p* = .014 , *d* =.799) and Content (*M* = 2.10, *SD* = 4.30) (*p* < .001, *d* = 1.058) faces were significantly older than from Neutral faces (*M* = 1.71, *SD* = 4.21). The overall mean difference score of Happiness faces did not significantly differ from Content faces (Fig 3).

In addition, the main effect of Face Age was significant, indicating that the differences between estimated and actual age was greater for young faces (*M* = 6.86, *SD* = 3.27) than for older faces (*M* = -1.74, *SD* = 3.99), regardless of raters’ age or type of facial expression. The interaction between the Facial Expression × Rater Age interaction was significant, we can see that older raters’ higher estimation of face age was more pronounced on Fear (older raters: *M* = 6.34, *SD* = 3.81, young raters: *M* = .64, *SD* = 3.64) and Sad (older raters: *M* = 5.40, *SD* = 3.67, young raters: *M* = .17, *SD* = 3.26) compared to young raters. Independent t-tests (with Bonferroni corrections*)* revealed that older rater’s overall mean difference scores were significantly higher than younger raters across all types of facial expressions. The interaction of Facial Expression × Face Age was significant. Pairwise comparisons (with Bonferroni correction) revealed that the overall perceived age for young faces was significantly higher than for older faces when identifying all facial expressions (p < .001 across all facial expression conditions), and this pattern of results were more pronounced on Fear (older faces: *M* = -1.35, *SD* = 4.79, young faces: *M* = 8.07, *SD* = 4.68) and Surprise (older faces: *M* = -2.35, SD = 4.63, young faces: M = 6.76, SD = 4.47). The three-way interaction between Facial Expression × Face Age × Rater Age was significant. Both older and younger raters’ perceived age were highest on Fear and lowest on Neutral, however, this difference gap (highest-lowest) was more pronounced in older raters compared to young raters. Pairwise comparisons (with Bonferroni correction) revealed that older raters’ mean perceived ages were significantly higher than younger raters on both young and older faces across all facial expressions (see Table 5 and Fig 3).

**Table 5. Comparisons older and young raters’ mean difference score (mean estimated age minus actual age) on all types of images.**

**N.B. Reported p values are uncorrected, comparisons which reached significance following Bonferroni corrections are marked (*)**

**
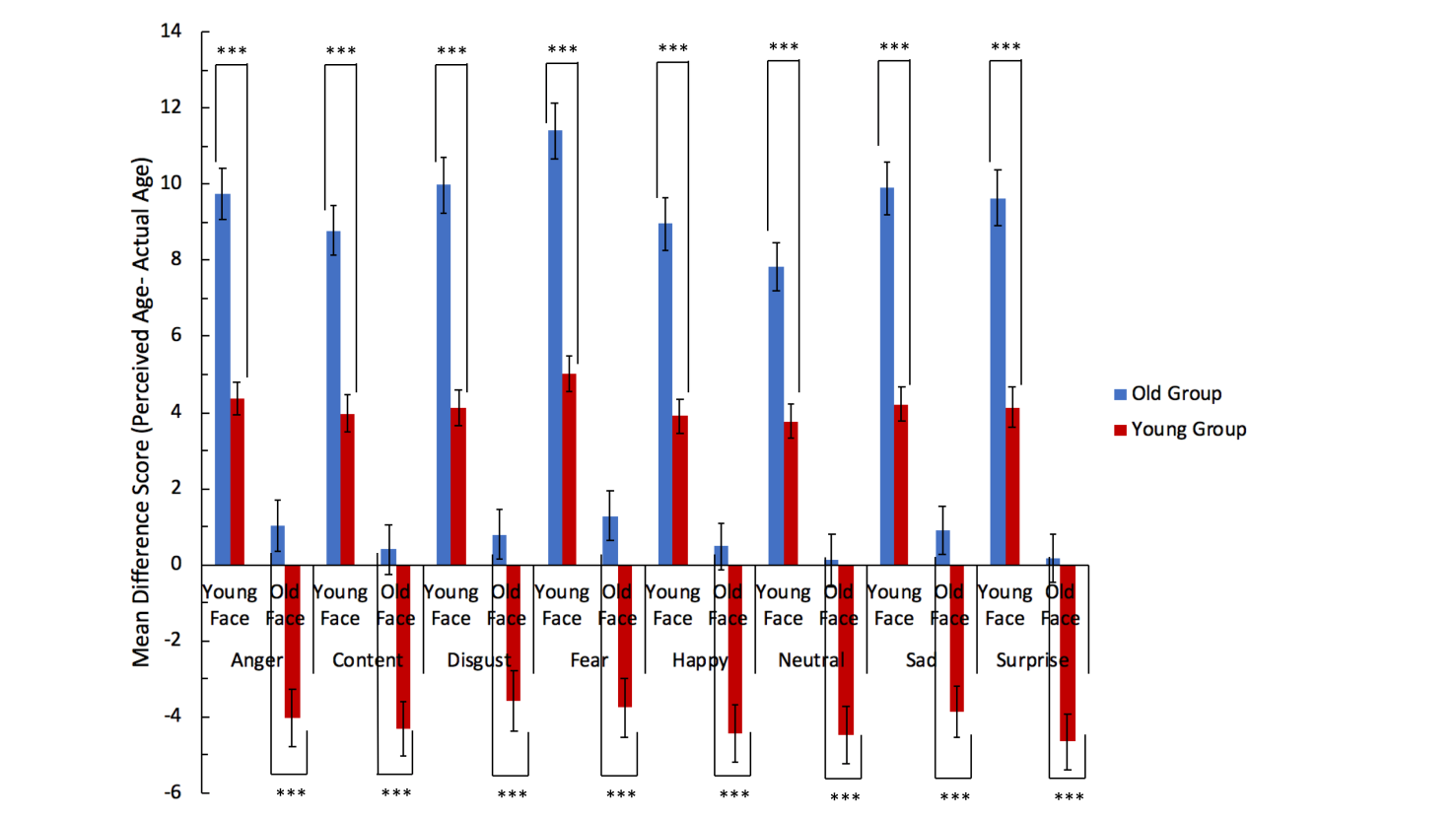
**

**Fig 3. Mean difference score (mean perceived age – mean actual age) of older and young groups on different facial expressions. Blue bars represent old group, red bars represent young group.** A positive value indicating the perceived age is older than actual age, whereas a negative value indicating the perceived age is younger than actual age. 1) The overall mean perceived ages from Happiness, Content and Neutral faces were significantly younger than from negative facial expressions such as Sadness, Anger, Disgust, and Fear, except Surprise. In addition, the overall mean perceived ages from Happiness and Content faces were significantly older than from Neutral faces, the overall mean difference score of Happiness faces did not significantly differ from Content faces. 2) Older raters’ mean perceived ages were significantly higher than younger raters on both young and older faces across all facial expressions. **(**Note: Error bars represents S.E. *** represents *p* <.001. ** represents *p* <.01. * represents *p* <.05.).

***Discussion***

The validation results have shown that older raters’ accuracy was significantly lower than younger adults at identifying Anger, Disgust, Fear, Surprise, Happiness, Neutral, Sadness and Surprise from either young/older faces, or both; while older raters’ identification accuracy for Content facial images were similar to young adult raters. This finding adds strength to prior studies reporting that older people have declined facial emotional perceptual ability [see a review 19]. In addition, raters’ overall accuracy for identifying young faces was significantly higher than for old faces, which is in parallel with prior studies stating that the wrinkles and folds of older faces can reduce the signal clarity of facial expressions [45, 46].

In terms of perceived emotional intensity, older raters’ mean perceived intensity was significantly higher than younger raters on facial expressions of Anger, Content and Happiness, but not on other facial expressions. This result partially supports ‘age-related positivity effect’, where older people tend to devote more attention on positive aspect of information that promote positivity, emotional balance, and well-being [socio-emotional selectivity theory - 47]. In addition, raters’ overall mean perceived intensity for Content and Fear were significantly higher from young faces than from older faces, whereas raters’ mean perceived intensity for Anger and Sadness were significantly higher for older faces than for young faces. This might be due to the winkles and folds of older adult faces mimicking or exaggerating the muscle movements of negative facial emotional expressions (e.g., wrinkles between eyes), whereas the facial features such as fuller cheeks and larger eye sizes (no extra folds on eyelids) of young faces mimics facial expression Content (lifted cheek muscles) and Fear (e.g., enlarged eyes). Part of these results is consistent with prior findings stating that facial expression of Anger is easier to detect from older faces than from young faces [48], whereas smile/Happiness is easier to be perceived from young faces than from older faces [49].

In terms of perceived age, the results have shown that both young and older adult raters were more accurate at estimating their own-age faces, which suggests that the ‘own-age bias’ involved in face processing extends to the perception of face age. The perceived age from faces with Happiness, Content and Neutral facial expressions were significantly younger than from faces displaying negative expressions such as Sadness, Anger, Disgust, and Fear (except Surprise). Interestingly, perceived age from faces with Happiness and Content facial expressions were significantly older than from Neutral faces, this result contradicts our normal perception – smiling makes you look younger. However, this unexpected finding awaits further replications to confirm.
